# Changes in microRNA expression associated with preeclampsia: a systematic review

**DOI:** 10.1590/1414-431X2025e13988

**Published:** 2025-05-30

**Authors:** A.C.S. Lopes, A.A. de Macedo, F.S. Mendes, I.M. Costa, L.M.S. Dusse, P.N. Alpoim

**Affiliations:** 1 Departamento de Análises Clínicas e Toxicológicas, Faculdade de Farmácia, Universidade Federal de Minas Gerais, Belo Horizonte, MG, Brasil

**Keywords:** Preeclampsia, MicroRNAs, Biomarkers, Systematic review

## Abstract

Preeclampsia (PE) is a disease of pregnancy characterized by the new onset of hypertension accompanied by proteinuria and/or other signs of maternal organ dysfunction that manifests after 20 weeks of gestation. MicroRNAs (miRNAs) are small non-coding RNAs (19-25 nucleotides) that function in the post-transcriptional regulation of gene expression. Many studies have suggested that different microRNA expression profiles may be associated with the development of PE. Hence, this study aims to report differentially expressed microRNAs that may be associated with the pathogenesis of PE and investigate whether different miRNA expression profiles are associated with different PE classifications and different phases of pregnancy. The bibliographic search was conducted from September 2021 to August 2024 and was performed on MEDLINE/PubMed, EMBASE, and Web of Science. This systematic review followed the methodological guidelines of the Cochrane Collaboration Manual for Systematic Intervention Reviews and was written according to the Preferred Reporting Items for Systematic Reviews and Meta-Analyses (PRISMA). Of the 1362 studies identified, 263 articles were selected as the sample of this study. The most frequently cited upregulated microRNAs were: miR-210, miR-155, miR-518b, miR-181a, miR-125b, miR-183, and miR-16. The most frequently cited downregulated microRNAs were: miR-363, miR-18a, miR-144, miR-149, miR-16, miR-18b, and miR-195. This study will serve as a reference to guide future experimental research. In addition, knowledge of the expression profiles of microRNAs associated with PE can help in the development of new protocols for early prediction of the disease.

## Introduction

Preeclampsia (PE) is the main cause of maternal and perinatal morbidity and mortality (1). It is characterized by the new onset of hypertension accompanied or not by proteinuria and/or signs of maternal organ dysfunction that manifests after 20 weeks of gestation (2). PE results from abnormal placentation, causing insufficient utero-placental blood perfusion and ischemia (2). PE can be classified according to the time of disease onset as early-onset or late-onset PE (EOPE/LOPE) if it occurs before or at/after 34 weeks of gestation, respectively (3).

MicroRNAs (miRNAs or miRs) are small non-coding RNAs (19-25 nucleotides) that function in the post-transcriptional regulation of gene expression. Many studies have suggested that different miRNA expression profiles may be associated with the development of PE (1-4). MiRNAs play key roles in regulating diverse biological processes and have aroused great interest in the diagnosis and monitoring of various diseases (2). Several studies have suggested that these molecules participate in the pathogenesis of PE by regulating common pathways such as hypoxia, ischemia, angiogenesis, and metabolism (3). In addition, due to their stability in plasma, the evaluation of miRNAs may be useful in the early detection of PE (2).

Although PE has been extensively studied, there is no cost-effective laboratory marker that indicates an increased risk of PE in the pre-symptomatic stage, which could improve the management of pregnant women, help prevent other associated complications, and accurately monitor PE and pregnancy progression. In this context, previous studies have been carried out aiming to understanding the microRNAs expression in preeclamptic women (4,5). It is important to emphasize that previous systematic reviews have been carried out on the same topic (4). However, the data must be updated, since many experimental studies have been published in recent years that take into account molecular biology development, which can lead to new data interpretations.

To the best of our knowledge, no systematic review has been conducted to date to assess miRNAs expression patterns in different PE classifications (EOPE, LOPE) or in different gestational trimesters. Hence, this systematic review aims to describe differentially expressed miRNAs - in plasma and/or placenta - that may be up- or downregulated in PE and to identify possible microRNA expression profiles in different PE classifications and pregnancy trimesters.

## Material and Methods

This systematic review followed the methodological guidelines of the Cochrane Handbook for Systematic Reviews of Interventions (6), and the results are presented according to the Preferred Reporting Items for Systematic Reviews and Meta-Analyses (PRISMA) (7). The search stage, data extraction, and risk of bias assessment were conducted by two researchers independently, with disagreements resolved by peers.

The study protocol was registered in the International Prospective Register of Systematic Reviews (PROSPERO) on September 18, 2021, under the registration identification number CRD42021274280.

### Search question

The search question included Population (pregnant women), Variable (preeclampsia occurrence), Outcome (microRNAs expression levels), and Study (analytical and observational studies). This systematic review aimed to answer the following question: which differentially expressed miRNAs (in plasma or placenta) are associated with PE occurrence?

### Bibliographic search

The bibliographic search was performed in June 2021 and updated in June 2022 and July 2024 in six databases: MEDLINE/PubMed, EMBASE, Web of Science, Cochrane, Scielo, and LILACS. The keywords used were preeclampsia and microRNAs and variations of these terms (the complete search strategy is available in the Supplementary Table S1). No language or publication date filter was used, but articles in Chinese (n=4), Russian (n=3), and Czech (n=1) were excluded because no member of the research group was able to accurately translate them.

As inclusion criteria, we selected: 1) case-control and cohort studies that determined miRNA expression in blood and/or placenta in pregnant women with EOPE or LOPE and in normotensive pregnant women during the three gestational trimesters. PE was defined as new onset elevation of blood pressure (BP ≥140/90 mmHg) at least 2 h apart at rest, after 20 weeks of pregnancy with evidence of end organ damage such as proteinuria and/or clinical symptoms (swelling of the hands and face, headache, visual disturbances, abdominal pain, nausea, and vomiting). Proteinuria was defined as ≥300 mg/24 h and/or a dipstick reading of 2+. EOPE and LOPE are distinguished based on the gestational age of symptom manifestation: before (EOPE) or after (LOPE) 34 weeks (3).

The exclusion criteria were: 1) Full text was unavailable (n=23); 2) The diagnostic criteria for PE were not clearly described (n=97); 3) The biological sample evaluated was other than blood or placenta. Studies that analyzed umbilical cord blood, culture cells, or exosomal microRNAs were also excluded (n=53); 4) MicroRNA expression levels were not assessed or not clearly reported. Studies that did not report which method was used to assess microRNA levels were also excluded (n=78); 5) The case group was not preeclamptic pregnant women. Studies evaluating women with gestational hypertension (GH), pregnancy-induced hypertension (PIH), fetal growth restriction (FGR), history of PE, superimposed PE or HELLP syndrome were not included (n=41); 6) The study did not include a control group with normotensive pregnant women (n=6); 7) The study did not describe the number of preeclamptic and/or normotensive participants (n=20); 8) The publication was retracted or was just a corrigendum (n=6); 9) The article could not be translated (n=8) because it was in Chinese (n=4), Russian (n=3), or Czeck (n=1); 10) The study was carried out on animals (n=10); 11) The study methodology involved bioinformatics analysis (n=11); and 12) The publication type was a review article, editorial, or commentary (n=7).

We also performed a manual search for studies in the citations of publications primarily included in this review. In addition, we searched for congress abstracts on Embase, using the same keywords used to search for articles. To obtain incomplete data on selected articles, attempts were made to contact the authors. If this was unsuccessful, the study was excluded.

### Data extraction

Study selection and data extraction were carried out by two independent reviewers (ACSL and AAM) using the software Rayyan (for duplication detection and exclusion and selection by title and abstract) and Microsoft Excel (for data extraction and analysis). Disagreements were resolved by a third examiner (PNA).

The following data were extracted from the studies: authors, title, publication year, country, type of study (case-control or cohort), PE diagnosis criteria, number of participants, biological sample (blood and/or placenta), clinical data for PE and normotensive pregnant women [maternal age, gestational age at sampling and delivery, systolic and diastolic blood pressure, proteinuria levels, platelets count, serum creatinine levels, serum aminotransferases activity (AST: aspartate aminotransferase and ATL: alanine aminotransferase), body mass index (BMI), adverse outcomes or complications, fetal birth weight], PE classification (early/late, mild/severe), analyzed microRNAs, microRNA expression profiles, and methods used for microRNA detection. In addition, quantitative data regarding microRNA expression levels were extracted when available.

Studies that did not present at least one of the following information were excluded: authors, title, country, year of publication, type of study, biological sample, number of participants, PE diagnosis criteria, evaluated microRNAs, microRNA expression status (upregulated, downregulated), and method used for microRNA quantification.

### Methodological quality

To assess the quality of case-control and cohort studies we used the Newcastle-Ottawa Quality Assessment Scale (NOS) for case-control studies (8). Similarly, this step was performed by two independent reviewers and disagreements were resolved by a third reviewer. This scale includes eight items divided into three components: 1) selection, 2) comparability, and 3) exposure. Studies were assigned a score from 0 to 9, with studies scoring ≥8 classified as high quality, 5 to 7 as moderate, and ≤4 as low quality.

### Data synthesis and analysis

Stratified analyzes were performed by biological sample (blood and/or placenta) and, when possible, by PE classification (EOPE; LOPE) and gestational trimester in which the biological sample was obtained (first, second, and third trimesters). We analyzed which microRNAs were differentially expressed in each group and assessed the correlation with their biological role.

## Results

### Bibliographic search

**Figure 1:**
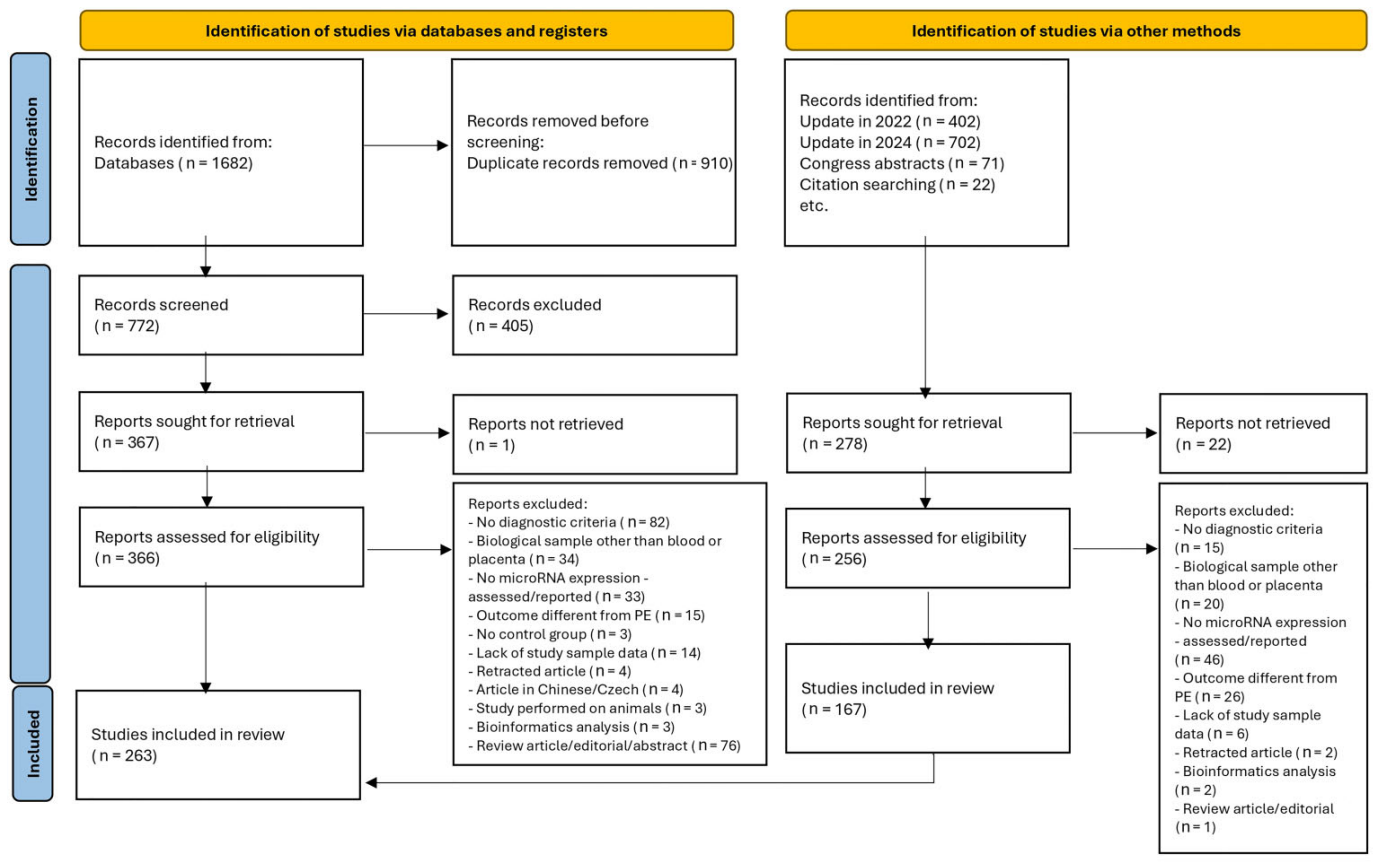
PRISMA 2020 flow diagram for new systematic reviews, which included searches of databases, registers, and other sources. From: https://doi.org/10.1136/bmj.n71. For more information, visit: http://www.prisma-statement.org/

The articles found on each database (PubMed, Embase, Web of Science, and LILACS) were imported into the online platform of Rayyan (9), which was used for the selection of eligible studies based on their titles and abstracts. Studies that were not excluded in this first stage were read in full, and data of the selected articles were included in a Microsoft Excel spreadsheet. The article selection was carried out until June 2021. However, two updates were performed (in June 2022 and July 2024). Finally, 263 studies were selected for the sample of this systematic review (Supplementary Table S2). The selection process and the reasons for article exclusion are described in a flowchart (Figure 1). Several articles met more than one exclusion criterion.

### Data analysis

Data analysis was performed considering all studies or by subgroups. For this reason, the results are presented in separate topics in order to analyze the findings in a stratified manner.

#### General analysis

Most of the studies included in this systematic review were published in China (n=164), followed by the USA (n=15) and Turkey (n=9). The list of the articles published in each country is available in Supplementary Table S3.

The year of publication of the studies ranged from 2007 to July, 2024, with an increase in published articles on the topic during this period.

Different diagnosis criteria were used for deciding on the inclusion of PE women in the studies. The most cited criteria were based on the American College of Obstetricians and Gynecologists (ACOG) Guidelines (n=78), the International Society for the Study of Hypertension in Pregnancy (ISSHP) (n=24), and Williams Obstetrics (23rd edition) (n=8). Additionally, several publications described the criteria without citing any organization. Among those, the majority of studies used the following criteria: “hypertension and proteinuria after the 20th week of pregnancy” (n=70). Some studies considered only “hypertension and proteinuria” without defining a gestational age (n=45), and others also considered “organ system dysfunction or fetus growth restriction” accompanied or not by proteinuria (n=20). In disagreement with current guidelines for the diagnosis of PE, some studies considered pregnant women who manifested only hypertension or proteinuria (n=9) or developed the symptoms after the 28th (n=1) or 34th week of gestation (n=1). Additional criteria used were: the Royal College of Obstetricians and Gynaecologists Guidelines of 2005, 8th edition Obstetrics and Gynecology - 2006, 9th edition of Obstetrics and Gynecology, ISSHP 2018 and ACOG 2020, ISSHP and ACOG 2002, Society of Obstetrics and Gynecology of the Chinese Medical Association 2020 Guideline for Diagnosis and Treatment of Hypertensive Disorders in Pregnancy, and Diagnosis Guidelines of the Expert Committee of Severe Preeclampsia.

Among the studies included in this systematic review, 220 were case-control studies, 26 were cohort studies, and 17 were a mixture of both types of methodological design.

Regarding the biological samples analyzed, 72 studies evaluated microRNA expression levels in blood samples only, 165 studies evaluated microRNA expression in placental samples only, and 26 studies analyzed both types of biological samples for microRNA expression.

The number of participants in each study ranged from 3 to 224 for preeclamptic women and from 3 to 421 for normotensive pregnant women.

The most cited outcome or complication associated with PE was fetal birth weight (n=146), followed by placental weight (n=18), caesarean (n=17), mode of delivery (n=18), fetal growth restriction [intrauterine growth restriction (IUGR) (n=12) and small for gestational age (SGA) (n=4)], APGAR (1 and 5 min) (n=12), neonatal size (in centimeters) (n=5), induction of labor (n=2), still births or neonatal deaths (n=2), perinatal mortality (n=1), live births (n=1), miscarriage (n=1), placental conditions [decidual vasculopathy (n=1), infarct (n=1), placenta abruption (n=1), villous abnormal development (n=1)], preterm delivery (n=2), anesthesia (n=1), fetal distress (n=1), maternal weight gain (n=1), signs of HELLP (n=2), fetal sex (n=2), and anemia (n=1). Generally, 151 studies cited at least one outcome, while 112 studies cited none.

A total of 59 studies classified preeclamptic women according to the gestational age of PE symptom onset. Of these, 19 included only EOPE pregnant women, 9 included only LOPE pregnant women, and 31 included both EOPE and LOPE pregnant women. Additionally, 204 studies did not mention the classification of preeclamptic women according to gestational age.

Most studies (n=252) used the real-time reverse transcription polymerase chain reaction (RT-qPCR) for detection and quantification of microRNAs. The second most used technique was the microarray (n=29), and several studies used both techniques (RT-qPCR and microarray) (n=28). Other methodologies employed were variations on sequencing techniques (n=9), PCR methods other than RT-qPCR (n=5), *in situ* hybridization (n=4), chromogenic *in situ* hybridization (CISH) (n=1), and enzyme-linked immunosorbent assay (n=1).

A total of 312 microRNAs were found to be upregulated. The most frequently cited upregulated microRNAs were: miR-210 (n=32) (2,10-40), miR-155 (n=16) (2,3,10,14,17,30,41-50), miR-518b (n=9) (23,24,38,51-56), miR-181a (n=9) (21,24,37,38,57-61), miR-125b (n=6) (62-67), miR-183 (n=5) (20,30,68-70), and miR-16 (n=5) (21,59,71-73). Conversely, 249 microRNAs were detected as downregulated. The most frequently cited downregulated microRNAs were: miR-363 (n=4) (21,24,38,74), miR-18a (n=5) (2,24,38,74,75), miR-144 (n=6) (2,23,38,58,76,77), miR-149 (n=3) (74,78,79), miR-16 (n=3) (58,74,80), miR-18b (n=4) (21,38,81,82), and miR-195 (n=6) (24,38,83,84,86). Surprisingly, miR-210, which is the most cited upregulated microRNA in PE, was also cited as downregulated in one study (87). The full list of upregulated and downregulated microRNAs is available in Supplementary Tables S4 and S5.

#### Placental tissues

Placental microRNA expression levels were investigated in 191 studies (72.07%). Among these, 26 also assessed circulating levels. A total of 186 microRNAs were found to be upregulated in PE placenta samples, the most frequently cited being: miR-210 (n=21) (13,15,16,18,19,21,22,24-26,28-30,32-39), miR-155 (n=10) (3,30,41-46,48,50), miR-181a (n=7) (21,24,37,38,57,59,60), miR-182* (N=3) (25,30,37), miR-183 (n=4) (30,68-70), miR-16 (n=4) (21,59,71,73), miR-20b (n=5) (21,59,71,73), and miR-20a (n=4) (21,26,89,90). A total of 173 microRNAs were detected as downregulated in PE placenta samples, the most frequently cited being: miR-363 (n=4) (21,24,38,74), miR‐18b (n=4) (21,38,81,82), and miR-195 (n=5) (24,38,83,84,86). The full list of upregulated and downregulated placental microRNAs is available in Supplementary Tables S4 and S5.

#### Blood samples

Blood microRNA expression levels were investigated in a total of 98 studies (36.98%). Among these, 26 also assessed microRNA placental levels. A total of 163 microRNAs were found to be upregulated in PE blood samples, the most frequently cited being: miR-210 (n=13) (2,10-12,14,17,20,23,24,27,31,40,49), miR-155 (n=7) (2,10,14,17,41,45,47), miR-518b (n=7) (23,24,51-56), and miR-27b-3p (n=4) (91-94). A total of 83 microRNAs were detected as downregulated in PE blood samples, the most frequently cited being: miR-16 (n=3) (58,74,80), miR-18a (n=3) (2,24,74), miR-144 (n=3) (23,58,77), and miR-363 (n=2) (24,74). The full list of upregulated and downregulated circulating microRNAs is available in Supplementary S3 and S4.

#### Early-onset preeclampsia (EOPE)

Fifty studies (18.87%) assessed microRNA expression levels in EOPE. Among these, 31 also investigated microRNA expression profiles in LOPE. A total of 38 microRNAs were found to be upregulated in EOPE, the most frequently cited being: miR-518b (n=5) (51,52,54-56), miR-125b (n=3) (62,65,66), and miR-155 (n=3) (17,42,48). A total of 33 microRNAs were detected as downregulated in EOPE, the most frequently cited being: miR-16 (n=2) (74,80). The full list of upregulated and downregulated microRNAs in EOPE is available in Supplementary Tables S4 and S5.

In studies that analyzed placental samples from EOPE pregnant women, 22 microRNAs were found to be upregulated (3,42,60,95-105), while 15 were found to be downregulated (3,22,42,60,74,97,104,106-108). Except for miR-22 (cited by two different studies as upregulated (100,105)), none of the other microRNAs were cited by more than one study (Table 1).

**Table 1:** Differentially expressed microRNAs in early-onset preeclampsia (EOPE) placentas reported by studies (in parenthesis).

Upregulated	Downregulated
**miR-22** 100,105	miR-135a 108
miR-22-3p 105	miR-140-5p 107
miR-33b-3p 98	miR-148b* 97
miR-124* 3	miR-149 74
miR-126 96	miR-149-5p 104
miR-130b 3	miR-181a 60
miR-133b 102	miR-223-3p 22
miR-137 101	miR-224-5p 22
miR-155 3,42,48	miR-363 74
miR-181a-2-3p 98	miR-520a-3p 106
miR-210-5p 98	miR-544 3
miR-331-5p 104	miR-937 97
miR-367* 98	miR-1301 22
miR-372-3p 104	miR-3907 97
miR-383 3	miR-3942 3
miR-423-3p 3	
miR-431 3	
miR-452 95	
miR-518a-5p 3	
miR-519a 60	
miR-663 99	
miR-4743-5p 104	

miR-22 was found to be upregulated by two studies (in bold type).

In studies that analyzed blood samples from EOPE pregnant women, 15 microRNAs were found to be upregulated (17,51,52,54,62,66,80,105,109-112), while 14 were found to be downregulated (17,66,74,80,85,104,113). Except for miR-518b (cited by two different studies as upregulated (51,52)), miR-16 (74,80), and miR-195 (n=2) (cited by two different studies as downregulated (24,85)), none of the other microRNAs were cited by more than one study (Table 2).

**Table 2:** Differentially expressed circulating microRNAs in early-onset preeclampsia (EOPE) reported by studies (in parenthesis).

Upregulated	Downregulated
miR-22 105	**miR-16** 74,80
miR-22-3p 105	miR-18a 74
miR-29b 112	miR-31 113
miR-125b 65	miR-125a-5p 17
miR-126 111	miR-126# 66
miR-143 66	miR-127 66
miR-155 17	miR-149 74
miR-192 66	**miR-195** 24,85
miR-200c 80	miR-221 66
miR-210 17	miR-331-5p 104
miR-320a 109	miR-363 74
**miR-518b** 54,55	miR-372-3p 103
miR-574-5p 110	miR-942 118
miR-1972 110	miR-4743-5p 104
miR-4793-3p 110	

miR-518b was found to be upregulated by two studies; miR-16 and miR-195 were found to be downregulated by two studies (in bold type).

#### Late-onset preeclampsia (LOPE)

Forty studies (15.09%) assessed microRNA expression levels in LOPE. Among these, 31 also investigated microRNA expression profiles in EOPE. A total of 56 microRNAs were found to be upregulated in LOPE (3,22,36,45,52,54,58-60,67,99,101,103,104,114-116), the most frequently cited being: miR-181a (n=3) (58-60), miR-210 (n=2) (22,36), and miR-1183 (n=2) (3,114). A total of 26 microRNAs were detected as downregulated (42,55,58,59,67,83,91,104,113,117,118), and none of them was cited by more than one study. The full list of upregulated and downregulated microRNAs in LOPE is available in Supplementary S3 and S4.

In studies that analyzed placental samples from LOPE pregnant women, 29 microRNAs were found to be upregulated (3,22,36,45,59,60,99,101,103,104,114-116), while 21 were found to be downregulated (42,48,59,67,83,91,104,116). Except for miR-181a (59,60), miR-210 (22,36), and miR-1183 (3,114), which were cited by two different studies as upregulated, and miR-146a (42,48) and miR-155 (42,48), which were cited by two different studies as downregulated, none of the other microRNAs were cited by more than one study (Table 3).

**Table 3:** Differentially expressed microRNAs in late-onset preeclampsia (LOPE) placentas reported by studies (in parenthesis).

	Upregulated	Downregulated
let-7b* 114	miR-378c 99	let-7c-5p 116
let-7f-1* 114	miR-383 3	miR-1 28
miR-16 59	miR-425* 114	miR-27b-3p 91
miR-23c 114	miR-513c-5p 115	miR-125b 67
miR-26b 59	miR-514b-3p 99	miR-127-3p 116
miR-29b 59	miR-519b-3p 59	**miR-146a** 42,48
miR-126-3p 115	miR-892c-3p 99	miR-149-5p 104
miR-137 101	**miR-1183** 3,114	**miR-155** 42,48
miR-145-5p 115	miR-4743-5p 104	miR-186-5p 91
miR-155 45		miR-195 83
**miR-181a** 59,60		miR-214 59
miR-193b-5p 115		miR-423-5p 59
miR-195 59		miR-491-5p 59
**miR-210** 22,36		miR-508-5p 59
miR-222 59		miR-519a-3p 116
miR-296-5p 115		miR-532-3p 59
miR-335 59		miR-532-5p 116
miR-371a-5p 115		miR-539-5p 116
miR-372-3p 103		miR-612 59
		miR-629-5p 116
		miR-658 59

miR-181a, miR-210, and miR-1183 were found to be upregulated by two studies; miR-146a and miR-155 were found to be downregulated by two studies (in bold type).

In studies that analyzed blood samples from LOPE pregnant women, 14 microRNAs were found to be upregulated (52,54,58), while 5 were found to be downregulated (58,113,117) (Table 4). None of these microRNAs was cited by more than one study.

**Table 4:** Differentially expressed circulating microRNAs in late-onset preeclampsia (LOPE) reported by studies (in parenthesis).

	Upregulated	Downregulated
miR-24 58	miR-151-3p 58	miR-16 58
miR-26a 58	miR-181a 58	miR-21 113
miR-30d 58	miR-221 58	miR-23b-5p 117
miR-103 58	miR-342-3p 58	miR-99b-5p 117
miR-130a 58	miR-425 58	miR-144 58
miR-130b 58	miR-518b 52	
miR-145 58	miR-574-5p 58	

#### First trimester

Twelve studies (4.53%) assessed microRNA expression levels in the first trimester of pregnancy (from 1 to 13 weeks). Among these studies, 11 investigated microRNA levels in blood samples, and two analyzed placental tissues. A total of 18 microRNAs were found to be upregulated (53,58,64,66,119,120) and 37 microRNAs were found to be downregulated (64,66,119) in the first trimester of pregnancies that progressed to PE. Except for miR-125b (63,66), which was cited by two different studies as upregulated, none of the other microRNAs were cited by more than one study (Table 5).

**Table 5:** Differentially expressed microRNAs in first trimester reported by studies (in parenthesis).

	Upregulated	Downregulated
let-7a-5p 6	ebv-miR-BART1-5p 119	miR-642b-3p 119
miR-15a-5p 64	hiv1-miR-TAR-3p 119	miR-892b 119
miR-15a-5p 64	miR-22-5p 64	miR-942 66
miR-92a-1-3p 64	miR-92a-2-5p 119	miR-1273c 119
miR-106a 64	miR-93-5p 64	miR-2392 119
**miR-125b** 63,64	miR-107 119	miR-3064-5p 119
miR-130a-3p 64	miR-126# 66	miR-3171 119
miR-143 66	miR-126-3p 64	miR-3184-5p 119
miR-146b-5p 120	miR-127 66	miR-3649 119
miR-191-5p 64	miR-188-3p 119	miR-4264-5p 64
miR-192 66	miR-203a-3p 119	miR-4329 119
miR-320a 119	miR-204-3p 64	miR-4432 119
miR-374a-5p 64	miR-211-5p 119	miR-4482-3p 119
miR-517-5p 53	miR-221 66	miR-4498 119
miR-518b 53	miR-365a-3p 64	miR-4758-5p 119
miR-520h 53	miR-369-3p 119	miR-5000-5p 119
miR-574-5p 64	miR-424-3p 119	miR-5009-3p 119
miR-1304-5p 119	miR-506-5p 119	miR-5582-3p 119
miR-5002-5p 119	miR-559-5p 64	

miR-125b was found to be upregulated by two studies.

#### Second trimester

Twenty-three studies (8.68%) assessed microRNA expression levels in the second trimester of pregnancy (from 14 to 27 weeks). Among these, 17 analyzed blood samples only, one analyzed placental tissue and five analyzed both blood and placenta samples. One microRNA (miR-204-5p) was found to be upregulated (121) and 4 (miR-133a, miR-206, miR-210, miR-942) were downregulated (87,118,122) in the second trimester of pregnancies that progressed to PE. No microRNA was cited by more than one study.

#### Third trimester

One hundred and ninety-five (74.58%) studies assessed microRNA expression levels in the third trimester of pregnancy (from 28 weeks onwards). A total of 182 microRNAs were found to be upregulated in third trimester samples from women who developed PE, the most frequently cited being: miR-210 (n=23) (10-15,17,19-22,25,27-30,32-36,38,40), miR-155 (n=12) (3,10,14,17,30,42-46,49,50), miR-16 (n=5) (21,59,71-73), miR-181a (n=6) (21,38,57-59,61), and miR-182* (n=3) (25,30,37). A total of 166 microRNAs were detected as downregulated in third trimester samples from women who developed PE, the most frequently cited being: miR-363 (n=3) (21,38,74), miR-1 (n=3) (28,38,67), miR-16 (n=3) (58,74,80), miR-18a (n=4) (24,38,74,75), miR-18b (n=4) (21,38,81,82), miR-144 (n=4) (38,58,76,77), miR-149 (n=3) (74,78,79), and miR-195 (n=4) (38,83,84,86). The full list of upregulated and downregulated microRNAs in the 3rd trimester is available in Supplementary Tables S4 and S5.

### Methodological quality

According to the Newcastle-Ottawa Quality Assessment Scale (NOS) for Case Control Studies (8), seven studies scored one point, 22 scored two points, nine scored three points, 26 scored four points, 62 scored five points, 61 scored six points, 32 scored seven points, 41 scored eight points, and three scored nine points.

## Discussion

Despite the extensive efforts of researchers around the world, the etiology of PE is not fully understood. Although immunological, inflammatory, and genetic factors and placental ischemia have been implicated in its pathogenesis, the exact mechanism underlying PE remains difficult to understand. Induced preterm labor with complete removal of the placenta remains the only curative treatment option for the management of PE, but this does not guarantee complete resolution of postpartum complications (2), since both women who develop PE and their neonates are at increased risk of developing cardiovascular disease and metabolic syndromes (such as type II diabetes) later in life (3).

MicroRNAs play a role in post-transcriptional regulation of gene expression through translational inhibition or degradation of messenger RNA. These molecules play key roles in the regulation of diverse biological processes, including cell differentiation, apoptosis, and development (4) and have triggered great interest in the diagnosis and monitoring of various conditions, including cancer, autoimmune, inflammatory, and neurological diseases (10). As the placenta is an important element in the development of PE, miRNAs may be implicated in the pathogenesis of the disease, regulating pathways associated with angiogenesis, hypoxia, ischemia, and metabolism (3).

### MicroRNAs as tools for early PE diagnosis

Currently, no marker can predict PE during the first trimester, when the process of maladaptive placentation begins (66). In this context, PE-associated miRNAs have the potential to serve as viable biomarkers, since several studies demonstrate the deregulation of placental and circulating miRNAs in PE, implying their involvement in the pathogenesis of the disease (1-3). These studies confirmed the differential expression of miRNAs in placental and circulating blood of women with PE compared to controls, which suggests a key role of this epigenetic mechanism in the altered development of placental vascular structure and subsequent cascade of events.

Due to their great stability in biological fluids such as plasma (which may be longer than nine months), the use of miRNAs has a great advantage in the early and non-invasive prediction of PE (2,66). In addition, the routine use of molecular biology techniques, which were previously restricted to research laboratories due to its high cost and complexity, has been increasingly used in clinical practice, especially after the COVID-19 pandemic, whose gold standard diagnosis is mainly established RT-qPCR, the same method used in the detection and quantification of microRNAs (123).

### Most frequently cited upregulated microRNAs

As demonstrated in this study, the most frequently cited microRNA with altered expression levels is miR-210. Being induced by hypoxia-inducible factor-1α during a hypoxia event, miR-210 acts by regulating several hypoxia response pathways, such as cell survival, angiogenesis, mitochondrial metabolism, and DNA repair (2). Winger et al. (124) suggested that miR-210 plays a central role in changing the pattern of trophoblast proliferation, which contributes to the PE pathogenesis. Differential expression of miR-210 was detected in both placenta and blood samples from women with EOPE and LOPE. This is in accordance with the literature, which reports that it is one of the most commonly over-expressed miRNAs in PE. However, it is still unclear whether the differential expression of miR-210 is a cause or a consequence of PE, although this miRNA is one of the main bets for a PE biomarker (1).

Interestingly, when performing the stratified analysis of the data, miR-210 was not one of the microRNAs found to be upregulated in the first and second trimesters of pregnancy, which would be expected due to the mechanisms it regulates. However, it should be noted that some studies did not clearly report the gestational interval in which the samples were collected or included pregnant women with gestational ages that fall into more than one trimester. Therefore, this result does not necessarily imply that miR-210 is not upregulated in the first gestational trimester, but due to unclear results, we cannot confirm this information. In addition, most studies carried out in the first and second trimesters of pregnancy analyzed blood samples, since the collection of placental tissue in early gestation periods increases the risk of miscarriage, being performed only in specific cases and under medical advice.

Other frequently cited upregulated microRNAs have been investigated in *in vitro* studies performed with trophoblastic cells and were shown to act in pathways that play important roles related to PE pathogenesis. According to Gan et al. (17), miR-155 is an essential regulator of endothelium-dependent vasorelaxation, playing a negative regulatory role in the migratory behavior via modulating endothelial nitric oxide synthase (eNOS) (17). MiR-518b belongs to the chromosome 19 microRNA cluster (C19MC), and Jelena et al. (52) claim that its upregulation in preeclamptic placentas may contribute to excessive trophoblast proliferation, which is a common pathological change in PE. Regarding miR-181a, its upregulation may lead to the downregulation of transforming growth factor beta (TGF-β) signaling and upregulation of mitogen-activated protein kinase (MAPK), being associated with elevated IL-6 levels in maternal plasma in preeclamptic women (125).

Yang et al. (65) suggested that miR-125b is involved in proliferation, apoptosis, invasion, and vascular endothelial growth factor (VEGF) production, acting on hypoxia and immune response mechanisms through the production of IL-8 by trophoblast cells. Li et al. (64) reported that elevated miR-125b inhibited trophoblast invasion and angiogenesis, leading to impaired endothelial cell function and poor placentation, contributing to the PE physiopathology. Suo et al. (70) demonstrated that miR-183 inhibited the invasion and migration of HTR-8/SVneo trophoblast cells via targeting matrix metallopeptidase 9 (MMP-9). Thus, miR-183 probably suppresses trophoblast cell proliferation, invasion, and angiogenesis, contributing to the development of PE (69). Likewise, miR-16 is involved in the regulation of proliferation, migration, and invasion of trophoblastic cells (71).

### Most frequently cited downregulated microRNAs

The most frequently downregulated microRNAs were also studied through *in vitro* analyses. miR-363 has been shown to target placental sodium coupled neutral amino acid transporters, leading to a variation in amino acids transport and nutrient transfer, which would culminate in the manifestation of PE (126). MiR-18a is associated with trophoblast cell invasion, likely via inhibition of TGF-β signaling (127), while miR-144 acts in the regulation of proliferation, migration, and invasion of trophoblastic cells (76), as does miR-16 (71). These findings suggest that such microRNAs are essential for maintaining the biological function of trophoblast cells and their downregulation can lead to PE development. In a complementary manner, Dong et al. (113) proposed that miR-31, miR-21, and miR-16 regulate various cellular and developmental processes by targeting genes involved in proliferation, invasion, angiogenesis, apoptosis, and immune tolerance.

Another microRNA associated with trophoblast proliferation and invasion is miR-18b, which is considered a protective factor against PE, as studies performed in rats showed that high levels of miR-18b contributed to decreased systolic and diastolic blood pressure and proteinuria, parameters that are commonly elevated in PE (81,82). Bai et al. (84) proposed that miR-195 targets genes associated with mechanisms of placental proliferation, apoptosis, and angiogenesis. For this reason, its downregulation contributes to PE pathogenesis by inhibiting cell invasion (58). In addition, miR-149 was reported to be related to cancer metastasis/cell migration (78), as well as in the regulation of endothelial cell function (74).

### Altered microRNAs in placenta *vs* blood

The most relevant microRNAs found to be altered in placenta samples are related to the mechanisms of trophoblast proliferation, migration, invasion, apoptosis, and angiogenesis (miR-210 (2); miR-183 (69); miR-16 (71); miR‐18b (81,82); miR-195 (128)). Another important function includes the control of angiogenesis through regulation of VEGF production in placenta (miR-182* (30,37); miR-20a (89,90); miR-20b (88-90)). Additionally, microRNAs regulating endothelium-dependent vasorelaxation (miR-155 (17)), nutrient transfer (miR-363 (126)), and inflammatory response (miR-181a (125)) were found to possibly contribute to the PE onset.

Similarly, the main mechanisms of the most altered microRNAs in peripheral blood samples were regulation of proliferation, migration, and invasion of trophoblastic cells (miR-16 (71); miR-144 (76); miR-18a (129); miR-518b (52)), angiogenesis (miR-27b-3p (91); miR-210 (2)), endothelium-dependent vasorelaxation via eNOS (miR-155 (17)), and transport of nutrients (miR-363 (126)).

### Altered microRNAs in EOPE *vs* LOPE

Studies that analyzed samples of pregnant women with EOPE demonstrated that the most frequently altered microRNAs in this group are mainly related to the mechanisms of trophoblast proliferation, migration, invasion, apoptosis, and angiogenesis (miR-518b (52); miR-125b (64); miR-16 (71); miR-195 (128); miR-149 (74,78)), as well as eNOS modulation (miR-155 (17)) and amino acids transport and nutrient transfer (miR-363 (126)), which may lead to impaired endothelial cell function and poor placentation.

Meanwhile, the most frequently cited microRNAs in studies that analyzed pregnant women with LOPE are related to the regulation of angiogenesis, mitochondrial metabolism, DNA repair (miR-210 (2)), inflammation (miR-181a (125)), and regulation of blood pressure (miR-1183 (114)). This is in accordance with the literature, which reports that EOPE shows a greater association with impaired trophoblast invasion, placental ischemia, and fetal growth restriction compared to LOPE, whereas in LOPE, maternal factors or susceptibility to vascular damage are more relevant. Under this scenario, those with an extremely susceptible vasculature may become preeclamptic simply by the normal adaptation to pregnancy, while those with a healthy vasculature may become preeclamptic only when faced with a major insult arising from defective placentation (4).

### Altered microRNAs in the first and third trimesters

During the first trimester of pregnancy, the most cited microRNA was miR-125b, which acts by inhibiting trophoblast invasion and angiogenesis, leading to impaired endothelial cell function and poor placentation (64). It is important to point out that most of the studies included in this systematic review were conducted during the third trimester of pregnancy and detected changes in the expression levels of several microRNAs involved in many pathways. The most frequently altered microRNAs in the third trimester are mainly related to the mechanisms of regulation of proliferation, migration, and invasion of trophoblastic cells (miR-16 (71); miR-144 (76); miR-18a (127); miR-18b (81,82)), placental proliferation, apoptosis, and angiogenesis (miR-195 (128)), amino acids transport and nutrient transfer (miR-363 (126)), regulation of endothelial cell function (miR-149 (74,78)), regulation several hypoxia response pathways (miR-210 (2)), and eNOS modulation (miR-155 (17)).

Inadequate vascular remodeling and hypoperfused placenta resulting from superficial cytotrophoblast migration toward the uterine spiral arterioles have been characterized as important events that lead to PE. The placenta becomes ischemic, resulting in the release of factors associated with maternal vascular endothelial dysfunction. Endothelial dysfunction is a common phenotype of PE and is characterized by vasoconstriction and decreased blood flow to organs. Furthermore, an increase in immune cells and inflammatory cytokines is associated with endothelial dysfunction during PE (4).

VEGF and placental growth factor (PlGF) play key roles in placental angiogenesis and are thought to be secreted by trophoblastic cells. VEGF is considered essential for the integrity of maternal endothelial cells. Interestingly, increased and decreased levels of VEGF have been found in the maternal circulation in PE. In addition, there is evidence that innate and adaptive immune processes are involved in the pathogenesis of PE. Compared with normal pregnancy, there is a shift in Th-2 to Th-1 type immunity in PE, and the predominant Th1 immunity is related to the poor placentation, exacerbated inflammatory response, and endothelial dysfunction observed in PE (4,36).

Nitric oxide (NO) is a potent vasodilator that causes relaxation of vascular smooth muscle cells through a cyclic guanosine monophosphate pathway. The nitric oxide/nitric oxide synthase system (NO/NOS) is also known to be altered in PE, as decreased levels of NO and increased levels of arginase (which degrades a precursor molecule in the NOS pathway) have been found in PE. In addition, NO deficiency correlates with the metabolic abnormalities observed in PE, such as hypertension, proteinuria, and platelet dysfunction. Therefore, an intact NOS system is thought to be essential for normal spiral artery remodeling and pregnancy (44).

Several miRNAs contribute to the regulation of the aforementioned processes. As the knowledge of the functional importance of miRNAs in adequate placentation and the development of PE increases, it becomes important to determine which specific miRNAs predominantly contribute to different pathogenic aspects of PE, considering the fact that the subtype of PE is an important clinical variable in predicting both maternal and perinatal outcomes. Although being less frequent, EOPE is associated with higher rates of neonatal mortality, fetal growth restriction, and greater degree of maternal morbidity compared to LOPE, which is also less likely to predict later-life cardiovascular disease (22,54,65). In this sense, patterns of miR expression could help link pathological processes to the different classifications of PE, resolving the ambiguity between EOPE and LOPE cases and making it more likely to achieve a correct diagnosis or choosing an effective and personalized preventive therapy.

In addition, samples from early gestational stages have great potential in revealing miRs that may only be transiently expressed, but that may be biomarkers of increased PE risk. For example, the significantly increased expression of miR-125b in the first trimester suggests that this may be a useful biomarker of increased risk of PE even months before diagnosis. By expanding the question of contribution towards PE classification, certain miRs expression profiles in the first trimester appear to correlate with worst outcomes, as with miR-125b, miR-143, miR-192, miR-320a, miR518b, and miR-518b, since they were increased in EOPE, but not in LOPE. In summary, these findings offer a better understanding of the role of microRNAs in PE and contribute to primary research aimed at finding biomarkers for PE.

## Relevance and limitations

The present study is of great relevance to the academic and clinical fields. Systematic reviews are the type of publication with the highest level of scientific evidence. Therefore, our results will serve to guide future research aimed at determining patterns of microRNA expression in clinical samples from preeclamptic women. In turn, these studies may influence health decision-makers to establish tests aimed at the early prediction of the disease, which results in better prognoses for women who develop PE and their offspring.

Some limitations of this study should be commented. The main one is the heterogeneity of the studies and the lack of some important data. The studies included in this review adopted different diagnostic criteria, as well as different inclusion and exclusion criteria. As a result, the groups of pregnant women included in each study vary widely. Participants had different maternal age, gestational age at which biological samples were collected, and time and mode of delivery. Several studies do not mention clinical characteristics relevant to the context of PE, such as systolic and diastolic pressure, proteinuria levels, platelet count, liver and kidney function markers, among others. Some studies do not even mention the unit of measurement of some of the parameters evaluated.

When placental tissue samples were used for analysis, the region of the placenta from which the biopsy was taken varied greatly between studies, with several studies not even describing the region. In the evaluation of circulating microRNAs, both plasma and serum samples were used. The collection and processing of such samples took place under different protocols or were not clearly described.

Due to the aforementioned reasons, it was not possible to carry out a meta-analysis of the collected data, since there are several confounding factors. Therefore, we reinforce the importance of writing good academic articles, since high quality and accurate information can be used in decision making in clinical practice, helping medicine and society in general.

The identification of pregnant women at increased risk for PE is the most desirable thing for obstetricians. This review listed the most frequently cited upregulated and downregulated microRNAs in PE. It will certainly serve as a reference to guide future experimental research by our research group, as well as other research groups around the world. The greatest knowledge about expression profiles of microRNAs in PE can undoubtedly help in the development of new protocols for early detection of the risk of developing the disease.

## Supplementary Material

Click to view [pdf].
