# Supplementary material for: Changes in microRNA expression associated with preeclampsia: a systematic review
Source: Braz J Med Biol Res. 2025 May 30;58:e13988. doi: 10.1590/1414-431X2025e13988 (PMC12128778; doi:10.1590/1414-431X2025e13988)
Supplement: Supplementary file 1 [file 1414-431X-bjmbr-58-e13988-suppl.pdf]

**Table S1:** Complete search strategy for each database.

| Strategy and Databases | Search Terms |
|---|---|
| **Search strategy** | Pre-Eclampsia<br>OR Pre Eclampsia<br>OR Preeclampsia<br>OR Pregnancy Toxemias<br>OR Pregnancy Toxemia<br>OR Edema Proteinuria Hypertension Gestosis<br>OR Hypertension, Pregnancy-Induced<br>OR hypertension pregnancy<br>OR (high blood pressure AND pregnancy)<br>OR (protein excretion AND pregnancy)<br>OR (proteinuria AND pregnancy)<br>OR HELLP syndrome AND MicroRNAs<br>OR MicroRNA OR miRNAs<br>OR Micro RNA |
| **PubMed** | Pre-Eclampsia (Title/Abstract)<br>OR Pre Eclampsia (Title/Abstract)<br>OR Preeclampsia (Title/Abstract)<br>OR Pregnancy Toxemias (Title/Abstract)<br>OR Pregnancy Toxemia (Title/Abstract)<br>OR Edema Proteinuria Hypertension Gestosis (Title/Abstract)<br>OR Hypertension, Pregnancy-Induced (Title/Abstract)<br>OR hypertension pregnancy (Title/Abstract)<br>OR high blood pressure (Title/Abstract) AND pregnancy (Title/Abstract)<br>OR protein excretion (Title/Abstract) AND pregnancy(Title/Abstract)<br>OR proteinuria (Title/Abstract) AND pregnancy (Title/Abstract)<br>OR toxemia (Title/Abstract)<br>OR HELLP syndrome (Title/Abstract) AND (MicroRNAs (Title/Abstract)<br>OR MicroRNA (Title/Abstract)<br>OR miRNAs (Title/Abstract)<br>OR Micro RNA (Title/Abstract)<br>OR miRNA (Title/Abstract) |
| **EMBASE** | 'pre eclampsia':ab,ti<br>OR preeclampsia:ab,ti<br>OR 'pregnancy toxemias':ab,ti<br>OR 'pregnancy toxemia':ab,ti<br>OR 'edema proteinuria hypertension gestosis':ab,ti<br>OR 'hypertension, pregnancy-induced':ab,ti<br>OR 'hypertension pregnancy':ab,ti<br>OR 'high blood pressure':ab,ti AND pregnancy:ab,ti<br>OR 'protein excretion':ab,ti AND pregnancy:ab,ti<br>OR proteinuria:ab,ti AND pregnancy:ab,ti<br>OR 'hellp syndrome':ab,ti) AND (micrornas:ab,ti<br>OR microrna:ab,ti<br>OR mirnas:ab,ti<br>OR 'micro rna':ab,ti<br>OR mirna:ab,ti |
| **Web of Science** | AB=(Pre-Eclampsia<br>OR Pre Eclampsia<br>OR Preeclampsia<br>OR Pregnancy Toxemias<br>OR Pregnancy Toxemia<br>OR Edema Proteinuria Hypertension Gestosis<br>OR Hypertension, Pregnancy-Induced<br>OR hypertension pregnancy<br>OR high blood pressure AND pregnancy<br>OR protein excretion AND pregnancy)<br>OR (proteinuria AND pregnancy)<br>OR HELLP syndrome) AND AB=(MicroRNAs<br>OR MicroRNA<br>OR miRNAs OR Micro RNA<br>OR miRNA |
| **LILACS** | Title, Abstract, Subject<br>Pre-Eclampsia<br>OR Pre Eclampsia<br>OR Preeclampsia<br>OR Pregnancy Toxemias OR Pregnancy Toxemia |

| | |
|---|---|
| | OR Edema Proteinuria Hypertension Gestosis<br>OR Hypertension, Pregnancy-Induced<br>OR hypertension pregnancy<br>OR high blood pressure AND pregnancy<br>OR protein excretion AND pregnancy<br>OR proteinuria AND pregnancy<br>OR HELLP syndrome AND MicroRNAs<br>OR MicroRNA<br>OR miRNAs<br>OR Micro RNA<br>OR miRNA |

Cochrane and Scielo were not included in Table S1 because no publications were found in these databases that had not already been selected from the other cited databases (PubMed, EMBASE, Web of Science, and LILACS). However, it is worth noting that the terms used for the search were the same for all databases.

**Table S2.** Studies included in the systematic review. See the reference list at the end of the supplementary tables.

| Authors | Reference | Methodological design | Biological sample | Analyzed miRNAs | Upregulated microRNAs | Downregulated microRNAs |
|---|---|---|---|---|---|---|
| Pineles et al 2007 | (175) | Cross-sectional, case-control study | Placenta | 157 miRNAs | miR-182<br>miR-210<br>miR-210<br>miR-155<br>miR-181b<br>miR-182*<br>miR-200b<br>miR-154*<br>miR-183 | NA |
| Hu et al 2009 | (68) | Case control | Placenta | miR-181a<br>miR-195<br>miR-222<br>miR-16<br>miR-29b<br>miR-26b<br>miR-335 | miR-16<br>miR-29b<br>miR-195<br>miR-26b<br>miR-181a<br>miR-335<br>miR-222 | miR-214<br>miR-658<br>miR-532-3p<br>miR-423-5p<br>miR-491-5p<br>miR-612<br>miR-508-5p |
| Zhu et al 2009 | (69) | Case control | Placenta | miR-210<br>miR-152<br>miR-411<br>miR-377<br>miR-518b<br>miR-18a<br>miR-363<br>miR-542-3p | miR-181a<br>miR-584<br>miR-30a-3p<br>miR-210<br>miR-152<br>miR-517*<br>miR-518b<br>miR-519e*<br>miR-638<br>miR-296<br>miR-362 | miR-101<br>miR-10b<br>miR-218<br>miR-590<br>miR-204<br>miR-32 0.503<br>miR-126* 0.522<br>miR-18a 0.437<br>miR-19a 0.488<br>miR-411<br>miR-377<br>miR-154*<br>miR-625<br>miR-144<br>miR-195<br>miR-150<br>miR-1<br>miR-18b<br>miR-363<br>miR-542-3p<br>miR-450<br>miR-223<br>miR-374 0.349 |
| Lazar et al 2010 | (230) | Case control | Placenta | miRNA-325 | NA | miR-325 |
| Zhang et al 2010 | (95) | Case control | Placenta | miR-155 | miR-155 | NA |
| Enquobahrie et al 2011 | (173) | Cohort/case control | Placenta | hsa-miR-518c<br>hsa-miR-1<br>hsa-miR-103<br>hsa-miR-15a<br>hsa-miR-584<br>hsa-miR-324-5p<br>hsa-miR-200b | miR-210 | miR-328<br>miR-584<br>miR-139-5p<br>miR-500<br>miR-1247<br>miR-34c-5p<br>miR-1 |

| | | | | | | |
|---|---|---|---|---|---|---|
| | | | | hsa-miR-210<br>hsa-miR-154 | | |
| Mayor-Lynn K et al 2011 | (174) | Cohort | Placenta | 820 miRNAs<br>miR-15b<br>miR-181a<br>miR-200C<br>miR-210<br>miR-296-3p<br>miR-377<br>miR-483-5p<br>miR-493 | miR-296-3p<br>miR-483-5p<br>miR-377<br>miR-210 | miR-200c |
| Noack et al 2011 | (222) | Case control | Placenta | let-7b<br>miR-302*<br>miR-104<br>miR-128a<br>miR-182*<br>miR-133b | let-7b<br>miR-302*<br>miR-104<br>miR-128a<br>miR-182*<br>miR-133b | NA |
| Bai et al 2012 | (120) | Case control | Placenta | pri-miR-195<br>miR-195 | NA | pri-miR-195<br>miR-195 |
| Ishibashi et al 2012 | (238) | Case control | Placenta | Table 1 | miR-210<br>miR-193b<br>miR-144*<br>miR-193b*<br>miR-18a<br>miR-185<br>miR-19a<br>miR-590–5p<br>miR-142–3p<br>miR-451<br>miR-22*<br>miR-526b*<br>miR-520a-3p<br>miR-10b<br>miR-20a<br>miR-518f*<br>miR-146b-5p<br>miR-517c<br>miR-518c<br>miR-525–5p<br>miR-519e*<br>miR-126* | miR-224 |
| Lázár et al 2012 | (229) | Case control | Placenta | miR-325 | NA | miR-325 |
| Wang et al 2012 | (172) | Case control | Placenta | miR-20b<br>miR-151-3p<br>miR-524-3p<br>miR-34c-5p<br>miR-20a<br>miR-17<br>miR-1975 | miR-20b<br>miR-516a-5p<br>miR-512–3p<br>miR-2277<br>miR-524-3p<br>miR-17<br>miR-20a<br>miR-20b | miR-151-3p<br>miR-146a<br>miR-192<br>miR-34c-5p |
| Wu et al 2012 | (66) | Case control | Blood | miR-574-5p<br>miR-26a<br>miR-151-3p<br>miR-130a<br>miR-181a<br>miR-130b<br>miR-30d<br>miR-145<br>miR-103<br>miR-425<br>miR-221<br>miR-342-3p<br>miR-24<br>miR-144<br>miR-16 | miR-574-5p<br>miR-26a<br>miR-151-3p<br>miR-130a<br>miR-181a<br>miR-130b<br>miR-30d<br>miR-145<br>miR-103<br>miR-425<br>miR-221<br>miR-342-3p<br>miR-24<br>miR-24<br>miR-26a<br>miR-103<br>miR-130b<br>miR-181a<br>miR-342-3p<br>miR-574-5p | miR-144<br>miR-16 |
| Zhang et al 2012 | (67) | Case control | Blood | miR-210 | miR-210 | NA |
| Anton et al 2013 | (168) | A case-control study and a prospective cohort study | Blood | miR-210 | miR-210 | NA |
| Betoni et al 2013 | (169) | Case control | Placenta | 847 miRNAs<br>miR-210 | miR-210_st<br>miR-193b_st | NA |

| | | | | | | |
|---|---|---|---|---|---|---|
| | | | | miR-182*<br>miR-30a-3p | miR-210<br>miR-182* | |
| Choi et al 2013 | (224) | Case control | Placenta | 158 miRNAs<br>miR-92b<br>miR-197<br>miR-342-3p<br>miR-296-4p<br>miR-26b<br>miR-25<br>miR-296-3p | miR-92b<br>miR-197<br>miR-342-3p<br>miR-296-5p<br>miR-26b<br>miR-25<br>miR-296-3p<br>miR-26a<br>miR-198<br>miR-202<br>miR-191<br>miR-95<br>miR-204<br>miR-92b<br>miR-197<br>miR-342-3p<br>miR-296-4p<br>miR-26b<br>miR-25<br>miR-296-3p | miR-21<br>miR-223 |
| Guo et al 2013 | (170) | Case control | Placenta | miR-520A-5p<br>miR-194<br>miR-412<br>miR-483<br>miR-503 | NA | miR-194<br>miR-149 |
| Hromadnikova et al 2013 | (206) | Cohort | Blood | miR-516-5p<br>miR-517*<br>miR-518b<br>miR-520a*<br>miR-520h<br>miR-525<br>miR-526a | miR-516-5<br>miR-517*<br>miR-520a*<br>miR-525<br>miR-526a | NA |
| Kumar et al 2013 | (171) | Case control | Placenta | miR-106a<br>miR-19b | miR-106a<br>miR-19b | NA |
| Li et al 2013 | (65) | Case control | Blood | miR-141<br>miR-144<br>miR-221<br>miR-29a | miR-141<br>miR-29a<br>miR-141<br>miR-221<br>miR-29a | miR-144 |
| Campos et al 2014 | (213) | Case control | Blood | miR-125b<br>miR-146a<br>miR-196b | NA | miR-196b |
| Doridot et al 2014 | (257) | Case control | Placenta | pri-miR-34a | pri-miR-34a | NA |
| Hong et al 2014 | (61) | Case control | Placenta | miR-126 | NA | miR-126 |
| Lalevée et al 2014 | (261) | Prospective case–control | Placenta | miR-526B<br>miR-518B<br>miR-517A<br>miR-210<br>miR-455-3P<br>miR-455-5P | miR-210 | miR-455-3P<br>miR-455-5P |
| Li et al 2014 | (62) | Case control | Placenta | miR-125b-1-3p | miR-125b-1-3p | NA |
| Li et al 2014 | (63) | Case control | Placenta | miR-155 | miR-155 | NA |
| Luo et al 2014 | (94) | Case control | Placenta | miR-210 | miR-210 | NA |
| Luque et al 2014 | (237) | Nested case-control | Blood | 754 miRNAs<br>miR-192<br>miR-143<br>miR-125b<br>miR-127<br>miR-942<br>miR-126#<br>miR-221 | miR-192<br>miR-143<br>miR-125b | miR-127<br>miR-942<br>miR-126#<br>miR-221 |
| Ura et al 2014 | (236) | Retrospective study | Blood | 754 miRNAs<br>miR-1233<br>miR-520<br>miR-210<br>miR-144 | miR-1233<br>miR-650<br>miR-520a<br>miR-215<br>miR-210<br>miR-25<br>miR-518b<br>miR-193a-3p<br>miR-32<br>miR-204 | miR-126<br>miR-335<br>miR-144<br>miR-204<br>miR-668<br>miR-376a<br>miR-15b |

| | | | | | | |
|---|---|---|---|---|---|---|
| | | | | | miR-296-5p<br>miR-152 | |
| Weedon-Fekjær et al 2014 | (259) | Case control | Placenta | ~900 miRNAs<br>miR-223-3p<br>miR-224-5p<br>miR-1301 | 17 miRNAs<br>miR-210 | 12 miRNAs<br>miR-223-3p miR-224-5p<br>miR-1301 |
| Xu et al 2014 | (64) | Prospective cohort | Blood + Placenta | 211 miRNAs<br>miR-17<br>miR-18a<br>miR-19b1<br>miR-92a1<br>miR-210 | miR-181a<br>miR-584<br>miR-30a-3p<br>miR-151<br>miR-31<br>miR-210<br>miR-17-3p<br>miR-193b<br>miR-638<br>miR-525*<br>miR-515-3p<br>miR-519e*<br>miR-518b<br>miR-524<br>miR-296<br>miR-362<br>miR-210<br>miR-30a-3p<br>miR-518b<br>miR-524<br>miR-17-3p<br>miR-151<br>miR-193b<br>miR-210 | miR-214<br>miR-218<br>miR-590<br>miR-18a<br>miR-19a<br>miR-379<br>miR-411<br>miR-195<br>miR-223<br>miR-363<br>miR-542-3p<br>miR-195<br>miR-223<br>miR-218<br>miR-18a<br>miR-379<br>miR-411<br>miR-18a<br>miR-19b1<br>miR-92a1 |
| Zou et al 2014 | (121) | Case control | Placenta | miR-101 | NA | miR-101 |
| Akehurst et al 2015 | (198) | Cohort | Blood + Placenta | 754 miRNAs | miR-196b-5p<br>miR-206-5p<br>miR-502-5p<br>miR-503-5p<br>miR-758-3p | miR-23a* |
| Anton et al 2015 | (167) | Case control | Placenta | miR-517a/b<br>miR-517c | miR-517a/b<br>miR-517c | NA |
| Ding et al 2015 | (59) | Case control | Placenta | miR-519d-3p | miR-519d-3p | NA |
| Hromadnikova et al 2015 | (204) | Retrospective cohort | Placenta | 32 microRNAs miR-1-3p miR-16-5p miR-17-5p miR-20a-5p miR-20b-5p miR-21-5p miR-23a-3p miR-24-3p miR-26a-5p miR-29a-3p miR-33a-5p miR-92a-3p miR-100-5p miR-103a-3p miR-122-5p miR-125b-5p miR-126-3p, miR-130b-3p miR-133a-3p miR-143-3p miR-145-5p miR-146a-5p miR-155-5p miR-181a-5p miR-195-5p miR-199a-5p miR-208a-3p miR-210-3p miR-221-3p miR-342-3p miR-499a-5p miR-574-3p | miR-499a-5p | NA |
| Hromadnikova et al 2015 | (205) | Retrospective cohort | Placenta | miR-512-5p miR-515-5p miR-516-5p miR-517-5p miR-518b miR-518f-5p miR-519a miR-519d miR-519e-5p miR-520a-5p miR-520h miR-524-5p miR-525 | NA | miR-515-5p<br>miR-517-5p<br>miR-518b<br>miR-518f-5p<br>miR-519a<br>miR-519d<br>miR-520a-5p<br>miR-520h<br>miR-524-5p<br>miR-525<br>miR-526a |

| | | | | | | |
|---|---|---|---|---|---|---|
| | | | | miR-526a<br>miR-526b | | |
| Jiang et al 2015 | (93) | Case control | Placenta | miR-335<br>miR-584 | miR-335<br>miR-584 | NA |
| Lasabová et al 2015 | (248) | Cohort | Placenta | miR-155<br>miR-122<br>miR-21 | miR-155<br>miR-122<br>miR-21 | NA |
| Li et al 2015 | (57) | Cohort | Blood | 10 microRNAs | miR-152<br>miR-183<br>miR-210<br>miR-182 | NA |
| Miura et al 2015 | (239) | Case control | Blood | miR-518b<br>miR-1323<br>miR-516b<br>miR-516a-5p<br>miR-525-5p<br>miR-515-5p<br>miR-520 h<br>miR-520a-5p<br>miR-519d<br>miR-526b | miR-518b<br>miR-1323<br>miR-516b<br>miR-516a-5p<br>miR-525-5p<br>miR-515-5p<br>miR-520 h<br>miR-520a-5p<br>miR-519d<br>miR-526b | NA |
| Murphy et al 2015 | (227) | Prospective longitudinal cohort | Blood | let-7f-5p<br>miR-98-5p<br>miR-221-3p miR-222-3p miR-126-3p<br>miR-130a-3p<br>miR-210-3p<br>miR-155-5p<br>miR-17-5p<br>miR-18a-5p miR-19a-3p miR-29a-3p<br>miR-92a-3p miR-20a-5p miR-20b-5p<br>miR-15b-5p miR-16-5p miR-296-3p miR-181a-5p<br>miR-195-5p<br>miR-29b-3p | miR-98-5p<br>miR-222-3p<br>miR-210-3p<br>miR-155-5p<br>miR-296-3p<br>miR-181a-5p<br>miR-29b-3p | miR-16-5p |
| Sun et al 2015 | (58) | Case control | Placenta | miR-34a | miR-34a | NA |
| Zhang et al 2015 | (60) | Case control | Placenta | miR-106a<br>miR-106a<br>miR-18b<br>miR-20b<br>miR-19b-2<br>miR-92a-2<br>miR-363 | miR-1<br>miR-16<br>miR-19b<br>miR-20a<br>miR-125b-1-3p<br>miR-181a<br>miR-182<br>miR-210<br>miR-355<br>miR-424<br>miR-1469<br>miR-106a | miR-29a-3p<br>miR-200c<br>miR-335<br>miR-363<br>miR-584<br>miR-744<br>miR-1826<br>miR-18b<br>miR-19b<br>miR-92a<br>miR-363 |
| Brooks et al 2016 | (178) | Case control | Placenta | Supplemental Table 2 | Supplemental Table 2 | Supplemental Table 2 |
| Hromadnikova et al 2016 | (203) | Retrospective cohort | Blood | miR-1-3p miR-16-5p<br>miR-17-5p<br>miR-20a-5p miR-20b-5p miR-21-5p<br>miR-23a-3p<br>miR-24-3p<br>miR-26a-5p<br>miR-29a-3p miR-92a-3p miR-100-5p<br>miR-103a-3p<br>miR-122-5p miR-125b-5p<br>miR-126-3p<br>miR-130b-3p<br>miR-133a-3p<br>miR-143-3p<br>miR-145-5p miR-146a-5p<br>miR-181a-5p<br>miR-195-5p miR-199a-5p<br>miR-210-3p miR-221-3p miR-342-3p<br>miR-499a-5p | NA | miR-100-5p<br>miR-125b-5p |

| | | | | | | |
|---|---|---|---|---|---|---|
| | | | | miR-574-3p | | |
| Hu et al 2016 | (92) | Case control | Placenta | miR-200c<br>miR-20a<br>miR-20b | miR-200c<br>miR-20a<br>miR-20b | NA |
| Munaut et al 2016 | (246) | Prospective cohort | Blood | hsa-miR-144-3p<br>hsa-miR-29a-3p<br>hsa-miR-210-3p<br>hsa-miR-210-5p<br>hsa-miR-1233-3p<br>hsa-miR-1233-5p<br>hsa-miR-24-3p<br>hsa-miR-26a-5p<br>hsa-miR-130b-3p<br>hsa-miR-130b-5p<br>hsa-miR-181a-5p<br>hsa-miR-181a-3p<br>hsa-miR-342-3p<br>hsa-miR-574-5p<br>hsa-miR-16-2-3p<br>hsa-miR-124-3p<br>hsa-miR-155-5p | miR-210-3p<br>miR-210-5p<br>miR-1233-3p<br>miR-574-5p | NA |
| Ospina-Prieto et al 2016 | (221) | Case control | Placenta | miR-141 | miR-141 | NA |
| Sandrim et al 2016 | (212) | Case control | Blood | 84 miRs<br>miR-885-5p miR-376<br>miR-19a miR-19b<br>miR-423<br>miR-885-5p | miR-885-5p<br>miR-885-5p<br>miR-885-5p | miR-376c-3p<br>miR-19a-3p<br>miR-19b-3p |
| Wang et al 2016 | (56) | Cohort | Blood | miR-451a<br>miR-15a-3P<br>miR-31-3P<br>miR-122-5P | miR-122-5p<br>miR-451a<br>miR-299-5p<br>miR-1299<br>miR-451a | miR-15a-3p<br>miR-31-3p<br>miR-4785<br>miR-4752<br>miR-15a-3p<br>miR-31-3p |
| Yang et al 2016 | (54) | Case control | Blood + Placenta | miR-125b | miR-125b | NA |
| Zhang et al 2016 | (177) | Case control | Placenta | miR-519e-5p<br>miR-515-5p<br>miR-518f<br>miR-519c-3p | NA | miR-519e-5p<br>miR-515-5p<br>miR-518f<br>miR-519c-3p<br>miR-515-3p<br>miR-520d-5p<br>miR-524-5p<br>miR-520a-5p<br>miR-516a-5p<br>miR-518b<br>miR-519e-5p<br>miR-515-5p<br>miR-518f<br>miR-519c-3p |
| Zhou et al 2016 | (55) | Case control | Placenta | 787 miRNAs miR-148a-3p<br>miR-210<br>miR-193b-3p<br>miR-31-5p<br>miR-365a-3p<br>miR-516b-5p<br>miR-520a-5p miR-27a-5p miR-135b-5p miR-136-3p<br>miR-10b-5p miR-192-5p | miR-148a-3p<br>miR-210<br>miR-193b-3p<br>miR-31-5p<br>miR-365a-3p<br>miR-516b-5p<br>miR-520a-5p<br>miR-27a-5p | miR-135b-5p<br>miR-136-3p |
| Adel et al 2017 | (190) | Case control | Placenta | miR-210 | miR-210 | NA |
| Azizi et al 2017 | (209) | Case control | Placenta | miR-155-5p | miR-155-5p | NA |
| Fang et al 2017 | (52) | Case control | Placenta | miRNA-218 | miR-218 | NA |
| Gan et al 2017 | (51) | Case control | Blood | miR-210<br>miR-155<br>miR-125b-5p<br>miR-125a-5p | miR-210<br>miR-155 | miR-125a-5p |
| Gunel et al 2017 | (182) | Case control | Blood + Placenta | 12 miRNAs | let-7b*<br>let-7f-1*<br>miR-1183<br>miR-23c<br>miR-425* | NA |
| Guo et al 2017 | (49) | Case control | Placenta | miR-34a | miR-34a | NA |
| Hromadnikova | (202) | Nested case control | Blood | miR-516b-5p | miR-517-5p | NA |

| | | | | | | |
|---|---|---|---|---|---|---|
| et al 2017 | | study of a longitudinal cohort | | miR-517-5p miR-518b miR-520a-5p miR-520h miR-525-5p | miR-518b miR-520h | |
| Jairajpuri et al 2017 | (244) | Case control | Blood | 84 miRNAs | miR-215 miR-155 miR-650 miR-210 miR-21 | miR-18a miR-19b1 |
| Jiang et al 2017 | (89) | Cohort | Blood | miR-520g | miR-520g | NA |
| Jin et al 2017 | (48) | Case control | Blood + Placenta | miR-20b | miR-20b | NA |
| Korkes et al 2017 | (176) | Case control | Placenta | miR-210 | miR-210 | NA |
| Lu et al 2017 | (91) | Case control | Placenta | miR-137 | miR-137 | NA |
| Luo et al 2017 | (1) | Case control | Placenta | miR-148a | NA | miR-148a |
| Nizyaeva et al 2017 | (197) | Case control | Placenta | miR-146a miR-155 | miR-155 | miR-146a miR-155 |
| Shao et al 2017 | (53) | Case control | Placenta | miR-22 | miR-22 | NA |
| Singh et al 2017 | (166) | Cohort/case control | Placenta | miR-202-3p | miR-202-3p has-miR-202-3p_st ENSG00000221611 _st hsa-miR-25-star_st hsa-miR-451_st has-miR548aj_st | hp_hsa-mir-320b-1_x_st has-miR-432-star_st hsa-miR-4701-3p_st hp_hsa-miR-933_st |
| Tsai et al 2017 | (254) | Case control | Blood + Placenta | miR-346 miR-582-3p | miR-346 miR-582-3p | miR-346 |
| Wang et al 2017 | (47) | Case control | Placenta | miR-18b | NA | miR-18b |
| Xiao et al 2017 | (50) | Case control | Placenta | miR-144 | NA | miR-144 |
| Xu et al 2017 | (90) | Case control | Placenta | let-7i | NA | let-7i |
| Yang et al 2017 | (88) | Case control | Blood + Placenta | miR-155 | miR-155 | NA |
| Zhang et al 2017 | (122) | Nested case-control study | Blood | miR-942 | NA | miR-942 |
| Brkić et al 2018 | (228) | Case control | Placenta | miR-218-5p | NA | miR-218-5p |
| Chen et al 2018 | (38) | Case control | Placenta | miR-210 | miR-210 | NA |
| Chi; Zhang 2018 | (83) | Case control | Placenta | miR-145 | NA | miR-145 |
| Fang et al 2018 | (40) | Case control | Placenta | miR-182-5p | miR-182-5p | NA |
| Fu et al 2018 | (39) | Case control | Placenta | miRNA-517-5p | miR-517-5p | NA |
| Gao et al 2018 | (87) | Case control | Placenta | miR-299 | miR-299 | NA |
| Gunel et al 2018 | (181) | Case control | Blood + Placenta | miR-195 | NA | miR-195 |
| Khaliq et al 2018 | (255) | Case control | Blood + Placenta | miR-222 miR-29a miR-181a | miR-29a miR-181a | miR-222 |
| Kim et al 2018 | (223) | Case control | Blood | miR-31-5p | miR-31-5p | NA |
| Li et al 2018 | (43) | Case control | Blood + Placenta | miRNA-376c | NA | miR-376c |
| Liu et al 2018 | (45) | Case control | Placenta | miR-203 | miR-203 | NA |
| Lykoudi et al 2018 | (250) | Case control | Placenta | miR-518a-5p | PE miR-124* miR-130b miR-155 miR-383 miR-423-3p miR-431 miR-500a miR-1183 miR-1305 miR-124* miR-130b miR-383 miR-423-3p miR-431 miR-518a-5p miR-383 miR-1183 | miR-126* miR-544 miR-3942 miR-544 miR-3942 |
| Martinez-Fierro et al 2018 | (234) | Nested cohort case–control | Blood | 51 members of chromosome 19 miRNA cluster | miR-520c-3p miR-518f miR-512-3p miR-520d-3p | NA |
| Niu et al 2018 | (46) | Case control | Placenta | miR-30a-3p | miR-30a-3p | NA |
| Nizyaeva et al 2018 | (200) | Case control | Placenta | miR-17 miR-181a miR-519a | miR-519a miR-181a | miR-181a |
| Sandrim et al 2018 | (214) | Case control | Blood | Let-7a-5p | NA | Let-7a-5p |

| | | | | | | |
|---|---|---|---|---|---|---|
| Timofeeva et al 2018 | (196) | Cohort 1 | Blood + Placenta | hsa-miR-532-5p<br>hsa-miR-423-5p<br>hsa-miR-127-3p<br>hsa-miR-539-5p<br>hsa-let-7c-5p<br>hsa-miR-629-5p<br>hsa-miR-519a-3p | miR-423-5p<br>miR-629-5p<br>miR-519a-3p<br>let-7c-5p<br>miR-519a-3p | miR-532-5p<br>miR-423-5p<br>miR-127-3p<br>miR-539-5p<br>miR-629-5p<br>miR-519a-3p<br>let-7c-5p<br>miR-532-5p<br>miR-127-3p<br>miR-539-5p<br>miR-629-5p<br>miR-519a-3p<br>let-7c-5p |
| Wang et al 2018 | (242) | Case control | Placenta | miR-181a-5p<br>miR-181a-3p<br>miR-181c<br>let-7<br>miR-625<br>miR-454<br>miR-34c<br>miR-483-3p<br>miR-663b | miR-892c-3p<br>miR-378c<br>miR-514b-3p<br>miR-663 | NA |
| Wang et al 2018 | (123) | Cohort/case control | Blood | miR-195 | NA | miR-195<br>miR-195 |
| Wang; Yan 2018 | (44) | Case control | Placenta | miR-454 | NA | miR-454 |
| Wu et al 2018 | (86) | Case control | Placenta | miR-181a-5p | miR-181a-5p | NA |
| Awamleh et al 2019 | (226) | Cohort | Placenta | hsa-miR-193b-5p<br>hsa-miR-193b-3p<br>hsa-miR-210-3p<br>hsa-miR-365a-3p<br>hsa-miR-365b-3p<br>hsa-miR-520a-3p<br>hsa-miR-210-5p<br>hsa-miR-181a-2-3p<br>hsa-miR-33b-3p | miR-210-5p<br>miR-181a-2-3p<br>miR-33b-3p | NA |
| Biró et al 2019 | (231) | Case control | Placenta | miR-210<br>miR-517<br>miR-16 | miR-210<br>miR-517 | NA |
| Cao et al 2019 | (80) | Case control | Blood + Placenta | miR-200a-3p<br>miR-141-3p | miR-200a-3p<br>miR-141-3p | NA |
| Demirer et al 2019 | (179) | Case control | Blood | miR-518b | miR-518b | NA |
| Dong et al 2019 | (30) | Case control | Blood | miR-31<br>miR-21<br>miR-16 | NA | miR-31<br>miR-21 |
| Hocaoglu et al 2019 | (184) | Case control | Blood | miR-21-3p<br>miR-155-5p<br>miR-16-5p | NA | miR-21-3p<br>miR-155-5p |
| Huang et al 2019 | (41) | Case control | Placenta | miR-181a-5p | miR-181a-5p | NA |
| Liu et al 2019 | (36) | Case-control | Placenta | miR-142-3p | miR-142-3p | NA |
| Lv et al 2019 | (28) | Case control | Placenta | miR-145-5p | NA | miR-145-5p |
| Ma et al 2019 | (124) | Prospective cohort study | Blood | miR-133a<br>miR-206 | NA | miR-133a<br>miR-206 |
| Martinez-Fierro 2019 | (233) | Retrospective nested cohort case-control | Blood | hsa-miR-628-3p<br>hsa-miR-628-5p | miR-628-3p<br>miR-628-5p | NA |
| Nejad et al 2019 | (208) | Case control | Blood | miR-210-3p<br>miR-517c-3p | miR-210-3p<br>miR-517c-3p | NA |
| Qian; Liu 2019 | (79) | Case control | Placenta | miR-30b | miR-30b | NA |
| Sekar et al 2019 | (216) | Case control | Blood | miR-510 | miR-510 | NA |
| Shi et al 2019 | (37) | Case-control | Placenta | miR-652-3p<br>miR-210 | miR-210 | miR-652-3p |
| Shi et al 2019 | (84) | Case control | Placenta | miR-454 | NA | miR-454 |
| Tang et al 2019 | (31) | Case control | Placenta | miR-424 | NA | miR-424 |
| Wang et al 2019 | (29) | Case control | Placenta | miR-210 | miR-210 | NA |
| Wang et al 2019 | (34) | Case control | Placenta | miR-210 | miR-210 | NA |
| Wang et al 2019 | (253) | Case-control | Blood | miR-141<br>miR-200a<br>miR-200b<br>miR-429 | miR-141<br>miR-200a | NA |
| Wang et al 2019 | (42) | Case control | Placenta | miR-19a-3p | NA | miR-19a-3p |
| Wang et al | (125) | Case control | Placenta | miR-141-5p | NA | miR-141-5p |

| | | | | | | |
|---|---|---|---|---|---|---|
| 2019 | | | | | | |
| Xie et al 2019 | (32) | Case control | Placenta | miR-320a | NA | miR-320a |
| Xue et al 2019 | (82) | Case control | Placenta | miR-34a-5p | miR-34a-5p | NA |
| Yang et al 2019 | (35) | Case-control | Blood +<br>Placenta | miR-376c<br>miR-377<br>miR-411 | miR-376c<br>miR-441 | NA |
| Yang et al 2019 | (126) | Case control | Placenta | miR-221-3p | NA | miR-221-3p |
| Yang et al 2019 | (127) | Case control | Placenta | miR-26a-5p | miR-26a-5p | NA |
| Yang; Guo<br>2019 | (81) | Case control | Placenta | miR-342-3p | miR-342-3p | NA |
| Yang; Meng<br>2019 | (85) | Case control | Placenta | miR-431 | miR-431 | NA |
| Youssef; Marei<br>2019 | (189) | Case control | Blood | microRNA-210<br>microRNA-155 | miR-210<br>miR-155 | NA |
| Zhong et al<br>2019 | (33) | Cohort/case control | Blood | Table 2 | miR-1304-5p<br>miR-320a<br>miR-5002-5p | 26 miRNAs<br>miR-188-3p<br>miR-211-5p<br>hiv1-miR-TAR-3p<br>miR-4432<br>miR-4498 |
| Demirer et al<br>2020 | (183) | Prospective cohort | Blood | miR-518b<br>miR-155-5p<br>miR-21-3p | miR-518b | NA |
| Dong et al 2020 | (12) | Case control | Placenta | miR-222-5p | NA | miR-222-5p |
| Fan et al 2020 | (21) | Case control | Placenta | miR-23a | miR-23a | NA |
| Gai et al 2020 | (10) | Case control | Placenta | miR-182-5p | miR-182-5p | NA |
| Ghafari et al<br>2020 | (207) | Case control | Blood | miR-155<br>miR-210<br>miR-494<br>miR-29b<br>miR-34a | miR-155<br>miR-210<br>miR-494 | miR-29b |
| Gusar et al<br>2020 | (199) | Case control | Blood +<br>Placenta | miR-27b-3p<br>miR-92b-3p<br>miR-181a-5p<br>miR-186-5p | miR-27b-3p<br>miR-92b-3p<br>miR-181a-5p<br>miR-186-5p | miR-27b-3p<br>miR-186-5p |
| Huang et al<br>2020 | (128) | Case control | Placenta | miR-139-5p | NA | miR-139-5p |
| Jelena et al<br>2020 | (260) | Case control | Blood | miR-210-3p<br>miR-518b | miR-518b | NA |
| Kim et al<br>2020 | (223) | Case control | Blood | miR-31<br>miR-155 | miR-31<br>miR-155 | NA |
| Kim et al 2020 | (223) | Case control | Blood | miR-31-5p | miR-31-5p | NA |
| Kim et al 2020 | (262) | Case control | Blood | miR-31-5p<br>miR-155-5p<br>miR-214-3p<br>miR-1290-3p | miR-31-5p<br>miR-155-5p<br>miR-214-3p | miR-1290-3p |
| Leseva et al<br>2020 | (165) | Cohort/case control | Placenta | miR-138 | miR-138 | NA |
| Li et al 2020 | (25) | Case–control | Blood +<br>Placenta | 16 miRNAs | let-7a-5p<br>miR-15a-5p<br>miR-92a-1-3p<br>miR-106a<br>miR-125b<br>miR-130a-3p<br>miR-191-5p<br>miR-374a-5p<br>miR-574-5p | miR-22–5p<br>miR-93-5p<br>miR-126-3p<br>miR-204-3p<br>miR-365a-3p<br>miR-559-5p<br>miR-4264-5p |
| Li et al 2020 | (129) | Case control | Placenta | miR-383<br>miR-16 | miR-16 | miR-383 |
| Li et al 2020 | (130) | Case control | Placenta | miR-507 | miR-507 | NA |
| Lip et al 2020 | (258) | Case control | Blood | miR-574-5p<br>miR-1972<br>miR-4793-3p | miR-574-5p<br>miR-1972<br>miR-4793-3p | NA |
| Liu et al 2020 | (13) | Case control | Placenta | miR-491-5p | miR-491-5p | NA |
| Liu et al 2020 | (78) | Case control | Placenta | miR-149-5p | NA | miR-149-5p |
| Mavreli et al<br>2020 | (249) | Mix: case<br>control/cohort | Blood | miR-23b-5p<br>miR-99b-5p | NA | miR-23b-5p<br>miR-99b-5p |
| Mi et al 2020 | (76) | Case control | Placenta | miR-196a-5p | NA | miR-196a-5p |
| Sheng et al<br>2020 | (24) | Case control | Blood | miR-206 | miR-206 | NA |
| Suo et al 2020 | (26) | Case control | Placenta | miR-183 | miR-183 | NA |
| Tao et al 2020 | (18) | Case control | Placenta | miR-124-3p | miR-124-3p | NA |
| Whigham et al<br>2020 | (241) | Mix: case<br>control/cohort | Blood +<br>Placenta | 41 miRs<br>miR363<br>miR149 | NA | miR-363<br>miR-149<br>miR-18a |

| | | | | | | |
|---|---|---|---|---|---|---|
| | | | | miR18a<br>miR1283<br>miR16<br>miR424 | | miR-1283<br>miR-16<br>miR-424<br>miR-363<br>miR-18a<br>miR-363<br>miR-149<br>miR-16<br>miR-363<br>miR-149 |
| Wu et al 2020 | (131) | Case control | Placenta | miR-135a-5p | NA | miR-135a-5p |
| Xu et al 2020 | (133) | Case control | Placenta | miR-134-5p | NA | miR-134-5p |
| Yang; Meng 2020 | (27) | Case control | Placenta | miR-215-5p | miR-215-5p | NA |
| Zhang et al 2020 | (14) | Case control | Placenta | hsa-miR-937<br>hsa-miR-148b*<br>hsa-miR-3907<br>hsa-miR-367* | miR-367* | miR-937<br>miR-148b*<br>miR-3907 |
| Zhang et al 2020 | (77) | Case control | Placenta | miR-346 | miR-346 | NA |
| Zheng et al 2020 | (23) | Case control | Blood + Placenta | miR-27a | miR-27a | NA |
| Abbas et al 2021 | (188) | Case control | Placenta | miRNA-452 | miR-452 | NA |
| Akgör et al 2021 | (180) | Case control | Blood | miR-142-3p<br>miR-218-5p<br>miR-125b-5p<br>miR-152<br>miR-17-5p<br>miR-128<br>miR-302b-3p<br>miR-24-3p<br>miR-191-5p<br>miR-328<br>miR-29a-3p<br>miR-132-3p<br>miR-197-3p<br>miR-342-3p<br>miR-375<br>miR-210<br>miR-486-5p<br>miR-139-5p<br>miR-214-3p | miR-210<br>miR-375<br>miR-197-3p<br>miR-132-3p<br>miR-29a-3p<br>miR-328<br>miR-24-3p<br>miR-218-5p | miR-302b-3p<br>miR-191-5p<br>miR-17-5p |
| Ali et al 2021 | (251) | Observational cross-sectional comparative study | Blood | miR-16 | miR-16 | NA |
| Amin-Beidokhti et al 2021 | (210) | Case control | Placenta | miR-517a/b | NA | miR-517a/b |
| Cai et al 2021 | (100) | Cohort | Placenta | miR-519d | NA | miR-519d |
| Chen et al 2021 | (74) | Case control | Placenta | miR-574 | miR-574 | NA |
| Chu et al 2021 | (72) | Case control | Placenta | miR-126-3p | NA | miR-126-3p |
| Fan et al 2021 | (71) | Case control | Placenta | miR-762 | NA | miR-762 |
| Gao et al 2021 | (2) | Cohort | Blood + Placenta | miR-300 | miR-300 | NA |
| Han et al 2021 | (19) | Case control | Blood + Placenta | miR-483 | NA | miR-483 |
| Hu et al 2021 | (6) | Case control | Placenta | miR-149-5p | NA | miR-149-5p |
| Jairajpuri et al 2021 | (243) | Case control | Blood | miRNA-210 | miR-210 | NA |
| Jin et al 2021 | (70) | Case control | Blood | miR-19a<br>miR-126<br>miRNA-210 | miR-19a<br>miR-210 | miR-126 |
| Jin et al 2021 | (105) | Case control | Blood + Placenta | miR-3935 | NA | miR-3935 |
| Kolkova et al 2021 | (247) | Case control | Blood | miR-21-5p<br>miR-155-5p<br>miR-210-5p<br>miR-16-5p<br>miR-650 | miR-21-5p<br>miR-155-5p | NA |
| Lai; Yu 2021 | (17) | Case control | Placenta | miR-183 | miR-183 | NA |
| Lei et al 2021 | (97) | Case control | Placenta | miR-520a-3p | NA | miR-520a-3p |
| Liao et al 2021 | (4) | Case control | Blood | miR-320a | miR-320a | NA |
| Licini et al 2021 | (235) | Case-control study nested in a cohort study | Blood + Placenta | miR-125b | miR-125b | NA |

| | | | | | | |
|---|---|---|---|---|---|---|
| Liu et al 2021 | (8) | Case control | Placenta | microRNA-126 | miR-126 | NA |
| Liu et al 2021 | (114) | Case control | Placenta | miR-558 | NA | miR-558 |
| Luizon et al 2021 | (215) | A case-control study based in a prospective cohort | Blood | 84 circulating miRNAs | miR-204-5p | NA |
| Luo et al | (98) | Case control | Placenta | miR23a-5p | miR23a-5p | NA |
| Mao; Zou 2021 | (104) | Case control | Placenta | miR-326 | miR-326 | NA |
| Martinez-Fierro ML; Garza-Veloz I 2021 | (232) | Retrospective nested cohort case-control | Blood | 768 miRNAs | Supplementary table | miR-133a-3p<br>miR-197-3p |
| Ni et al 2021 | (75) | Case control | Placenta | miR-95-5p | miR-95-5p | NA |
| Sharma et al 2021 | (217) | Case control | Blood + Placenta | miR-146-5p<br>miR-187-5p | miR-146-5p<br>miR-187-5p | miR-146-5p<br>miR-187-5p |
| Song et al 2021 | (16) | Case control | Placenta | miR-655-3p | miR-655-3p | NA |
| Tang et al 2021 | (22) | Case control | Blood | miR-125b | miR-125b | NA |
| Trongpisutsak; Phupong 2021 | (263) | Prospective observational study (cohort) | Blood | miR-210 | NA | miR-210 |
| Wang et al 2021 | (20) | Case control and cohort | Blood + Placenta | miR-155 | miR-155 | NA |
| Witvrouwen et al 2021 | (245) | Prospective case–control | Blood | miR-16<br>miR-29b<br>miR-126<br>miR-155<br>miR-200c | miR-200c | miR-16 |
| Wu et al 2021 | (103) | Case control | Placenta | miR-302a | miR-302a | NA |
| Yang 2021 | (102) | Case control | Placenta | miR-133b | miR-133b | NA |
| Yang et al 2021 | (113) | Case control | Placenta | miR-18b | NA | miR-18b |
| Yin et al 2021 | (9) | Case control | Placenta | miR-138-5p | NA | miR-138-5p |
| Yu et al 2021 | (73) | Case control | Placenta | miR-146a | miR-146a | NA |
| Yu et al 2021 | (132) | Case control | Placenta | miR-218-5p | miR-218-5p | NA |
| Zhang et al 2021 | (11) | Case control | Placenta | miR-525-5p | NA | miR-525-5p |
| Zhao et al 2021 | (3) | Case control | Placenta | miR-135 | NA | miR-135 |
| Zhao et al 2021 | (5) | Case control | Placenta | miR-16 | miR-16 | NA |
| Zhou et al 2021 | (99) | Case control | Placenta | miR-129-2-3p, miR-409-3p, miR-765, miR-371a-5p, miR-296-5p, miR-874-3p, miR-605, miR-513a-5p, miR-513c-5p, miR-126-3p, miR-143-3p, miR-145-5p, miR-193b-5p, miR-27a-5p, miR-412-5p, and miR-497-5p | miR-371a-5p<br>miR-513c-5p<br>miR-126-3p<br>miR-145-5p<br>miR-193b-5p<br>miR-296-5p | NA |
| Zhu; Liu 2021 | (7) | Case control | Blood | miR-27b-3p | miR-27b-3p | NA |
| Zhu; Wang 2021 | (15) | Case control | Placenta | miR-183 | miR-183 | NA |
| Chen et al 2022 | (135) | Case control | Placenta | miR-4443 | miR-4443 | NA |
| Dong et al 2022 | (106) | Cohort | Blood | miR-345–3p | NA | miR-345-3p |
| Du et al 2022 | (96) | Case control | Blood | miR-193b-5p | miR-193b-5p | NA |
| Gu et al 2022 | (137) | Case control | Placenta | miR-30a | miR-20a | NA |
| Hu et al 2022 | (138) | Case control | Placenta | miR-21 | miR-21 | NA |
| Jiang et al 2022 | (117) | Case control | Placenta | miR-140-5p | NA | miR-140-5p |
| Li et al 2022 | (107) | Case control | Placenta | miR-558 | NA | miR-558 |
| Li et al 2022 | (108) | Case control | Blood + Placenta | miR-24-3p | miR-24-3p | NA |
| Li et al 2022 | (140) | Case control | Blood | miR-19a/b-3p | NA | miR-19a/b-3p |
| Li et al 2022 | (141) | Case control | Placenta | miR-24-3p | miR-24-3p | NA |
| Li et al 2022 | (142) | Case control | Placenta | miR-372-3p | miR-372-3p | NA |
| Li; Li 2022 | (118) | Case control | Placenta | miR-424-5p | NA | miR-424-5p |
| Lin et al 2022 | (144) | Case control | Placenta | miR-574-5p | miR-574-5p | NA |
| Liu et al 2022 | (112) | Case control | Placenta | miR-942-5p | NA | miR-942-5p |
| Liu; Wang 2022 | (146) | Case control | Placenta | miR-200b-3p | miR-200b-3p | NA |
| Ning et al 2022 | (149) | Case control | Blood | miR-146b-5p | miR-146b-5p | NA |
| Shang et al 2022 | (151) | Case control | Placenta | miR-558 | NA | miR-558 |
| Simsek et al 2022 | (185) | Case control | Blood + Placenta | miR-1<br>miR-125b | miR-125b | miR-1<br>miR-125b |

| | | | | | | |
|---|---|---|---|---|---|---|
| Wang et al 2022 | (109) | Case control | Placenta | miR-330-5p | miR-330-5p | NA |
| Wang et al 2022 | (116) | Case control | Placenta | miR-346 | miR-346 | NA |
| Wei et al 2022 | (119) | Case control | Placenta | miR-128-3p | miR-128-3p | NA |
| Wei et al 2022 | (157) | Case control | Placenta | miR-24-3p | miR-24-3p | NA |
| Xing et al 2022 | (111) | Case control | Placenta | miR-558 | NA | miR-558 |
| Xu; Teng 2022 | (159) | Case control | Placenta | miR-136 | miR-136 | NA |
| Yang et al 2022 | (101) | Case control | Blood | miR-27b-3p | miR-27b-3p | NA |
| Yousefzadeh et al 2022 | (211) | Case control | Placenta | miR-18a<br>miR-30a<br>miR-206 | miR-30a<br>miR-206 | miR-18a |
| Yuan et al 2022 | (115) | Cohort | Placenta | miR-532-3p<br>miR-423-5p | NA | miR-532-3p<br>miR-423-5p |
| Zhang; Wang 2022 | (110) | Case control | Placenta | miR-202-5p | miR-202-5p | NA |
| Zhou et al 2022 | (160) | Case control | Blood | miR-25-3p | miR-25-3p | NA |
| Zhou et al 2022 | (161) | Case control | Blood | miR-195-5p | NA | miR-195-5p |
| Ali et al 2023 | (252) | Case control | Blood | miR-182-3-p<br>miR-519-d-5p<br>miR-378-3p | miR-182-3-p<br>miR-519-d-5p | miR-378-3p |
| Arora et al 2023 | (218) | Case control | Placenta | miR-22<br>miR-22-3p | miR-22<br>miR-22-3p | NA |
| Artemieva et al 2023 | (201) | Case control | Placenta | miR-146a<br>miR-155 | miR-155 | miR-146a<br>miR-155 |
| Ayoub et al 2023 | (192) | Case control | Blood | miR-186<br>miR-181a | miR-186<br>miR-181a | NA |
| Cao et al 2023 | (134) | Case control | Placenta | miR-19b-3p | NA | miR-19b-3p |
| Chen et al 2023 | (136) | Case control | Placenta | miR-195 | NA | miR-195 |
| Hocaoglu et al 2023 | (186) | Case control | Blood | miR-155-5p<br>miR-518b | miR-155-5p<br>miR-518b | NA |
| Mamdouh et al 2023 | (194) | Case control | Blood | miR-155 | miR-155 | NA |
| Mo et al 2023 | (148) | Case control | Placenta | miR-206 | miR-206 | NA |
| Nunode et al 2023 | (240) | Case control | Blood | miR-515-5p | miR-515-5p | NA |
| Paremmal et al 2023 | (219) | Case control | Placenta | miR-210 | miR-210 | NA |
| Peng et al 2023 | (150) | Case control | Placenta | miR-518a-5p | miR-518a-5p | NA |
| Soobryan et al 2023 | (256) | Case control | Blood | miR-126<br>miR-210<br>miR-29B | miR-126 | NA |
| Su et al 2023 | (152) | Case control | Placenta | miR-494 | miR-494 | NA |
| Sui et al 2023 | (153) | Case control | Placenta | miR-378a-3p | NA | miR-378a-3p |
| Vijayan et al 2023 | (220) | Case control | Placenta | miR-4743-5p<br>miR-331-5p<br>miR-149-5p | miRNA-4743-5p<br>miR-331-5p | miR149-5p |
| Wang et al 2023 | (155) | Case control | Placenta | miR-155 | miR-155 | NA |
| Xu et al 2023 | (158) | Case control | Placenta | miR-101-5p | miR-101-5p | NA |
| Zhou et al 2023 | (21) | Case control | Placenta | miR-188-3p | NA | miR-188-3p |
| Zhu et al 2023 | (163) | Case control | Placenta and Blood | miR-296 | miR-296 | NA |
| Zhu; Chen 2023 | (164) | Case control | Placenta | miR-424 | NA | miR-424 |
| Abbas et al 2024 | (191) | Case control | Blood | miRNA-21 | NA | miRNA-21 |
| Ellakwa et al 2024 | (193) | Case control | Blood | miR-149 | NA | miR-149 |
| Jiang et al 2024 | (139) | Case control | Placenta | miR-217 | NA | miR-217 |
| Liao et al 2024 | (143) | Case control | Placenta | miR-942-5p<br>miR-5006-3p | NA | miR-942-5p<br>miR-5006-3p |
| Liu et al 2024 | (145) | Case control | Placenta | miR-223-3p | NA | miR-223-3p |
| Lu et al 2024 | (147) | Case control | Placenta | miR-508-3p | NA | miR-508-3p |
| Ozler et al 2024 | (187) | Case control | Blood | miR-17<br>miR-19b<br>miR-20a<br>mi-20b<br>miR-126<br>miR-155<br>miR-200<br>miR-210<br>miR-222 | miR-17<br>miR-20a<br>miR-20b<br>miR126<br>miR-210 | NA |
| Senousy et al 2024 | (195) | Prospective study | Blood | miR-29b | miR-29b | NA |
| Tian et al 2024 | (154) | Case control | Placenta | miR-942-5p | NA | miR-942-5p |

| | | | | | | |
|---|---|---|---|---|---|---|
| Wang et al 2024 | (156) | Case control | Placenta | miR-135a | NA | miR-135a |

**Table S3.** List of countries of publications. See the reference list at the end of the supplementary tables.

| Country | Number of studies |
|---|---|
| China | 164 (1–164) |
| USA | 15 (31,165–178) |
| Turkey | 9 (179–187) |
| Egypt | 8 (188–195) |
| Russia | 6 (196–201) |
| Czech Republic | 5 (202–206) |
| Iran | 5 (207–211) |
| Brazil | 5 (176,212–215) |
| India | 5 (216–220) |
| Germany | 3 (165,221,222) |
| South Korea | 3 (223–225) |
| Canada | 3 (226–228) |
| Hungary | 3 (229–231) |
| Mexico | 3 (232–234) |
| Italy | 3 (235–237) |
| Japan | 3 (238–240) |
| Australia | 2 (241,242) |
| Bahrein | 2 (243,244) |
| Belgium | 2 (245,246) |
| Slovakia | 2 (247,248) |
| Greece | 2 (249,250) |
| Pakistan | 2 (251,252) |
| Taiwan | 2 (253,254) |
| South Africa | 2 (255,256) |
| Spain | 1 (237) |
| France | 1 (257) |
| The Netherlands | 1 (258) |
| Norway | 1 (259) |
| Serbia | 1 (260) |
| Switzerland | 1 (261) |
| Korea | 1 (262) |
| Thailand | 1 (263) |

**Table S4.** Upregulated microRNAs related to preeclampsia. See the reference list at the end of the supplementary tables.

| microRNA | General | Early | Late | Placenta | Blood | 1st trimester | 2nd trimester | 3rd trimester |
|---|---|---|---|---|---|---|---|---|
| ENSG00000221611_st | 1 (166) | | | 1 (166) | | | | |
| let-7a-5p | 1 (25) | | | | | 1 (64) | | |
| let-7b | 1 (222) | | | 1 (222) | | | | 1 (222) |
| let-7b* | 1 (182) | | 1 (182) | | | | | 1 (182) |
| let-7c-5p | 1 (196) | 1 (196) | | | 1 (196) | | | 1 (196) |
| let-7f-1* | 1 (182) | | 1 (182) | | | | | 1 (182) |
| miR-1 | 1 (60) | | | 1 (60) | | | | 1 (60) |
| miR-7f | 1 (68) | | 1 (68) | 1 (68) | | | | |
| miR-10b | 1 (238) | | | 1 (238) | | | | |
| miR-10b-3p | 1 (232) | | | | 1 (232) | | | |
| miR-15a-5p | 1 (25) | | | | | 1 (25) | | |
| miR-16 | 5 (5,60,68,129,251) | | 1 (68) | 4 (5,60,68,129) | 1 (251) | | | 5 (5,60,68,129,251) |
| miR-16-5p | 1 (247) | | | | 1 (247) | | | |
| miR-17 | 2 (172,187) | | | 1 (172) | 1 (187) | | | 2 (172,187) |
| miR-17-3p | 1 (64) | | | 1 (64) | | | | |
| miR-18a | 1 (238) | | | 1 (238) | | | | |
| miR-18b-5p | 1 (232) | | | | 1 (232) | | | |
| miR-19a | 2 (70,238) | | | 1 (70) | 1 (238) | | | |
| miR-19b | 2 (60,171) | | | 2 (60,171) | | | | 1 (60) |
| miR-19b-1-5p | 1 (232) | | | | 1 (232) | | | |
| miR-19b-3p | 1 (232) | | | | 1 (232) | | | |
| miR-20a | 5 (60,92,172,187,238) | | | 4 (60,92,172,238) | 1 (187) | | | 4 (60,92,172,187) |
| miR-20b | 5 (48,60,68,172,187) | | 1 (68) | 5 (48,60,68,172,187) | 2 (48,187) | | | 3 (92,172,187) |
| miR-21 | 3 (138,244,248) | | | 2 (138,244) | 1 (244) | | | 2 (138,248) |
| miR-21-5p | 2 (232,247) | | | | 2 (232,247) | | | 1 (247) |
| miR-22 | 2 (53,218) | 2 (53,218) | | 2 (53,218) | | | | 2 (53,218) |
| miR-22* | 1 (238) | | | 1 (238) | | | | |
| miR-22-3p | 1 (218) | 1 (218) | | 1 (218) | | | | 1 (218) |
| miR-23a | 1 (21) | | | 1 (21) | | | | 1 (21) |
| miR-23a-5p | 1 (98) | | | 1 (98) | | | | |
| miR-23c | 1 (182) | | 1 (182) | | | | | 1 (182) |
| miR-24 | 1 (66) | | 1 (66) | | 1 (66) | | | 1 (66) |
| miR-24-3p | 4 (108,141,157,180) | | | 3 (108,141,157) | 1 (180) | | | 3 (108,141,180) |
| miR-25 | 2 (224,236) | | | 1 (224) | 1 (236) | | | 1 (224) |
| miR-25-3p | 1 (160) | | | | 1 (160) | | | 1 (160) |
| miR-25-star_st | 1 (166) | | | 1 (166) | | | | |
| miR-26a | 2 (66,224) | | 1 (66) | 1 (224) | 1 (66) | | | 2 (66,224) |
| miR-26a-5p | 1 (127) | | | 1 (127) | | | | 1 (127) |
| miR-26b | 2 (68,224) | | 1 (68) | 2 (68,224) | | | | 2 (68,224) |
| miR-26b-3p | 1 (232) | | | | 1 (232) | | | |
| miR-27a | 1 (23) | | 1 (23) | 1 (23) | | | | 1 (23) |
| miR-27a-5p | 1 (55) | | | 1 (55) | | | | 1 (55) |
| miR-27b-3p | 4 (7,101,199,232) | 1 (199) | | | 4 (7,101,199,232) | | | 2 (101,199) |
| miR-28-3p | 1 (232) | | | | 1 (232) | | | |
| miR-28-5p | 1 (232) | | | | 1 (232) | | | |
| miR-29a | 2 (65,255) | | | | 2 (65,255) | | | 2 (65,255) |

| | | | | | | | |
|---|---|---|---|---|---|---|---|
| miR-29a-3p | 1 (180) | | | | 1 (180) | | 1 (180) |
| miR-29b | 2 (68,195) | 1 (195) | 2 (68,195) | 1 (68) | 1 (195) | | 2 (68,195) |
| miR-29b-3p | 1 (227) | | | | 1 (227) | | 1 (227) |
| miR- 30a | 2 (137,211) | | | 2 (137,211) | | | 2 (137,211) |
| miR-30a-3p | 3 (46,64,69) | | | 3 (46,64,69) | | | 2 (46,69) |
| miR-30b | 1 (79) | | | 1 (79) | | | 1 (79) |
| miR-30d | 1 (66) | | 1 (66) | | 1 (66) | | |
| miR-30e | 1 (68) | | 1 (68) | 1 (68) | | | |
| miR-30e-3p | 1 (232) | | | | 1 (232) | | |
| miR-31 | 2 (64,223) | | | 1 (64) | 1 (223) | | |
| miR-31-5p | 3 (55,225,262) | | | 1 (262) | 2 (55,64) | | 1 (55) |
| miR-32 | 1 (236) | | | | 1 (236) | | |
| miR-33b-3p | 1 (226) | 1 (226) | | 1 (226) | | | 1 (226) |
| pri-miR-34a | 1(257) | | | 1 (257) | | | |
| miR-34a | 2 (49,58) | | | 2 (49,58) | | | 2 (49,58) |
| miR-34a-3p | 1 (232) | | | | 1 (232) | | 1 (232) |
| miR-34a-5p | 1 (37) | | | 1 (222) | | | |
| miR-92a-1-3p | 1 (25) | | | | | 1 (25) | |
| miR-92b | 1 (224) | | | 1 (224) | | | 1 (224) |
| miR-92b-3p | 1 (199) | 1 (199) | | | 1 (199) | | |
| miR-95 | 1 (224) | | | 1 (224) | | | 1 (224) |
| miR-95-5p | 1 (75) | | | 1 (75) | | | 1 (75) |
| miR-98-5p | 1 (227) | | | | 1 (227) | | 1 (227) |
| miR-101-5p | 1 (158) | | | 1 (158) | | | 1 (158) |
| miR-103 | 1 (66) | | 1 (66) | | 1 (66) | | 1 (66) |
| miR-104 | 1 (222) | | | 1 (222) | | | 1 (222) |
| miR-106a | 3 (25,60,171) | | | 2 (60,171) | | 1 (25) | 1 (60) |
| miR-122 | 1 (248) | | | 1 (248) | | | 1 (248) |
| miR-122-5p | 1 (56) | | | | 1 (56) | | |
| miR-124* | 1 (250) | 1 (250) | | 1 (250) | | | 1 (250) |
| miR-124-3p | 1 (18) | | | 1 (18) | | | 1 (18) |
| miR-125b | 6 (22,25,54,185,235,237) | 3  (22,54,237) | 1 (185) | | 3 (22,185,237) | 2 (25,237) | 2 (62,185) |
| miR-125b-1-3p | 2 (60,62) | | | 2 (60,62) | | | 2 (60,62) |
| miR-126 | 3 (8,187,256) | 2 (8,256) | 2 (8,256) | 1 (8) | 2 (187,256) | | 2 (8,187) |
| miR-126* | 1 (238) | | | 1 (238) | | | |
| miR-126-3p | 2 (99,232) | | 1 (99) | 1  (99) | 1 (232) | | 1 (99) |
| miR-126-5p | 1 (232) | | | | 1 (232) | | |
| miR-128-3p | 1 (119) | | | 1 (119) | | | 1 (119) |
| miR-128a | 1 (222) | | | 1 (222) | | | 1 (222) |
| miR-130a | 1 (66) | | 1 (66) | | 1 (66) | | 1 (66) |
| miR-130a-3p | 1 (25) | | | | | 1 (25) | |
| miR-130b | 2 (66,250) | 1 (250) | 1 (66) | 1 (250) | 1 (66) | | 2 (66,250) |
| miR-132-3p | 1 (180) | | | | 1 (180) | | 1 (180) |
| miR-133b | 2 (102,222) | 1(102) | | 2 (102,222) | | | 2 (102,222) |
| miR-136 | 1 (159) | | | 1 (159) | | | 1 (159) |
| miR-137 | 1 (91) | 1 (91) | 1 (91) | 1 (91) | | | |
| miR-138 | 1 (165) | | | 1 (165) | | | 1 (165) |
| miR-141 | 3 (65,221,253) | | 1 (221) | 1 (221) | 2 (65,253) | | 3 (65,221,253) |
| miR-141-3p | 1 (80) | | | 1 (80) | 1 (80) | | |
| miR-142-3p | 1 (232) | | | 1 (232) | 1 (232) | | |

| | | | | | | | |
|---|---|---|---|---|---|---|---|
| miR-142-5p | 1 (232) | | | | 1 (232) | | |
| miR-143 | 1 (237) | 1 (237) | | | | 1 (237) | |
| miR-144* | 1 (238) | | | 1 (238) | | | |
| miR-144-5p | 1 (232) | | | | 1 (232) | | |
| miR-145 | 1 (66) | | 1 (66) | | 1 (66) | | 1 (66) |
| miR-145-5p | 1 (99) | | 1 (99) | 1 (99) | | | 1 (99) |
| miR-146-5p | 1 (217) | | | 1 (217) | | | |
| miR-146a | 1 (73) | | | 1 (73) | | | 1 (73) |
| miR-146a-5p | 1 (232) | | | | 1 (232) | | |
| miR-146b-5p | 3 (149,232,238) | | | 1 (238) | 2 (149,232) | 1 (149) | |
| miR-148a-3p | 1 (55) | | | 1 (55) | | | 1 (55) |
| miR-151 | 1 (64) | | | 1 (64) | | | |
| miR-151-3p | 1 (66) | | 1 (66) | | 1 (66) | | 1 (66) |
| miR-151a-3p | 1 (232) | | | | 1 (232) | | |
| miR-151a-5p | 1 (232) | | | | 1 (232) | | |
| miR-152 | 3 (57,69,236) | | | 1 (69) | 2 (57,236) | | 2 (57,236) |
| miR-154* | 1 (69) | | | 1 (69) | | | 1 (69) |
| miR-155 | 16 (20,51,63,88,95,155,175, 189,194,197,201,207,223, 244,248,250) | 3 (51,201,247) | 2 (88,201) | 10 (20,63,88,95,155, 175,197,201,248, 250) | 7 (51,189,194,207, 227,244,247) | | 12 (51,63,155,175,189,194, 197,207,227,247,248,250) |
| miR-155-5p | 5 (186,209,227,247,262) | 1 (186) | 1 (186) | 1 (209) | 4 (186,227,247,262) | | 4 (186,227,247,262) |
| miR-181a | 9 (60,64,66,68,69,192,200, 222,255) | | 3 (66,68,226) | 7 (60,64,68,69,200, 222,255) | 2 (66,192) | | 6 (60,64,66,68,192,255) |
| miR-181a-2-3p | 1 (226) | 1 (226) | | 1 (226) | | | 1 (226) |
| miR-181a-5p | 4 (41,86,199,227) | 1 (199) | | 2 (41,86) | 2 (41,86) | | 3 (41,86,227) |
| miR-181b | 1 (175) | | | 1 (175) | | | 1 (175) |
| miR-182 | 3 (57,60,175) | | | 2 (60,175) | 1 (57) | | 3 (57,60,175) |
| miR-182* | 3 (25,30,37) | | | 3 (169,175,222) | | | 3 (169,175,222) |
| miR-182-3-p | 1 (252) | | | | 1 (252) | | 1 (252) |
| miR-182-5p | 2 (10,40) | | | 2 (10,40) | | | |
| miR-183 | 5 (15,17,26,57,175) | | | 4 (15,17,26,175) | 1 (57) | | 3 (26,57,175) |
| miR-185 | 1 (238) | | | 1 (238) | | | |
| miR-186 | 1 (192) | | | | 1 (192) | | 1 (192) |
| miR-186-5p | 2 (199,232) | 1 (199) | | | 2 (199,232) | | |
| miR-187-5p | 1 (217) | | | 1 (217) | | | |
| miR-191 | 1 (224) | | | 1 (224) | | | 1 (224) |
| miR-191-5p | 1 (25) | | | | | 1 (25) | |
| miR-192 | 1 (237) | 1 (237) | | | 1 (237) | 1 (237) | |
| miR-193a-3p | 1 (236) | | | | 1 (236) | | |
| miR-193b | 1 (64) | | | 1 (64) | | | |
| miR-193b* | 1 (238) | | | 1 (238) | | | |
| miR-193b_st | 1 (169) | | | 1 (169) | | | 1 (169) |
| miR-193b-3p | 1 (55) | | | 1 (55) | | | 1 (55) |
| miR-193b-5p | 2 (96,99) | | 1 (99) | 1 (99) | 1(96) | | |
| miR-195 | 1 (68) | | 1 (68) | 1 (68) | | | 1 (68) |
| miR-196b-5p | 1 (198) | | | | 1 (198) | | 1 (198) |
| miR-197 | 1 (224) | | | 1 (224) | | | 1 (224) |
| miR-197-3p | 1 (180) | | | | 1 (180) | | 1 (180) |
| miR-198 | 1 (224) | | | 1 (224) | | | 1 (224) |

| | | | | | | | | |
|---|---|---|---|---|---|---|---|---|
| miR-199a-3p | 1 (232) | | | | 1 (232) | | | |
| miR-200a | 1 (253) | | | | 1 (253) | | | 1 (253) |
| miR-200a-3p | 1 (80) | | | 1 (80) | 1 (80) | | | |
| miR-200b | 1 (175) | | | 1 (175) | | | | 1 (175) |
| miR-200b-3p | 1 (146) | | | 1 (146) | | | | 1 (146) |
| miR-200c | 2 (92,245) | 1 (245) | | 1 (92) | 1 (245) | | | 2 (92,245) |
| miR-202 | 1 (224) | | | 1 (224) | | | | 1 (224) |
| miR-202-3p_st | 1 (166) | | | 1 (166) | | | | |
| miR-202-5p | 1 (110) | | | 1 (110) | | | | 1 (110) |
| miR-203 | 1 (45) | | | 1 (45) | | | | 1 (45) |
| miR-204 | 2 (224,236) | | | 1 (224) | 1 (236) | | | 1 (224) |
| miR-204-5p | 1 (215) | | | | 1 (215) | | 1 (215) | |
| miR-206 | 3 (24,148,211) | | | 2 (148,211) | 1 (24) | | | 2 (148,211) |
| miR-206-5p | 1 (198) | | | | 1 (198) | | | 1 (198) |
| miR-210 | 32 (29,34,37,38,51,55,57,60, 64,67,69,70,94,168,169, 173-176,180,187,189,190, 207,219,222,231,236,238, 243,244,259,261) | 1 (51) | 2 (38,259) | 21 (29,34,37,38,55,60, 64,69,94,169,173-176,190,219,222, 231,238,259,261) | 13 (51,57,67,70,168, 180, 187, 189,207,227,236, 243,244) | 1 (236) | 3 (1,24,31) | 23 (10–15,17–22,24,25,27–35,38,40) |
| miR-210-3p | 3 (208,227,246) | | | | 3 (208,227,246) | | | 2 (208,227) |
| miR-210-5p | 2 (226,246) | 1 (226) | | 1 (226) | 1(246) | | | 1 (226) |
| miR-214-3p | 1 (262) | | | | 1 (262) | | | |
| miR-215 | 2 (236,244) | | | | 2 (236,244) | | | |
| miR-215-5p | 1 (27) | | | 1 (27) | | | | 1 (27) |
| miR-218 | 1 (52) | | | 1 (52) | | | | |
| miR-218-5p | 2 (132,180) | | | 2 (132,180) | | | | |
| miR-221 | 2 (65,66) | | 1 (66) | | 2 (65,66) | | | 2 (65,66) |
| miR-221-3p | 1 (232) | | | | 1 (232) | | | |
| miR-222 | 1 (68) | | 1 (68) | 1 (68) | | | | 1 (68) |
| miR-222-3p | 1 (227) | | | | 1 (227) | | | 1 (227) |
| miR-296 | 3 (64,69,163) | 1 (163) | 1 (163) | 3 (64,69,163) | 1 (163) | | 1 (163) | 2 (69,163) |
| miR-296-3p | 3 (174,224,227) | | | 2 (174,224) | 1 (227) | | | 3 (174,224,227) |
| miR-296-5p | 3 (99,224,236) | | 1 (99) | 2 (99,224) | | | | 2 (99,224) |
| miR-299 | 1 (87) | | | 1 (87) | | | | 1 (87) |
| miR-299-5p | 1 (56) | | | | 1 (56) | | | |
| miR-300 | 1 (2) | | | 1 (2) | 1 (2) | | | |
| miR-302* | 1 (222) | | | 1 (222) | | | | 1 (222) |
| miR-302a | 1 (103) | | | 1 (103) | | | | 1 (103) |
| miR-302c-3p | 1 (232) | | | | 1 (232) | | | |
| miR-320a | 1 (103) | 1 (103) | | | 1 (103) | 1 (103) | | |
| miR-323a-3p | 1 (232) | | | | 1 (232) | | | |
| miR-326 | 1 (232) | | | 1 (232) | | | | |
| miR-328 | 1 (180) | | | | 1 (180) | | | 1 (180) |
| miR-330-5p | 1 (109) | | | 1 (109) | 1 (109) | | | 1 (109) |
| miR-331-5p | 1 (220) | 1 (220) | 1 (220) | 1 (220) | | | | |
| miR-335 | 2 (68,93) | | 1 (68) | 2 (68,93) | | | | 1 (68) |
| miR-342-3p | 3 (66,81,224) | | 1 (66) | 2 (81,224) | 1 (66) | | | 3 (66,81,224) |
| miR-346 | 2 (116,254) | | | 2 (116,254) | 1 (254) | | | 2 (116,254) |
| miR-355 | 1 (60) | | | 1 (60) | | | | 1 (60) |
| miR-361-5p | 1 (232) | | | | 1 (232) | | | |

| | | | | | | | |
|---|---|---|---|---|---|---|---|
| miR-362 | 2 (64,69) | | | 2 (64,69) | | | 1 (69) |
| miR-365a-3p | 1 (55) | | | 1 (55) | | | 1 (55) |
| miR-367* | 1 (14) | 1 (14) | | 1 (14) | | | 1 (14) |
| miR-371a-5p | 1 (99) | | 1 (99) | 1 (99) | | | 1 (99) |
| miR-371-3p | 1 (142) | 1 (142) | 1 (142) | 1 (142) | | | 1 (142) |
| miR-374a-5p | 2 (25,232) | | | | 1 (232) | 1 (25) | |
| miR-375 | 1 (180) | | | | 1 (180) | | 1 (180) |
| miR-376c | 1 (35) | | | 1 (35) | 1 (35) | | 1 (35) |
| miR-377 | 1 (174) | | | 1 (174) | | | 1 (174) |
| miR-378a-3p | 1 (232) | | | | 1 (232) | | |
| miR-378c | 1 (242) | | 1 (242) | 1 (242) | | | 1 (242) |
| miR-383 | 1 (250) | 1 (250) | 1 (250) | 1 (250) | | | 1 (250) |
| miR-411-5p | 1 (232) | | | | 1 (232) | | |
| miR-422a | 1 (232) | | | | 1 (232) | | |
| miR-423-3p | 1 (250) | 1 (250) | | 1 (250) | | | 1 (250) |
| miR-423-5p | 1 (196) | 1 (196) | | | 1 (196) | | 1 (196) |
| miR-424 | 1 (60) | | | 1 (60) | | | 1 (60) |
| miR-425 | 1 (66) | | 1 (66) | | 1 (66) | | 1 (66) |
| miR-425* | 1 (182) | | 1 (182) | | | | 1 (182) |
| miR-431 | 2 (85,250) | 1 (250) | | 2 (85,250) | | | 2 (85,250) |
| miR-432-3p | 1 (232) | | | | 1 (232) | | |
| miR-441 | 1 (35) | | | 1 (35) | 1 (35) | | 1 (35) |
| miR-450a | 1 (68) | | 1 (68) | 1 (68) | | | |
| miR-451 | 1 (238) | | 1 (238) | 1 (238) | | | |
| miR-451a | 1 (56) | | | | 1 (56) | | |
| miR-451_st | 1 (166) | | | 1 (166) | | | |
| miR-452 | 1 (188) | 1 (188) | | 1 (188) | | | |
| miR-483-5p | 1 (174) | | | 1 (174) | | | 1 (174) |
| miR-486-3p | 1 (68) | | 1 (68) | 1 (68) | | | |
| miR-490-3p | 1 (232) | | | | 1 (232) | | |
| miR-491-5p | 1 (13) | | | 1 (13) | | | |
| miR-494 | 2 (152,207) | | | 1 (152) | 1 (207) | | 2 (152,207) |
| miR-499a-5p | 1 (204) | | | 1 (204) | | | 1 (204) |
| miR-500a | 1 (250) | | | 1 (250) | | | 1 (250) |
| miR-502-5p | 1 (198) | | | | 1 (198) | | 1 (198) |
| miR-503-5p | 1 (198) | | | | 1 (198) | | 1 (198) |
| miR-507 | 1 (130) | | | 1 (130) | | | |
| miR-510 | 1 (216) | | | | 1 (216) | | |
| miR-512-3p | 3 (99,232,234) | | | 1 (99) | 2 (232,234) | | 1 (99) |
| miR-513c-5p | 1 (99) | | 1 (99) | 1 (99) | | | 1 (99) |
| miR-514b-3p | 1 (242) | | 1 (242) | 1 (242) | | | 1 (242) |
| miR-515-3p | 1 (64) | | | 1 (64) | | | |
| miR-515-5p | 2 (239,240) | | | | 2 (239,240) | | 2 (239,240) |
| miR-516-5 | 1 (206) | | | | 1 (206) | | |
| miR-516a-5p | 2 (172,239) | | | 1 (172) | 1 (239) | | 2 (172,239) |
| miR-516b | 1 (239) | | | | 1 (239) | | 1 (239) |
| miR-516b-5p | 1 (55) | | | 1 (55) | | | 1 (55) |
| miR-517 | 1 (231) | | | 1 (231) | | | 1 (231) |
| miR-517* | 2 (69,206) | | | 1 (206) | 1 (69) | | 1 (69) |
| miR-517-5p | 2 (39,202) | | | 1 (202) | 1 (39) | 1 (202) | |

| | | | | | | | | |
|---|---|---|---|---|---|---|---|---|
| miR-517a/b | 1 (167) | | | 1 (167) | | | | 1 (167) |
| miR-517c | 2  (167,238) | | | 2 (167,238) | | | | 1 (167) |
| miR-517c-3p | 1 (208) | | | | 1 (208) | | | 1 (208) |
| miR-518a-5p | 2 (150,250) | 1  (250) | | 2 (150,250) | | | | 2 (150,250) |
| miR-518b | 9 (64,69,179,183,186,202, 236,239,260) | 5 (179,183,186,239, 260) | 2 (186,260) | 3 (68,69,183) | 7 (179,183,186, 202,236,239,260) | 1 (236) | | 4 (69,179,186,239) |
| miR-518c | 1 (238) | | | 1 (238) | | | | |
| miR-518f | 1 (234) | | | | 1 (234) | | | |
| miR-518f* | 1 (238) | | | 1 (238) | | | | |
| miR-518f-3p | 1 (222) | | | | 1 (222) | | | |
| miR-519a | 1 (200) | 1 (200) | | 1 (200) | | | | |
| miR-519a-3p | 1 (196) | 1 (196) | 1 (196) | | 1 (196) | | | 1 (196) |
| miR-519b-3p | 1 (68) | | 1 (68) | 1 (68) | | | | |
| miR-519d | 1 (239) | | | | 1 (239) | | | 1 (239) |
| miR-519d-3p | 1 (59) | | | | | | | 1 (163) |
| miR-519-d-5p | 1 (252) | | | | 1 (252) | | | 1 (252) |
| miR-519e* | 3 (64,69,238) | | | 3 (64,69,238) | | | | 1 (69) |
| miR-520h | 1 (202) | | | | 1 (202) | 1 (202) | | 1 (202) |
| miR-520a | 1 (236) | | | | 1 (236) | | | |
| miR-520a* | 1 (238) | | | | 1 (238) | | | |
| miR-520a-3p | 1 (238) | | | 1 (238) | | | | |
| miR-520a-5p | 2 (55,239) | | | 1 (55) | 1 (202) | | | 2 (55,239) |
| miR-520c-3p | 2 (232,234) | | | | 2 (232,234) | | | |
| miR-520d-3p | 2 (232,234) | | | | 2 (232,234) | | | |
| miR-520g | 1 (89) | | 1 (89) | 1 (89) | 1 (89) | | | |
| miR-520h | 1 (202) | | | | 1 (202) | | | |
| miR-522 | 1 (68) | | 1 (68) | 1 (68) | | | | |
| miR-524 | 1 (64) | | | 1 (64) | | | | 1 (64) |
| miR-524-3p | 1 (172) | | | 1 (172) | | | | |
| miR-525 | 1 (205) | | | | 1 (205) | | | |
| miR-525* | 1 (64) | | | 1 (64) | | | | 1 (64) |
| miR-525-5p | 2 (11,239) | | | 1 (239) | 1 (11) | | | |
| miR-526a | 1 (206) | | | | 1 (206) | | | |
| miR-526b | 1 (239) | | | | 1 (239) | | | 1 (239) |
| miR-526b* | 1 (238) | | | 1 (238) | | | | |
| miR-548aj_st | 1 (166) | | | 1 (166) | | | | |
| miR-565 | 1 (68) | | 1 (68) | 1 (68) | | | | |
| miR-572 | 1 (232) | | | | 1 (232) | | | |
| miR-573 | 1 (232) | | | | 1 (232) | | | |
| miR-574 | 1 (25) | | | 1 (25) | | | | 1 (25) |
| miR-574-5p | 5 (25,66,144,246,258) | 1 (258) | 1 (66) | 1 (144) | 3 (66,246,258) | 1 (25) | | 2 (66,258) |
| miR-582-3p | 1 (254) | | | | 1 (254) | | | 1 (254) |
| miR-584 | 3 (64,69,93) | | | 3 (64,69,93) | | | | 1 (69) |
| miR-584-5p | 1 (232) | | | | 1 (232) | | | |
| miR-590-5p | 1 (232) | | | 1 (232) | 1 (232) | | | |
| miR-601 | 1 (232) | | | | 1 (232) | | | |
| miR-628-3p | 2 (232,233) | | | | 2 (232,233) | | | |
| miR-628-5p | 2 (232,233) | | | | 2 (232,233) | | | |
| miR-629-5p | 1 (196) | 1 (196) | | | 1 (196) | | | 1 (196) |
| miR-638 | 2 (64,69) | | | 2 (64,69) | | | | 1 (69) |

| | | | | | | | | |
|---|---|---|---|---|---|---|---|---|
| miR-650 | 2 (236,244) | | | | 2 (236,244) | | | |
| miR-655-3p | 1 (16) | | | 1 (16) | | | | |
| miR-663 | 1 (242) | 1 (242) | | 1 (242) | | | | 1 (242) |
| miR-664a-3p | 1 (232) | | | | 1 (232) | | | |
| miR-758-3p | 1 (198) | | | | 1 (198) | | | 1 (198) |
| miR-766-3p | 1 (232) | | | | 1 (232) | | | |
| miR-875-5p | 1 (232) | | | | 1 (232) | | | |
| miR-885-5p | 1 (212) | | | | 1 (212) | | | 1 (212) |
| miR-892b | 1 (232) | | | | 1 (232) | | | |
| miR-892c-3p | 1 (242) | | 1 (242) | 1 (242) | | | | 1 (242) |
| miR-1183 | 2 (182,250) | | 2 (182,250) | 2 (182,250) | | | | 2 (182,250) |
| miR-1233 | 1 (236) | | | | 1 (236) | | | |
| miR-1233-3p | 1 (246) | | | | 1 (246) | | | |
| miR-1247-5p | 1 (232) | | | | 1 (232) | | | |
| miR-1299 | 1 (56) | | | | 1 (56) | | | |
| miR-1304-5p | 1 (33) | | | | 1 (33) | 1 (33) | | |
| miR-1305 | 1 (239) | | | 1 (239) | | | | 1 (239) |
| miR-1323 | 1 (239) | | | | 1 (239) | | | 1 (239) |
| miR-1469 | 1 (60) | | | 1 (60) | | | | 1 (60) |
| miR-1972 | 1 (258) | 1 (258) | | | 1 (258) | | | 1 (258) |
| miR-2277 | 1 (172) | | | 1 (172) | | | | 1 (172) |
| miR-4443 | 1 (135) | | | 1 (135) | | | | 1 (135) |
| miR-4793-3p | 2 (220,258) | 2 (220,258) | 2 (220,258) | 1 (220) | 1 (258) | | | 1 (258) |
| miR-5002-5p | 1 (33) | | | | 1 (33) | 1 (33) | | |

**Table S5.** Downregulated microRNAs in preeclampsia. See the reference list at the end of the supplementary tables.

| microRNA | General | Early | Late | Placenta | Blood | 1st trimester | 2nd trimester | 3rd trimester |
|---|---|---|---|---|---|---|---|---|
| ebv-miR-BART1-5p | 1 (33) | | | | 1 (33) | 1 (33) | | |
| hiv1-miR-TAR-3p | 1 (33) | | | | 1 (33) | 1 (33) | | |
| Let-7a-5p | 1 (214) | | | | 1 (214) | | | 1 (214) |
| let-7c-5p | 1 (196) | 1 (196) | 1 (196) | 1 (196) | | | | 1 (196) |
| let-7f_st | 1 (169) | | | 1 (169) | | | | 1 (169) |
| let-7i | 1 (90) | | | 1 (90) | | | | 1 (90) |
| miR-1 | 3 (69,173,185) | | 1 (185) | 3 (69,173,185) | | | | 3 (69,173,185) |
| miR-7_st | 1 (169) | | | 1 (169) | | | | 1 (169) |
| miR-10b | 1 (69) | | | 1 (69) | | | | 1 (69) |
| miR-15a-3p | 1 (56) | | | | 1 (56) | | | |
| miR-15b | 1 (236) | | | | 1 (236) | | | |
| miR-16 | 3 (66,241,245) | 2 (241,245) | 1 (66) | | 3 (66,241,245) | | | 3 (66,241,245) |
| miR-16-5p | 1 (227) | | | | 1 (227) | | | 1 (227) |
| miR-17-5p | 1 (180) | | | | 1 (180) | | | 1 (180) |
| miR-18a | 5 (64,69,211,241,244) | 1 (241) | | 3 (64,69,211) | 3 (64,241,244) | | | 4 (64,69,211,241) |
| miR-18b | 4 (47,60,69,232) | | | 4 (47,60,69,232) | | | | 4 (47,60,69,232) |

| | | | | | | | | |
|---|---|---|---|---|---|---|---|---|
| miR-19a | 3 (64,69,187) | | | 2 (64,69) | 1 (187) | | | 2 (69,187) |
| miR-19a-3p | 2 (42,212) | | | 1 (42) | 1 (212) | | | 1 (212) |
| miR-19b | 1 (60) | | | 1 (60) | | | | 1 (60) |
| miR-19b1 | 2 (64,244) | | | | 2 (64,244) | | | |
| miR-19b-3p | 2 (187,192,212) | | | 1 (192) | 2 (187,212) | | | 2 (187,212) |
| miR-20a-star_st | 1 (169) | | | 1 (169) | | | | 1 (169) |
| miR-21 | 3 (30,163,224) | | 1 (30) | 2 (163,224) | 1 (30) | | | 1 (163) |
| miR-21-3p | 1 (184) | | | | 1 (184) | | | 1 (184) |
| miR-22-5p | 1 (25) | | | | | 1 (25) | | |
| miR-23a* | 1 (198) | | | | 1 (198) | | | |
| miR-23b-5p | 1 (249) | | 1 (249) | | 1 (249) | | | |
| miR-24-1-star_st | 1 (169) | | | 1 (169) | | | | 1 (169) |
| miR-26b_st | 1 (169) | | | 1 (169) | | | | 1 (169) |
| miR-27b-3p | 1 (232) | 1 (232) | 1 (232) | 1 (232) | | | | |
| miR-29a-3p | 1 (60) | | | 1 (60) | | | | 1 (60) |
| miR-29b | 1 (207) | | | | 1 (207) | | | 1 (207) |
| miR-29c-star_st | 1 (169) | | | 1 (169) | | | | 1 (169) |
| miR-30d-star_st | 1 (169) | | | 1 (169) | | | | 1 (169) |
| miR-31 | 1 (30) | 1 (30) | | | 1 (30) | | | |
| miR-31-3p | 1 (56) | | | | 1 (56) | | | |
| miR-32 | 1 (69) | | | 1 (69) | | | | 1 (69) |
| miR-34c-5p | 2 (172,173) | | | 2 (172,173) | | | | 2 (172,173) |
| miR-92a | 1 (60) | | | 1 (60) | | | | 1 (60) |
| miR-92a1 | 1 (64) | | | | 1 (64) | | | |
| miR-92a-2-5p | 1 (33) | | | | 1 (33) | 1 (33) | | |
| miR-93-5p | 1 (25) | | | | | 1 (25) | | |
| miR-99b-5p | 1 (249) | | 1 (249) | | 1 (249) | | | |
| miR-100-5p | 1 (203) | | | | 1 (203) | | | |
| miR-101 | 2 (69,121) | | | 2 (69,121) | | | | 2 (69,121) |
| miR-101_st | 1 (169) | | | 1 (169) | | | | 1 (169) |
| miR-107 | 1 (33) | | | | 1 (33) | 1 (33) | | |
| miR-125a-5p | 1 (51) | 1 (51) | | | 1 (51) | | | 1 (51) |
| miR-125b | 1 (185) | | 1 (185) | 1 (185) | | | | 1 (185) |
| miR-125b-5p | 1 (203) | | | | 1 (203) | | | |
| miR-126 | 3 (61,70,236) | | | 1 (61) | 2 (61,70) | | | |
| miR-126# | 1 (237) | 1 (237) | | | 1 (237) | 1 (237) | | |
| miR-126* | 2 (69,250) | | | 2 (69,250) | | | | 2 (69,250) |
| miR-126-3p | 2 (25,72) | | | 1 (72) | | 1 (25) | | 1 (72) |
| miR-127 | 1 (237) | 1 (237) | | | 1 (237) | 1 (237) | | |
| miR-127-3p | 1 (196) | 1 (196) | 1 (196) | 1 (196) | | | | 1 (196) |
| miR-128_st | 1 (169) | | | 1 (169) | | | | 1 (169) |
| miR-133a | 1 (124) | | | | 1 (124) | | 1 (124) | |
| miR-133a-3p | 1 (232) | | | | 1 (232) | | | |

| | | | | | | | | |
|---|---|---|---|---|---|---|---|---|
| miR-134-5p | 1 (133) | | | 1 (133) | | | | 1 (133) |
| miR-135 | 1 (3) | | | 1 (3) | | | | 1 (3) |
| miR-135a | 1(156) | 1 (156) | | 1(156) | | | | |
| miR-135a-5p | 1 (131) | | | 1 (131) | | | | 1 (131) |
| miR-135b-5p | 1 (55) | | | 1 (55) | | | | 1 (55) |
| miR-136-3p | 1 (55) | | | 1 (55) | | | | 1 (55) |
| miR-137_st | 1 (169) | | | 1 (169) | | | | 1 (169) |
| miR-138-5p | 1 (9) | | | 1 (9) | | | | |
| miR-139-5p | 2 (128,173) | | | 2 (128,173) | | | | 2 (128,173) |
| miR-140-5p | 1 (117) | 1 (117) | | 1 (117) | | | | |
| miR-140-5p_st | 1 (169) | | | 1 (169) | | | | 1 (169) |
| miR-141-5p | 1 (125) | | | 1 (125) | | | | 1 (125) |
| miR-144 | 6 (50,65,66,69,236, 244) | | 1 (66) | 2 (50,69) | 3 (65,66,236) | | | 4 (50,65,66,69) |
| miR-145 | 1 (83) | | | | | | | |
| miR-145-5p | 1 (28) | | | 1 (28) | | | | 1 (28) |
| miR-145-star_st | 1 (169) | | | 1 (169) | | | | 1 (169) |
| miR-146-5p | 1 (217) | | | 1 (217) | 1 (217) | | | |
| miR-146a | 3 (172,197,201) | 1 (201) | 2 (197, 201) | 3 (172,197,201) | | | | 2 (172,197) |
| miR-148a | 1 (1) | | | 1 (1) | | | | 1 (1) |
| miR-148b* | 1 (14) | 1 (14) | | | | | | 1 (14) |
| miR-149 | 3 (170,193,241) | 2 (170,241) | | 1 (6) | 2 (193,241) | | 1 (193) | 3 (170,193,241) |
| miR-149-5p | 3 (6,78,220) | 1 (220) | 1 (220) | 3 (6,78,220) | | | | 2 (6,78) |
| miR-150 | 1 (69) | | | 1 (69) | | | | 1 (69) |
| miR-151-3p | 1 (172) | | | 1 (172) | | | | |
| miR-154* | 1 (69) | | | 1 (69) | | | | 1 (69) |
| miR-155 | 1 (197) | | 1 (197) | 1 (197) | | | | 1 (197) |
| miR-155-5p | 1 (184) | | | | 1 (184) | | | 1 (184) |
| miR-181a | 1 (200) | 1 (200) | | 1 (200) | | | | |
| miR-186-5p | 1 (199) | 1 (199) | 1 (199) | 1 (199) | | | | |
| miR-187-5p | 1 (217) | | | 1 (217) | 1 (217) | | | |
| miR-188-3p | 2 (33,162) | | | 1 (162) | 1 (33) | 1 (33) | | |
| miR-191-5p | 1 (180) | | | | 1 (180) | | | 1 (180) |
| miR-192 | 1 (172) | | | 1 (172) | | | | 1 (172) |
| miR-192_st | 1 (169) | | | 1 (169) | | | | |
| miR-194 | 1 (170) | | | 1 (170) | | | | |
| pri-miR-195 | 1 (120) | | | 1 (120) | | | | 1 (120) |
| miR-195 | 5 (64,69,120,136,181) | | 1 (181) | 4 (64,69,120,136) | 1 (181) | | | 4 (69,120,136,181) |
| miR-195-5p | 1 (161) | | | | 1 (161) | | | 1 (161) |
| miR-196a_st | 1 (169) | | | 1 (169) | | | | 1 (169) |
| miR-196a-5p | 1 (76) | | | 1 (76) | | | | |
| miR-196b | 1 (213) | | | | 1 (213) | | | 1 (213) |
| miR-196b_st | 1 (169) | | | 1 (169) | | | | 1 (169) |

| | | | | | | | | |
|---|---|---|---|---|---|---|---|---|
| miR-197-3p | 1 (232) | | | | 1 (232) | | | |
| miR-199b-5p_st | 1 (169) | | | 1 (169) | | | | 1 (169) |
| miR-200c | 2 (60,174) | | | 2 (60,174) | | | | 2 (60,174) |
| miR-203a-3p | 1 (33) | | | | 1 (33) | 1 (33) | | |
| miR-204 | 2 (23,261) | | | 1 (224) | 1 (236) | 1 (236) | 1 (236) | 1 (224) |
| miR-204-3p | 1 (25) | | | | | 1 (25) | | |
| miR-206 | 1 (124) | | | | 1 (124) | | 1 (124) | |
| miR-210 | 1 (263) | | | | 1 (263) | | 1 (263) | |
| miR-211-5p | 1 (33) | | | | 1 (33) | 1 (33) | | |
| miR-214 | 2 (64,68) | | 1 (68) | 2 (64,68) | | | | 1 (68) |
| miR-214-star_st | 1 (169) | | | 1 (169) | | | | 1 (169) |
| miR-217 | 1 (146) | | | 1 (146) | | | | 1 (146) |
| miR-218 | 2 (69,228) | | | 2 (69,228) | | | | |
| miR-218_st | 1 (169) | | | 1 (169) | | | | 1 (169) |
| miR-218-5p | 1 (228) | | | 1 (228) | | | | 1 (228) |
| miR-221 | 1 (237) | 1 (237) | | | 1 (237) | 1 (237) | | |
| miR-221-3p | 1 (126) | | | 1 (126) | | | | 1 (126) |
| miR-221-star_st | 1 (169) | | | 1 (169) | | | | 1 (169) |
| miR-222 | 1 (255) | | | | 1 (255) | | | 1 (255) |
| miR-222-5p | 1 (12) | | | 1 (12) | | | | |
| miR-223 | 3 (64,69,224) | | | 3 (64,69,224) | | | | 1 (69) |
| miR-223_st | 1 (169) | | | 1 (169) | | | | 1 (169) |
| miR-223-3p | 2 (148,259) | 1 (259) | | 2 (148,259) | | | | 2 (148,259) |
| miR-224 | 1 (238) | | | 1 (238) | | | | |
| miR-224_st | 1 (169) | | | 1 (169) | | | | 1 (169) |
| miR-224-5p | 1 (259) | 1 (259) | | 1 (259) | | | | 1 (259) |
| miR-302b-3p | 1 (180) | | | | 1 (180) | | | 1 (180) |
| miR-320a | 1 (32) | | | 1 (32) | | | | 1 (32) |
| mir-320b-1_x_st | 1 (166) | | | 1 (166) | | | | |
| miR-325 | 2 (229,230) | | | 2 (229,230) | | | | |
| miR-328 | 1 (230) | | | 1 (230) | | | | 1 (230) |
| miR-329_st | 1 (169) | | | 1 (169) | | | | 1 (169) |
| miR-335 | 2 (60,236) | | | 1 (60) | 1 (236) | | | 1 (60) |
| miR-345-3p | 1 (106) | | | | 1 (106) | | | 1 (106) |
| miR-346 | 1 (254) | | | 1 (254) | | | | 1 (254) |
| miR-363 | 4 (60,64,69,241) | 1 (241) | | 2 (60,69) | 2 (64,241) | | | 3 (60,69,241) |
| miR-363_st | 1 (169) | | | 1 (169) | | | | 1 (169) |
| miR-365a-3p | 1 (25) | | | | 1 (25) | 1 (25) | | |
| miR-369-3p | 1 (33) | | | | | 1 (33) | | |
| miR-374 | 1 (69) | | | 1 (69) | | | | 1 (69) |
| miR-376a | 1 (236) | | | 1 (236) | 1 (236) | | | |
| miR-376c | 1 (43) | | | | 1 (43) | | | 1 (43) |
| miR-376c-3p | 1 (212) | | | | 1 (212) | | | 1 (212) |
| miR-377 | 1 (69) | | | 1 (69) | | | | 1 (69) |

| | | | | | | | |
|---|---|---|---|---|---|---|---|
| miR-378-3p | 1 (138) | | | 1 (138) | | | 1 (138) |
| miR-379 | 1 (64) | | | 1 (64) | | | |
| miR-383 | 1 (129) | | | 1 (129) | | | 1 (129) |
| miR-411 | 2 (64,69) | | | 2  (64,69) | | | 1 (69) |
| miR-411-star_st | 1 (169) | | | 1 (169) | | | 1 (169) |
| miR-423-5p | 3 (68,115,196) | 1 (196) | 1 (68) | 3 (68,115,196) | | | 2 (68,196) |
| miR-424 | 3 (31,219,241) | | | 2 (31,219) | 1 (241) | | 3 (31,219,241) |
| miR-424-3p | 1 (33) | | | | 1 (33) | 1 (33) | |
| miR-424-5p | 1 (118) | | | 1 (118) | | | |
| miR-432-star_st | 1 (166) | | | 1 (166) | | | |
| miR-450 | 1 (69) | | | 1 (69) | | | 1 (69) |
| miR-454 | 2 (44,84) | | | 2 (44,84) | | | 2 (44,84) |
| miR-454_st | 1 (169) | | | 1 (169) | | | 1 (169) |
| miR-455-3p | 1 (261) | | | 1 (261) | | | 1 (261) |
| miR-455-5p | 1 (261) | | | 1 (261) | | | 1 (261) |
| miR-483 | 1 (19) | 1 (19) | | 1 (19) | 1 (19) | | 1 (19) |
| miR-489_st | 1 (169) | | | 1 (169) | | | 1 (169) |
| miR-491-5p | 1 (68) | | 1 (68) | 1 (68) | | | 1 (68) |
| miR-493-star_st | 1 (169) | | | 1 (169) | | | 1 (169) |
| miR-500 | 1 (173) | | | 1 (173) | | | 1 (173) |
| miR-502-5p_st | 1 (169) | | | 1 (169) | | | 1 (169) |
| miR-505_st | 1 (169) | | | 1 (169) | | | 1 (169) |
| miR-506-5p | 1 (33) | | | | 1 (33) | 1 (33) | |
| miR-508-3p | 1 (218) | | | 1 (218) | | | 1 (218) |
| miR-508-5p | 1 (68) | | 1 (68) | 1 (68) | | | 1 (68) |
| miR-515-3p | 1 (177) | | | 1 (177) | | | 1 (177) |
| miR-515-5p | 2 (177,205) | | | 2 (177,205) | | | 2 (177,205) |
| miR-516a-5p | 1 (177) | | | 1 (177) | | | 1 (177) |
| miR-517-5p | 1 (205) | | | 1 (205) | | | 1 (205) |
| miR-517a/b | 1 (210) | | | 1 (210) | | | 1 (210) |
| miR-518b | 2 (177,205) | | | 2 (177,205) | | | 2 (177,205) |
| miR-518f | 1 (177) | | | 1 (177) | | | 1 (177) |
| miR-518f-5p | 1 (205) | | | 1 (205) | | | 1 (205) |
| miR-519a | 1 (205) | | | 1 (205) | | | 1 (205) |
| miR-519a-3p | 1 (196) | 1 (196) | 1 (196) | 1 (196) | | | 1 (196) |
| miR-519c-3p | 2 (169,177) | | | 1 (169,177) | | | 1 (169,177) |
| miR-519c-3p_st | 1 (169) | | | 1 (169) | | | 1 (169) |
| miR-519d | 2 (100,205) | | | 2 (100,205) | | | 2 (100,205) |
| miR-519e-5p | 2 (177) | | | 1 (177) | | | 1 (177) |
| miR-520a-3p | 1 (97) | 1 (97) | | 1 (97) | | | 1 (97) |
| miR-520a-5p | 2 (177,205) | | | 2 (177,205) | | | 2 (177,205) |
| miR-520b_st | 1 (169) | | | 1 (169) | | | 1 (169) |
| miR-520c-3p_st | 1  (169) | | | 1 (169) | | | 1 (169) |
| miR-520d-5p | 1 (177) | | | 1 (177) | | | 1 (177) |

| | | | | | | | | |
|---|---|---|---|---|---|---|---|---|
| miR-520f_st | 1 (169) | | | 1 (169) | | | | 1 (169) |
| miR-520h | 1 (205) | | | 1 (205) | | | | 1 (205) |
| miR-524-5p | 2 (177,205) | | | 2 (177,205) | | | | 2 (177,205) |
| miR-525 | 1 (205) | | | 1 (205) | | | | 1 (205) |
| miR-525-5p | 1 (11) | | | 1 (11) | | | | 1 (11) |
| miR-526a | 1 (205) | | | 1 (205) | | | | 1 (205) |
| miR-532-3p | 2 (68,115) | | 1 (68) | 2 (68,115) | | | | 1 (68) |
| miR-532-5p | 1 (196) | | 1 (196) | 1 (196) | | | | 1 (196) |
| miR-539-5p | 1 (196) | 1 (196) | | 1 (196) | | | | 1 (196) |
| miR-542-3p | 2 (64,69) | | | 2 (64,69) | | | | 1 (69) |
| miR-542-3p_st | 1 (169) | | | 1 (169) | | | | 1 (169) |
| miR-544 | 1 (250) | 1 (250) | | 1 (250) | | | | 1 (250) |
| miR-551b_st | 1 (169) | | | 1 (169) | | | | 1 (169) |
| miR-558 | 4 (107,111,114,141) | | | 4 (107,111,114,141) | | | | 2 (107,141) |
| miR-559-5p | 1 (25) | | | | | 1 (25) | | |
| miR-584 | 2 (60,173) | | | 2 (60,173) | | | | 2 (60,173) |
| miR-585_st | 1 (169) | | | 1 (169) | | | | 1 (169) |
| miR-590 | 2 (64,69) | | | 2 (64,69) | | | | 1 (69) |
| miR-612 | 1 (68) | | 1 (68) | 1 (68) | | | | 1 (68) |
| miR-625 | 1 (69) | | | 1 (69) | | | | 1 (69) |
| miR-629-5p | 1 (196) | 1 (196) | 1 (196) | 1 (196) | | | | 1 (196) |
| miR-642b-3p | 1 (33) | | | | 1 (33) | 1 (33) | | |
| miR-652-3p | 1 (37) | | | 1 (37) | | | | |
| miR-658 | 1 (68) | | 1 (68) | 1 (68) | | | | 1 (68) |
| miR-668 | 1 (236) | | | | 1 (236) | | | |
| miR-744 | 1 (60) | | | 1 (60) | | | | 1 (60) |
| miR-762 | 1 (71) | | | 1 (71) | | | | 1 (71) |
| miR-892b | 1 (33) | | | | 1 (33) | 1 (33) | | |
| miR-933_st | 1 (166) | | | 1 (166) | | | | |
| miR-937 | 1 (14) | 1 (14) | | 1 (14) | | | | 1 (14) |
| miR-942 | 2 (122,237) | 1 (237) | | | 2 (122,237) | 1 (237) | 1 (122) | |
| miR-942-5p | 3 (112,157,160) | | | 3 (112,157,160) | | | | 1 (160) |
| miR-1247 | 1 (173) | | | 1 (173) | | | | 1 (173) |
| miR-1273c | 1 (33) | | | | 1 (33) | 1 (33) | | |
| miR-1283 | 1 (241) | | | | 1 (241) | | | 1 (241) |
| miR-1290-3p | 1 (262) | | | | 1 (262) | | | |
| miR-1301 | 1 (259) | 1 (259) | | 1 (259) | | | | 1 (259) |
| miR-1826 | 1 (60) | | | 1 (60) | | | | 1 (60) |
| miR-2392 | 1 (33) | | | | 1 (33) | 1 (33) | | |
| miR-3064-5p | 1 (33) | | | | 1 (33) | 1 (33) | | |
| miR-3171 | 1 (33) | | | | 1 (33) | 1 (33) | | |
| miR-3184-5p | 1 (33) | | | | 1 (33) | 1 (33) | | |
| miR-3649 | 1 (33) | | | | 1 (33) | 1 (33) | | |
| miR-3907 | 1 (14) | 1 (14) | | 1 (14) | | | | 1 (14) |

| | | | | | | |
|---|---|---|---|---|---|---|
| miR-3935 | 1 (191) | | | | | 1 (191) |
| miR-3942 | 1 (250) | 1 (250) | 1 (250) | | | 1 (250) |
| miR-4264-5p | 1 (25) | | | | 1 (25) | |
| miR-4329 | 1 (33) | | | 1 (33) | 1 (33) | |
| miR-4432 | 1 (33) | | | 1 (33) | 1 (33) | |
| miR-4482-3p | 1 (33) | | | 1 (33) | 1 (33) | |
| miR-4498 | 1 (33) | | | 1 (33) | 1 (33) | |
| miR-4701-3p_st | 1 (166) | | 1 (166) | | | |
| miR-4752 | 1 (56) | | | 1 (56) | | |
| miR-4758-5p | 1 (33) | | | 1 (33) | 1 (33) | |
| miR-4785 | 1 (56) | | | 1 (56) | | |
| miR-5000-5p | 1 (33) | | | 1 (33) | 1 (33) | |
| miR-5006-3p | 1 (160) | | 1 (160) | | | 1 (160) |
| miR-5009-3p | 1 (33) | | | 1 (33) | 1 (33) | |
| miR-5582-3p | 1 (33) | | | 1 (33) | 1 (33) | |

## Supplementary References

1. Luo S, Li H, Cao N, Tang Y, Gu W. MicroRNA-148a affects functions of placental trophoblast cells in preeclampsia by regulating HLA-G. Int J Clin Exp Pathol. 2017;10(5):5205–12.

2. Gao S, Wang Y, Han S, Zhang Q. Up-regulated microRNA-300 in maternal whole peripheral blood and placenta associated with pregnancy-induced hypertension and preeclampsia. Int J Clin Exp Pathol. 2017;10(4):4232–42.

3. Zhao X, Zhang X, Wu Z, Mei J, Li L, Wang Y. Up-regulation of microRNA-135 or silencing of PCSK6 attenuates inflammatory response in preeclampsia by restricting NLRP3 inflammasome. Molecular Medicine. 2021 Dec 23;27(1):82.

4. Liao G, Cheng D, Li J, Hu S. Clinical significance of microRNA-320a and insulin-like growth factor-1 receptor in early-onset preeclampsia patients. European Journal of Obstetrics & Gynecology and Reproductive Biology. 2021 Aug;263:164–70.

5. Zhao X, Liu F, Zhang J, Zhang J, Zhang L, Chen L. LINC01128 - miR-16 interaction regulates the migration and invasion of human chorionic trophoblast cells. Hypertens Pregnancy. 2021 Apr 3;40(2):152–61.

6. Hu Z, Dong C, Dong Q. Circ_0015382 is associated with preeclampsia and regulates biological behaviors of trophoblast cells through miR-149-5p/TFPI2 axis. Placenta. 2021 May;108:73–80.

7. Zhu L, Liu Z. Serum from patients with hypertension promotes endothelial dysfunction to induce trophoblast invasion through the miR 27b 3p/ATPase plasma membrane Ca 2+ transporting 1 axis. Mol Med Rep. 2021 Mar 3;23(5):319.

8. Liu B, Liu L, Cui S, Qi Y, Wang T. Expression and significance of <scp>microRNA</scp> -126 and <scp>VCAM</scp> -1 in placental tissues of women with early-onset preeclampsia. Journal of Obstetrics and Gynaecology Research. 2021 Jun 10;47(6):2042–50.

9. Yin A, Chen Q, Zhong M, Jia B. MicroRNA-138 improves LPS-induced trophoblast dysfunction through targeting RELA and NF-κB signaling. Cell Cycle. 2021 Mar 19;20(5–6):508–21.

10. Gai S, Sun L, Wang H, Yang P. Circular RNA hsa_circ_0007121 regulates proliferation, migration, invasion, and epithelial–mesenchymal transition of trophoblast cells by miR-182-5p/PGF axis in preeclampsia. Open Medicine. 2020 Oct 14;15(1):1061–71.

11. Zhang M, Li P, Mao X, Zhang H. Regulatory mechanism of <scp>miR</scp> ‐525‐5p in over‐invasion of trophoblast. Journal of Obstetrics and Gynaecology Research. 2021 Feb;47(2):679–88.

12. Dong X, Zhao J, Han J, Han XJ, Zhao CM, Zou AX, et al. MiR-222-5p promotes the growth and migration of trophoblasts by targeting AHNAK. Eur Rev Med Pharmacol Sci. 2020 Nov;24(21):10954–9.

13. Liu E, Zhou Y, Li J, Zhang D. MicroRNA 491 5p inhibits trophoblast cell migration and invasion through targeting matrix metalloproteinase 9 in preeclampsia. Mol Med Rep. 2020 Oct 14;22(6):5033–40.

14. Zhang H, Xue L, Lv Y, Yu X, Zheng Y, Miao Z, et al. Integrated microarray analysis of key genes and a miRNA mRNA regulatory network of early onset preeclampsia. Mol Med Rep. 2020 Sep 30;22(6):4772–82.

15. Zhu H, Wang C. Retracted: HDAC2‐mediated proliferation of trophoblast cells requires the miR‐183/FOXA1/IL‐8 signaling pathway. J Cell Physiol. 2021 Apr 8;236(4):2544–58.

16. Song H, Wang X, Li JC, Lv YH. MiR-655-3p inhibits growth and invasiveness of trophoblasts via targeting PBX3 and thus deteriorates preeclampsia. Eur Rev Med Pharmacol Sci. 2020 Oct;24(20):10346–51.

17. Lai W, Yu L. Elevated MicroRNA 183 Impairs Trophoblast Migration and Invasiveness by Downregulating FOXP1 Expression and Elevating GNG7 Expression during Preeclampsia. Mol Cell Biol. 2021 Jan 1;41(1).

18. Tao J, Xia LZ, Liang L, Chen Y, Wei D, Meng J, et al. MiR-124–3p promotes trophoblast cell HTR-8/SVneo pyroptosis by targeting placental growth factor. Placenta. 2020 Nov;101:176–84.

19. Han L, Luo QQ, Peng MG, Zhang Y, Zhu XH. miR-483 is downregulated in pre-eclampsia via targeting insulin-like growth factor 1 (IGF1) and regulates the PI3K/Akt/mTOR pathway of endothelial progenitor cells. J Obstet Gynaecol Res. 2021 Jan;47(1):63–72.

20. Wang Z, Shan Y, Yang Y, Wang T, Guo Z. MicroRNA-155 is upregulated in the placentas of patients with preeclampsia and affects trophoblast apoptosis by targeting SHH/GLi1/BCL2. Hum Exp Toxicol. 2021 Mar 10;40(3):439–51.

21. Fan Y, Dong Z, Zhou G, Fu J, Zhan L, Gao M, et al. Elevated miR-23a impairs trophoblast migration and invasiveness through HDAC2 inhibition and NF-κB activation. Life Sci. 2020 Nov;261:118358.

22. Tang J, Wang D, Lu J, Zhou X. MiR-125b participates in the occurrence of preeclampsia by regulating the migration and invasion of extravillous trophoblastic cells through STAT3 signaling pathway. Journal of Receptors and Signal Transduction. 2021 Mar 4;41(2):202–8.

23. Zheng W, Chen A, Yang H, Hong L. MicroRNA 27a inhibits trophoblast cell migration and invasion by targeting SMAD2: Potential role in preeclampsia. Exp Ther Med. 2020 Jun 24;

24. Sheng C, Zhao Y, Zhu L. Down-regulation of EDN1 gene expression by circulating miR-206 is associated with risk of preeclampsia. Medicine. 2020 May 29;99(22):e20319.

25. Li Q, Han Y, Xu P, Yin L, Si Y, Zhang C, et al. Elevated microRNA-125b inhibits cytotrophoblast invasion and impairs endothelial cell function in preeclampsia. Cell Death Discov. 2020 May 13;6(1):35.

26. Suo M, Sun Y, Yang H, Ji J, He Y, Dong L, et al. miR-183-5p suppressed the invasion and migration of HTR-8/SVneo trophoblast cells partly via targeting MMP-9 in preeclampsia. Biosci Rep. 2020 Jun 26;40(6).

27. Yang X, Meng T. miR‐215‐5p decreases migration and invasion of trophoblast cells through regulating CDC6 in preeclampsia. Cell Biochem Funct. 2020 Jun 23;38(4):472–9.

28. Lv Y, Lu X, Li C, Fan Y, Ji X, Long W, et al. miR-145–5p promotes trophoblast cell growth and invasion by targeting FLT1. Life Sci. 2019 Dec;239:117008.

29. Wang H, Zhao Y, Luo R, Bian X, Wang Y, Shao X, et al. A positive feedback self-regulatory loop between miR-210 and HIF-1α mediated by CPEB2 is involved in trophoblast syncytiolization: implication of trophoblast malfunction in preeclampsia. Biol Reprod. 2019 Oct 16;

30. Dong K, Zhang X, Ma L, Gao N, Tang H, Jian F, et al. Downregulations of circulating miR-31 and miR-21 are associated with preeclampsia. Pregnancy Hypertens. 2019 Jul;17:59–63.

31. Tang Q, Gui J, Wu X, Wu W. Downregulation of miR-424 in placenta is associated with severe preeclampsia. Pregnancy Hypertens. 2019 Jul;17:109–12.

32. Xie N, Jia Z, Li L. miR 320a upregulation contributes to the development of preeclampsia by inhibiting the growth and invasion of trophoblast cells by targeting interleukin 4. Mol Med Rep. 2019 Aug 8;

33. Zhong Y, Zhu F, Ding Y. Differential microRNA expression profile in the plasma of preeclampsia and normal pregnancies. Exp Ther Med. 2019 Jun 3;

34. Wang R, Liu W, Liu X, Liu X, Tao H, Wu D, et al. MicroRNA‐210 regulates human trophoblast cell line HTR‐8/SVneo function by attenuating Notch1 expression: Implications for the role of microRNA‐210 in pre‐eclampsia. Mol Reprod Dev. 2019 Jul 21;86(7):896–907.

35. Yang H li, Zhang H zhi, Meng F rong, Han S yi, Zhang M. Differential expression of microRNA-411 and 376c is associated with hypertension in pregnancy. Brazilian Journal of Medical and Biological Research. 2019;52(4).

36. Liu E, Liu Z, Zhou Y, Chen M, Wang L, Li J. MicroRNA 142 3p inhibits trophoblast cell migration and invasion by disrupting the TGF β1/Smad3 signaling pathway. Mol Med Rep. 2019 Mar 1;

37. Shi Z, Liu B, Li Y, Liu F, Yuan X, Wang Y. MicroRNA‐652‐3p promotes the proliferation and invasion of the trophoblast HTR‐8/SVneo cell line by targeting homeobox A9 to modulate the expression of ephrin receptor B4. Clin Exp Pharmacol Physiol. 2019 Jun;46(6):587–96.

38. Chen J, Zhao L, Wang D, Xu Y, Gao H, Tan W, et al. Contribution of regulatory T□cells to immune tolerance and

association of microRNA 210 and Foxp3 in preeclampsia. Mol Med Rep. 2018 Dec 10;

39. Fu JY, Xiao YP, Ren CL, Guo YW, Qu DH, Zhang JH, et al. Up-regulation of miR-517-5p inhibits ERK/MMP-2 pathway: potential role in preeclampsia. Eur Rev Med Pharmacol Sci. 2018 Oct;22(20):6599–608.

40. Fang YN, Huang ZL, Li H, Tan WB, Zhang QG, Wang L, et al. Highly expressed miR-182-5p can promote preeclampsia progression by degrading RND3 and inhibiting HTR-8/SVneo cell invasion. Eur Rev Med Pharmacol Sci. 2018 Oct;22(20):6583–90.

41. Huang X, Wu L, Zhang G, Tang R, Zhou X. Elevated MicroRNA-181a-5p Contributes to Trophoblast Dysfunction and Preeclampsia. Reproductive Sciences. 2019 Aug 30;26(8):1121–9.

42. Wang N, Li R, Xue M. Potential regulatory network in the PSG10P/miR-19a-3p/IL1RAP pathway is possibly involved in preeclampsia pathogenesis. J Cell Mol Med. 2019 Feb;23(2):852–64.

43. Li J, Du J, Wang Z, Wang C, Bai J, Zhang S. Expression of miR 376 in blood of pregnant women with preeclampsia and its effect on 25 hydroxyvitamin D. Exp Ther Med. 2018 Jul 3;

44. Wang F, Yan J. MicroRNA-454 is involved in regulating trophoblast cell proliferation, apoptosis, and invasion in preeclampsia by modulating the expression of ephrin receptor B4. Biomedicine & Pharmacotherapy. 2018 Nov;107:746–53.

45. Liu F, Wu K, Wu W, Chen Y, Wu H, Wang H, et al. miR 203 contributes to pre eclampsia via inhibition of VEGFA expression. Mol Med Rep. 2018 Feb 2;

46. Niu Z ru, Han T, Sun X luan, Luan L xia, Gou W li, Zhu X ming. MicroRNA-30a-3p is overexpressed in the placentas of patients with preeclampsia and affects trophoblast invasion and apoptosis by its effects on IGF-1. Am J Obstet Gynecol. 2018 Feb;218(2):249.e1-249.e12.

47. Wang S, Wang X, Weng Z, Zhang S, Ning H, Li B. Expression and role of microRNA 18b and hypoxia inducible factor 1α in placental tissues of preeclampsia patients. Exp Ther Med. 2017 Aug 30;

48. Jin M, Li H, Xu H, Huo G, Yao Y. MicroRNA-20b inhibits trophoblast cell migration and invasion by targeting MMP-2. Int J Clin Exp Pathol. 2017;10(11):10901–9.

49. Guo M, Zhao X, Yuan X, Li P. Elevated microRNA-34a contributes to trophoblast cell apoptosis in preeclampsia by targeting BCL-2. J Hum Hypertens. 2017 Dec 12;31(12):815–20.

50. Xiao J, Tao T, Yin Y, Zhao L, Yang L, Hu L. miR-144 may regulate the proliferation, migration and invasion of trophoblastic cells through targeting PTEN in preeclampsia. Biomedicine & Pharmacotherapy. 2017 Oct;94:341–53.

51. Gan L, Liu Z, Wei M, Chen Y, Yang X, Chen L, et al. MiR-210 and miR-155 as potential diagnostic markers for pre-eclampsia pregnancies. Medicine. 2017 Jul;96(28):e7515.

52. Fang M, Du H, Han B, Xia G, Shi X, Zhang F, et al. Hypoxia-inducible microRNA-218 inhibits trophoblast invasion by targeting LASP1: Implications for preeclampsia development. Int J Biochem Cell Biol. 2017 Jun;87:95–103.

53. Shao X, Liu Y, Liu M, Wang Y, Yan L, Wang H, et al. Testosterone Represses Estrogen Signaling by Upregulating miR-22. Hypertension. 2017 Apr;69(4):721–30.

54. Yang W, Wang A, Zhao C, Li Q, Pan Z, Han X, et al. miR-125b Enhances IL-8 Production in Early-Onset Severe Preeclampsia by Targeting Sphingosine-1-Phosphate Lyase 1. PLoS One. 2016 Dec 9;11(12):e0166940.

55. Zhou X, Li Q, Xu J, Zhang X, Zhang H, Xiang Y, et al. The aberrantly expressed miR-193b-3p contributes to preeclampsia through regulating transforming growth factor-β signaling. Sci Rep. 2016 Jan 29;6(1):19910.

56. Wang Y, Yang X, Yang Y, Wang W, Zhao M, Liu H, et al. High-throughput deep screening and identification of four peripheral leucocyte microRNAs as novel potential combination biomarkers for preeclampsia. Journal of Perinatology. 2016 Apr 17;36(4):263–7.

57. Li Q, Long A, Jiang L, Cai L, Xie L, Gu J, et al. Quantification of preeclampsia-related microRNAs in maternal serum. Biomed Rep. 2015 Nov;3(6):792–6.

58. Sun M, Chen H, Liu J, Tong C, Meng T. MicroRNA-34a inhibits human trophoblast cell invasion by targeting MYC. BMC Cell Biol. 2015 Dec 3;16(1):21.

59. Ding J, Huang F, Wu G, Han T, Xu F, Weng D, et al. MiR-519d-3p Suppresses Invasion and Migration of Trophoblast Cells via Targeting MMP-2. PLoS One. 2015 Mar 24;10(3):e0120321.

60. Zhang C, Li Q, Ren N, Li C, Wang X, Xie M, et al. Placental miR-106a~363 cluster is dysregulated in preeclamptic placenta. Placenta. 2015 Feb;36(2):250–2.

61. Hong F, Li Y, Xu Y. Decreased placental miR-126 expression and vascular endothelial growth factor levels in patients with pre-eclampsia. Journal of International Medical Research. 2014 Dec 23;42(6):1243–51.

62. Li Q, Pan Z, Wang X, Gao Z, Ren C, Yang W. miR-125b-1-3p inhibits trophoblast cell invasion by targeting sphingosine-1-phosphate receptor 1 in preeclampsia. Biochem Biophys Res Commun. 2014 Oct;453(1):57–63.

63. Li X, Li C, Dong X, Gou W. MicroRNA-155 inhibits migration of trophoblast cells and contributes to the pathogenesis of severe preeclampsia by regulating endothelial nitric oxide synthase. Mol Med Rep. 2014 Jul;10(1):550–4.

64. Xu P, Zhao Y, Liu M, Wang Y, Wang H, Li Y xia, et al. Variations of MicroRNAs in Human Placentas and Plasma From Preeclamptic Pregnancy. Hypertension. 2014 Jun;63(6):1276–84.

65. Li H, Ge Q, Guo L, Lu Z. Maternal Plasma miRNAs Expression in Preeclamptic Pregnancies. Biomed Res Int. 2013;2013:1–9.

66. Wu L, Zhou H, Lin H, Qi J, Zhu C, Gao Z, et al. Circulating microRNAs are elevated in plasma from severe preeclamptic pregnancies. REPRODUCTION. 2012 Mar;143(3):389–97.

67. Zhang Y, Fei M, Xue G, Zhou Q, Jia Y, Li L, et al. Elevated levels of hypoxia-inducible microRNA-210 in pre-eclampsia: new insights into molecular mechanisms for the disease. J Cell Mol Med. 2012 Feb;16(2):249–59.

68. Hu Y, Li P, Hao S, Liu L, Zhao J, Hou Y. Differential expression of microRNAs in the placentae of Chinese patients with severe pre-eclampsia. Clin Chem Lab Med. 2009 Jan 1;47(8).

69. Zhu X ming, Han T, Sargent IL, Yin G wu, Yao Y qing. Differential expression profile of microRNAs in human placentas from preeclamptic pregnancies vs normal pregnancies. Am J Obstet Gynecol. 2009 Jun;200(6):661.e1-661.e7.

70. Jin Y, Jia T, Wu X, Wang Y, Sun W, Chen Y, et al. The predictive value of microRNA in early hypertensive disorder complicating pregnancy (HDCP). Am J Transl Res. 2021;13(6):7288–93.

71. Fan Z, Wang Q, Deng H. Circ_0011460 upregulates HTRA1 expression by sponging miR‐762 to suppress HTR8/SVneo cell growth, migration, and invasion. American Journal of Reproductive Immunology. 2021 Nov 26;86(5).

72. Chu X, Gu Y, Sheng W, Sun J, Morgan JA, Lewis DF, et al. Downregulation of miR-126-3p expression contributes to increased inflammatory response in placental trophoblasts in preeclampsia. J Reprod Immunol. 2021 Apr;144:103281.

73. Yu Y, An X, Fan D. Histone Deacetylase Sirtuin 2 Enhances Viability of Trophoblasts Through p65-Mediated MicroRNA-146a/ACKR2 Axis. Reproductive Sciences. 2021 May 6;28(5):1370–81.

74. Chen J, Zhan Y, Xu J, Wang Y, Gao Q. EGR1 Overexpression Inhibits the Occurrence of Preeclampsia by Binding to MicroRNA-574 Promoter and Upregulating GAB1. Reproductive Sciences. 2021 Apr 19;28(4):1112–21.

75. Ni H, Wang X, Qu H, Gao X, Yu X. MiR-95-5p involves in the migration and invasion of trophoblast cells by targeting low density lipoprotein receptor-related protein 6. J Obstet Gynaecol Res. 2021 Jan;47(1):184–97.

76. Mi C, Ye B, Gao Z, Du J, Li R, Huang D. BHLHE40 plays a pathological role in pre-eclampsia through upregulating SNX16 by transcriptional inhibition of miR-196a-5p. Mol Hum Reprod. 2020 Jul 1;26(7):532–48.

77. Zhang L, Li H, Li M, Zhang W, Yang Z, Zhang S. LRP6 is involved in the proliferation, migration and invasion of trophoblast cells via miR 346. Int J Mol Med. 2020 Apr 8;

78. Liu R, Wang X, Yan Q. The regulatory network of lncRNA DLX6-AS1/miR-149–5p/ERP44 is possibly related to

the progression of preeclampsia. Placenta. 2020 Apr;93:34–42.

79. Qian S, Liu R. miR-30b facilitates preeclampsia through targeting MXRA5 to inhibit the viability, invasion and apoptosis of placental trophoblast cells. Int J Clin Exp Pathol. 2019;12(11):4057–65.

80. Cao G, Cui R, Liu C, Zhang Z. MicroRNA regulation of transthyretin in trophoblast biofunction and preeclampsia. Arch Biochem Biophys. 2019 Nov;676:108129.

81. Yang X, Guo F. miR 342 3p suppresses cell migration and invasion in preeclampsia by targeting platelet derived growth factor receptor α. Mol Med Rep. 2019 Jun 10;

82. Xue F, Yang J, Li Q, Zhou H. Down-regulation of microRNA-34a-5p promotes trophoblast cell migration and invasion via targetting Smad4. Biosci Rep. 2019 Feb 28;39(2).

83. Chi Z, Zhang M. Exploration of the regulation and control mechanisms of miR 145 in trophoblast cell proliferation and invasion. Exp Ther Med. 2018 Oct 23;

84. Shi Z, She K, Li H, Yuan X, Han X, Wang Y. MicroRNA-454 contributes to sustaining the proliferation and invasion of trophoblast cells through inhibiting Nodal/ALK7 signaling in pre-eclampsia. Chem Biol Interact. 2019 Jan;298:8–14.

85. Yang X, Meng T. MicroRNA-431 affects trophoblast migration and invasion by targeting ZEB1 in preeclampsia. Gene. 2019 Jan;683:225–32.

86. Wu L, Song W yan, Xie Y, Hu L li, Hou X man, Wang R, et al. miR-181a-5p suppresses invasion and migration of HTR-8/SVneo cells by directly targeting IGF2BP2. Cell Death Dis. 2018 Jan 16;9(2):16.

87. Gao Y, She R, Wang Q, Li Y, Zhang H. Up-regulation of miR-299 suppressed the invasion and migration of HTR-8/SVneo trophoblast cells partly via targeting HDAC2 in pre-eclampsia. Biomedicine & Pharmacotherapy. 2018 Jan;97:1222–8.

88. Yang X, Zhang J, Ding Y. Association of microRNA-155, interleukin 17A, and proteinuria in preeclampsia. Medicine. 2017 May;96(18):e6509.

89. Jiang L, Long A, Tan L, Hong M, Wu J, Cai L, et al. Elevated microRNA-520g in pre-eclampsia inhibits migration and invasion of trophoblasts. Placenta. 2017 Mar;51:70–5.

90. Xu Y, Huang X, Xie J, Chen Y, Fu J, Wang L. Let-7i-Induced Atg4B Suppression Is Essential for Autophagy of Placental Trophoblast in Preeclampsia. J Cell Physiol. 2017 Sep;232(9):2581–9.

91. Lu TM, Lu W, Zhao LJ. MicroRNA-137 Affects Proliferation and Migration of Placenta Trophoblast Cells in Preeclampsia by Targeting ERRα. Reproductive Sciences. 2017 Jan 1;24(1):85–96.

92. Hu TX, Wang G, Guo XJ, Sun QQ, He P, Gu H, et al. MiR 20a,-20b and -200c are involved in hydrogen sulfide stimulation of VEGF production in human placental trophoblasts. Placenta. 2016 Mar;39:101–10.

93. Jiang F, Li J, Wu G, Miao Z, Lu L, Ren G, et al. Upregulation of microRNA-335 and microRNA-584 contributes to the pathogenesis of severe preeclampsia through downregulation of endothelial nitric oxide synthase. Mol Med Rep. 2015 Oct;12(4):5383–90.

94. Luo R, Shao X, Xu P, Liu Y, Wang Y, Zhao Y, et al. MicroRNA-210 Contributes to Preeclampsia by Downregulating Potassium Channel Modulatory Factor 1. Hypertension. 2014 Oct;64(4):839–45.

95. Zhang Y, Diao Z, Su L, Sun H, Li R, Cui H, et al. MicroRNA-155 contributes to preeclampsia by down-regulating CYR61. Am J Obstet Gynecol. 2010 May;202(5):466.e1-466.e7.

96. Du M, Wu S, Chen J, Liu X, Yu P, Wang L. Diagnostic Value and Clinical Significance of MicroRNA-193b-5p in Hypertensive Disorder Complicating Pregnancy. Indian J Pharm Sci. 2022;84(S2).

97. Lei D, Fang C, Deng N, Yao B, Fan C. Long noncoding RNA expression profiling identifies MIR210HG as a novel molecule in severe preeclampsia. Life Sci. 2021 Apr;270:119121.

98. Luo S, Wang L, Li S, Wang H, Huang S, Zhang Z, et al. Identification of Key Molecules and lncRNA-miRNA-

mRNA ceRNA Network in Preeclampsia. Int J Gen Med. 2021 Nov;Volume 14:7579–90.

99. Zhou Q, Li H, Zhang Y, Peng W, Hou H, Gu M, et al. MicroRNA-513c-5p is involved in the pathogenesis of preeclampsia by regulating of low-density lipoprotein receptor-associated protein 6. BMC Pregnancy Childbirth. 2021 Dec 20;21(1):837.

100. Cai H, Li D, Wu J, Shi C. miR-519d downregulates LEP expression to inhibit preeclampsia development. Open Medicine. 2021 Aug 25;16(1):1215–27.

101. Yang Y, Tang F, Zhao X. miR-27b-3p is Highly Expressed in Serum of Patients with Preeclampsia and has Clinical Significance. Endocr Metab Immune Disord Drug Targets. 2022 May;22(6):612–9.

102. Yang HY. MiR-133b regulates oxidative stress injury of trophoblasts in preeclampsia by mediating the JAK2/STAT3 signaling pathway. J Mol Histol. 2021 Dec 8;52(6):1177–88.

103. Wu M, Zhao Y, Li L, Wang G, Xing L. Exosomal microRNA 302a promotes trophoblast migration and proliferation, and represses angiogenesis by regulating the expression levels of VEGFA in preeclampsia. Mol Med Rep. 2021 Oct 19;24(6):864.

104. Mao Q, Zou H. Circular RNA circ_0032962 promotes trophoblast cell progression as ceRNA to target PBX3 via sponging miR-326 in preeclampsia. Reprod Biol. 2021 Dec;21(4):100571.

105. Jin M, Xu S, Li J, Yao Y, Tang C. MicroRNA-3935 promotes human trophoblast cell epithelial-mesenchymal transition through tumor necrosis factor receptor-associated factor 6/regulator of G protein signaling 2 axis. Reproductive Biology and Endocrinology. 2021 Dec 7;19(1):134.

106. Dong N, Li D, Cai H, Shi L, Huang L. Expression of lncRNA MIR193BHG in serum of preeclampsia patients and its clinical significance. J Gynecol Obstet Hum Reprod. 2022 May;51(5):102357.

107. Li Y, Chen J, Song S. Circ‐OPHN1 suppresses the proliferation, migration, and invasion of trophoblast cells through mediating miR‐558/THBS2 axis. Drug Dev Res. 2022 Jun 11;83(4):1034–46.

108. Li H, Zhou L, Zhang C, Xi Q, Lv J, Huo W, et al. Follistatin dysregulation impaired trophoblast biological functions by GDF11-Smad2/3 axis in preeclampsia placentas. Placenta. 2022 Apr;121:145–54.

109. Wang W, Shi J, Zheng L. Identification of Circular RNA circ_0017068 as a Regulator of Proliferation and Apoptosis in Trophoblast Cells by miR-330-5p/XIAP Axis. Reproductive Sciences. 2022 Aug 3;29(8):2414–27.

110. Zhang Z, Wang Y. Over-expression of long non-coding RNA NORAD promotes trophoblastic cell viability, migration, and invasion in preeclampsia via the miR-202-5p/FXR1 axis. Taiwan J Obstet Gynecol. 2022 Mar;61(2):255–64.

111. Xing H, Ding Q, Lu H, Li Q. Circ_0007611 stimulates IL-1 receptor accessory protein to inhibit trophoblast cell proliferation and induce cell apoptosis. Biol Reprod. 2022 May 17;106(5):1011–21.

112. Liu J, Yang Y, Liu W, Lan R. circ_0085296 inhibits the biological functions of trophoblast cells to promote the progression of preeclampsia via the miR-942-5p/THBS2 network. Open Medicine. 2022 Mar 21;17(1):577–88.

113. Yang Z, Shan N, Deng Q, Wang Y, Hou Y, Mei J, et al. Extracellular vesicle‐derived microRNA‐18b ameliorates preeclampsia by enhancing trophoblast proliferation and migration via Notch2/TIM3/mTORC1 axis. J Cell Mol Med. 2021 May 9;25(10):4583–95.

114. Liu Y, Ma X, Liu Y. Hsa_circ_0001326 regulates proliferation, migration, invasion, and EMT of HTR‐8/SVneo cells via increasing IL16 expression. American Journal of Reproductive Immunology. 2021 Nov 22;86(5).

115. Yuan Y, Gong Y, Zhong L, Ding X, Yang Z, Su X, et al. Circular RNA expression profile and competing endogenous RNA regulatory network in preeclampsia. Placenta. 2022 Mar;119:32–8.

116. Wang W, Liu J, Pan E. CircHIPK3 contributes to human villous trophoblast growth, migration and invasion via modulating the pathway of miR-346/KCMF1. Placenta. 2022 Feb;118:46–54.

117. Jiang Y, Luo T, Xia Q, Tian J, Yang J. microRNA-140-5p from human umbilical cord mesenchymal stem cells–

released exosomes suppresses preeclampsia development. Funct Integr Genomics. 2022 Oct 28;22(5):813–24.

118. Li C, Li Q. Circular RNA circ_0111277 Serves as ceRNA, Targeting the miR-424-5p/NFAT5 Axis to Regulate the Proliferation, Migration, and Invasion of Trophoblast Cells in Preeclampsia. Reproductive Sciences. 2022 Mar 30;29(3):923–35.

119. Wei X, Yuan Y, Yang Q. SNHG22 promotes migration and invasion of trophoblasts via miR-128-3p/PCDH11X axis and activates PI3K/Akt signaling pathway. Clinics. 2022 Jan;77:100055.

120. Bai Y, Yang W, Yang H xia, Liao Q, Ye G, Fu G, et al. Downregulated miR-195 Detected in Preeclamptic Placenta Affects Trophoblast Cell Invasion via Modulating ActRIIA Expression. PLoS One. 2012 Jun 19;7(6):e38875.

121. Zou Y, Jiang Z, Yu X, Zhang Y, Sun M, Wang W, et al. MiR-101 regulates apoptosis of trophoblast HTR-8/SVneo cells by targeting endoplasmic reticulum (ER) protein 44 during preeclampsia. J Hum Hypertens. 2014 Oct 1;28(10):610–6.

122. Zhang Y, Huang G, Zhang Y, Yang H, Long Y, Liang Q, et al. MiR-942 decreased before 20 weeks gestation in women with preeclampsia and was associated with the pathophysiology of preeclampsia in vitro. Clin Exp Hypertens. 2017 Feb 17;39(2):108–13.

123. Wang H, Zhang L, Guo X, Bai Y, Li YX, Sha J, et al. MiR-195 modulates oxidative stress-induced apoptosis and mitochondrial energy production in human trophoblasts via flavin adenine dinucleotide-dependent oxidoreductase domain-containing protein 1 and pyruvate dehydrogenase phosphatase regulatory subunit. J Hypertens. 2018 Feb;36(2):306–18.

124. Ma R, Lu Y, Dou C, Gu Q. Clinical significance of miR-133a and miR-206 in pregnant women with preeclampsia and correlation with pregnancy outcomes. Int J Clin Exp Med. 2019;12(9):7383–91.

125. Wang Y, Cheng K, Zhou W, Liu H, Yang T, Hou P, et al. miR-141-5p regulate ATF2 via effecting MAPK1/ERK2 signaling to promote preeclampsia. Biomedicine & Pharmacotherapy. 2019 Jul;115:108953.

126. Yang Y, Li H, Ma Y, Zhu X, Zhang S, Li J. MiR-221-3p is down-regulated in preeclampsia and affects trophoblast growth, invasion and migration partly via targeting thrombospondin 2. Biomedicine & Pharmacotherapy. 2019 Jan;109:127–34.

127. Yang Y, Xi L, Ma Y, Zhu X, Chen R, Luan L, et al. The lncRNA small nucleolar RNA host gene 5 regulates trophoblast cell proliferation, invasion, and migration via modulating miR‐26a‐5p/N‐cadherin axis. J Cell Biochem. 2019 Mar 22;120(3):3173–84.

128. Huang J, Zheng L, Kong H, Wang F, Su Y, Xin H. miR-139-5p promotes the proliferation and invasion of trophoblast cells by targeting sFlt-1 in preeclampsia. Placenta. 2020 Mar;92:37–43.

129. Li T, Zhou B, He Y, Liu J, Li Y. Expression and clinical diagnostic value of miR-383 in patients with severe preeclampsia. Cell Mol Biol. 2020 Jun 5;66(3):92–100.

130. Li HQ, Fan JJ, Li XH, Bao D. MiR-507 inhibits the growth and invasion of trophoblasts by targeting CAMK4. Eur Rev Med Pharmacol Sci. 2020 Jun;24(11):5856–62.

131. Wu D, Shi L, Hong L, Chen X, Cen H. MiR-135a-5p promotes the migration and invasion of trophoblast cells in preeclampsia by targeting β-TrCP. Placenta. 2020 Sep;99:63–9.

132. Yu Z, Zhang Y, Zheng H, Gao Q, Wang H. LncRNA SNHG16 regulates trophoblast functions by the miR-218-5p/LASP1 axis. J Mol Histol. 2021 Oct 10;52(5):1021–33.

133. Xu B, Geng X, Liu X, Liu Y. Long non-coding RNA FAM99A modulated YAP1 to affect trophoblast cell behaviors in preeclampsia by sponging miR-134-5p. Brazilian Journal of Medical and Biological Research. 2020;53(12).

134. Cao C, Cui J, Liu G. circ_0004904 regulates the trophoblast cell in preeclampsia via miR-19b-3p/ARRDC3 axis. Open Medicine. 2023 May 16;18(1).

135. Chen C, Gao J, Chen D, Liu J, He B, Chen Y, et al. miR-4443/MMP2 suppresses the migration and invasion of trophoblasts through the HB-EGF/EGFR pathway in preeclampsia. Cell Cycle. 2022 Dec 2;21(23):2517–32.

136. Chen Y, Zhou C, Zhao X, Che R, Wu Y, Wan S, et al. Extracellular Vesicles Derived from Human Umbilical Cord Mesenchymal Stem Cells Promote Trophoblast Cell Proliferation and Migration by Targeting TFPI2 in Preeclampsia. Stem Cells Int. 2023 Aug 1;2023:1–10.

137. Gu F, Lu D, Zhang L. MicroRNA-30a contributes to pre-eclampsia through regulating the proliferation, apoptosis, and angiogenesis modulation potential of mesenchymal stem cells by targeting AVEN. Bioengineered. 2022 Apr 1;13(4):8724–34.

138. Hu M, Zheng Y, Liao J, Wen L, Cheng J, Huang J, et al. miR21 modulates the Hippo signaling pathway via interference with PP2A Bβ to inhibit trophoblast invasion and cause preeclampsia. Mol Ther Nucleic Acids. 2022 Dec;30:143–61.

139. Jiang L ling, Yang D lin, Han Q, Zhang H le, Pan M, Yan J ying. LncRNA-NEAT1 blocks the Wnt/β-catenin signaling pathway by targeting miR-217 to inhibit trophoblast cell migration and invasion. J Assist Reprod Genet. 2024 Aug 6;41(8):2107–15.

140. Li K, Lv C, Zhang W, Fang J. CircFN1 upregulation initiated oxidative stress-induced apoptosis and inhibition of proliferation and migration in trophoblasts via circFN1-miR-19a/b-3p-ATF2 ceRNA network. Reprod Biol. 2022 Jun;22(2):100631.

141. Li Z, Ru X, Wang S, Cao G. miR‐24‐3p regulation of retinol binding protein 4 in trophoblast biofunction and preeclampsia. Mol Reprod Dev. 2022 Sep 12;89(9):423–30.

142. Li Z, Wang J, Li D, Chen H, Meng T. miR 372 3p promotes preeclampsia progression by regulating twist1. Exp Ther Med. 2022 Oct 19;24(6):723.

143. Liao W, Zeng H, Jiang X, Deng X, Tu S, Lan H, et al. CircPAPPA2 plays a role in preeclampsia pathogenesis via regulation of the miR-942/miR-5006-3p. BMC Pregnancy Childbirth. 2024 Jun 7;24(1):414.

144. LIN L, LIAN X, LIU Y, LIN A. MicroRNA-574-5p affects trophoblast proliferation, migration and invasion by targeting ZEB1 in preeclampsia. Panminerva Med. 2022 Oct;64(3).

145. Liu X, Li Z, Lu D. MicroRNA-223-3p downregulates the inflammatory response in preeclampsia placenta via targeting NLRP3. BMC Pregnancy Childbirth. 2024 Mar 6;24(1):175.

146. Liu H, Wang X. MiR-200b-3p is upregulated in the placental tissues from patients with preeclampsia and promotes the development of preeclampsia via targeting profilin 2. Cell Cycle. 2022 Sep 17;21(18):1945–57.

147. Lu C, Zheng F, Pan L, Han Q, Wu J, Zhang W. Suppression of circular <scp>RNA</scp> serum and glucocorticoid‐induced kinase 1 elevates antioxidant molecules and angiogenesis in trophoblast cells to attenuate preeclampsia via <scp>microRNA</scp> ‐508‐3p to target and restrain <scp>PUM</scp> homolog 1. Journal of Obstetrics and Gynaecology Research. 2024 Mar 11;50(3):322–33.

148. MO W, JIN J, WANG X, LUAN W, YAN J, LONG X. MicroRNA-206 Contributes to the Progression of Preeclampsia by Suppressing the Viability and Mobility of Trophocytes via the Inhibition of AGTR1. Physiol Res. 2023 Nov 20;72(5):597–606.

149. Ning W, Chen Y, Chen Y, Zhang H, Wu B, Wen C. Correlation and predictive value of serum miR-146b-5p expression during the first trimester of pregnancy with pre-eclampsia. J Obstet Gynaecol (Lahore). 2022 Nov 17;42(8):3537–44.

150. PENG X, ZHANG R, ZHANG Y, CAI C. Nuclear Factor-Kappa B-induced miRNA-518a-5p represses trophoblast cell migration and invasion by the Nuclear Factor-Kappa B pathway. An Acad Bras Cienc. 2023;95(1).

151. Shang J, Lin L, Huang X, Zhou L, Huang Q. Re-expression of circ_0043610 contributes to trophoblast dysfunction through the miR-558/RYBP pathway in preeclampsia. Endocr J. 2022;69(12):EJ22-0153.

152. Su S, Zhong L, Huang S, Deng L, Pang L. MiRNA-494 induces trophoblast senescence by targeting SIRT1. Hypertens Pregnancy. 2023 Dec 31;42(1).

153. Sui S, Zhang Y, Huang Y. microRNA-378a-3p plays a regulatory role in trophoblast cell function in preeclampsia by targeting CMTM3. Mol Cell Endocrinol. 2023 Oct;576:111997.

154. Tian X, Zhang Y, Zhao M, Yin X. Circ_0030042 inhibits trophoblast cell growth, invasion and epithelial-mesenchymal transition process in preeclampsia via miR-942-5p/LITAF. J Reprod Immunol. 2024 Mar;162:104205.

155. Wang Z, Liu D, Dai Y, Li R, Zheng Y, Zhao G, et al. Elevated Placental microRNA-155 Is a Biomarker of a Preeclamptic Subtype. Hypertension. 2023 Feb;80(2):370–84.

156. Wang L, Shi L, Zhou B, Hong L, Gong H, Wu D. METTL3-mediated lncRNA HOXD-AS1 stability regulates inflammation, and the migration and invasion of trophoblast cells via the miR-135a/ β-TRCP axis. Noncoding RNA Res. 2024 Mar;9(1):12–23.

157. Wei X, Yuan Y, Yang Q. Long noncoding RNA PVT1 accelerates the growth of placental trophoblasts in preeclampsia through the microRNA‐24‐3p/HSD11B2 axis. Mol Reprod Dev. 2022 Jul 23;89(7):271–80.

158. Xu J, Wang J, Chen M, Chao B, He J, Bai Y, et al. miR-101-5p suppresses trophoblast cell migration and invasion via modulating the DUSP6-ERK1/2 axis in preeclampsia. J Assist Reprod Genet. 2023 Jul 10;40(7):1597–610.

159. Xu X, Teng H. circRNA circ_0055724 Inhibits Trophoblastic Cell Line HTR-8/SVneo's Invasive and Migratory Abilities via the miR-136/N-Cadherin Axis. Dis Markers. 2022 Jun 22;2022:1–13.

160. Zhou D, Qu B, Zhang X. Diagnostic value of serum miR-25-3p in hypertensive disorders in pregnancy. Women Health. 2022 Nov 26;62(9–10):818–26.

161. Zhou D, Xu X, Liu Y, Liu H, Cheng X, Gu Y, et al. MiR‐195‐5p facilitates the proliferation, migration, and invasion of human trophoblast cells by targeting FGF2. Journal of Obstetrics and Gynaecology Research. 2022 Aug 18;48(8):2122–33.

162. Zhou M, Yu X, Li C, Lou L, Yang S, Cai J, et al. Circ_0111277 suppresses trophoblast cell proliferation, angiogenesis, migration, invasion and EMT via regulating miR‐188‐3p/GRHL2 axis. American Journal of Reproductive Immunology. 2023 Mar 29;89(3).

163. Zhu D, Guo T, Xu J, Yuan D, Lin M, Yang M. Elevated Expression of miR-296 in Human Placentas and Serum Samples From Pregnancies With Preeclampsia. Br J Biomed Sci. 2023 Apr 11;80.

164. Zhu W, Chen X. miR 424 5p is downregulated in the placentas of patients with preeclampsia and affects trophoblast migration and invasion. Exp Ther Med. 2023 May 5;25(6):294.

165. Leseva MN, Binder AM, Ponsonby AL, Vuillermin P, Saffery R, Michels KB. Differential gene expression and limited epigenetic dysregulation at the materno-fetal interface in preeclampsia. Hum Mol Genet. 2020 Jan 15;29(2):335–50.

166. Singh K, Williams J, Brown J, Wang ET, Lee B, Gonzalez TL, et al. Up-regulation of microRNA-202-3p in first trimester placenta of pregnancies destined to develop severe preeclampsia, a pilot study. Pregnancy Hypertens. 2017 Oct;10:7–9.

167. Anton L, Olarerin-George AO, Hogenesch JB, Elovitz MA. Placental Expression of miR-517a/b and miR-517c Contributes to Trophoblast Dysfunction and Preeclampsia. PLoS One. 2015 Mar 23;10(3):e0122707.

168. Anton L, Olarerin-George AO, Schwartz N, Srinivas S, Bastek J, Hogenesch JB, et al. miR-210 Inhibits Trophoblast Invasion and Is a Serum Biomarker for Preeclampsia. Am J Pathol. 2013 Nov;183(5):1437–45.

169. Betoni JS, Derr K, Pahl MC, Rogers L, Muller CL, Packard RE, et al. MicroRNA analysis in placentas from patients with preeclampsia: comparison of new and published results. Hypertens Pregnancy. 2013 Nov 11;32(4):321–39.

170. Guo L, Tsai SQ, Hardison NE, James AH, Motsinger-Reif AA, Thames B, et al. Differentially expressed microRNAs and affected biological pathways revealed by modulated modularity clustering (MMC) analysis of human preeclamptic and IUGR placentas. Placenta. 2013 Jul;34(7):599–605.

171. Kumar P, Luo Y, Tudela C, Alexander JM, Mendelson CR. The c-Myc-Regulated MicroRNA-17~92 (miR-17~92) and miR-106a~363 Clusters Target hCYP19A1 and hGCM1 To Inhibit Human Trophoblast Differentiation. Mol Cell Biol. 2013 May 1;33(9):1782–96.

172. Wang W, Feng L, Zhang H, Hachy S, Satohisa S, Laurent LC, et al. Preeclampsia Up-Regulates Angiogenesis-Associated MicroRNA ( i.e ., miR-17, -20a, and -20b) That Target Ephrin-B2 and EPHB4 in Human Placenta. J Clin Endocrinol Metab. 2012 Jun 1;97(6):E1051–9.

173. Enquobahrie DA, Abetew DF, Sorensen TK, Willoughby D, Chidambaram K, Williams MA. Placental microRNA expression in pregnancies complicated by preeclampsia. Am J Obstet Gynecol. 2011 Feb;204(2):178.e12-178.e21.

174. Mayor-Lynn K, Toloubeydokhti T, Cruz AC, Chegini N. Expression Profile of MicroRNAs and mRNAs in Human Placentas From Pregnancies Complicated by Preeclampsia and Preterm Labor. Reproductive Sciences. 2011 Jan 31;18(1):46–56.

175. Pineles BL, Romero R, Montenegro D, Tarca AL, Han YM, Kim YM, et al. Distinct subsets of microRNAs are expressed differentially in the human placentas of patients with preeclampsia. Am J Obstet Gynecol. 2007 Mar;196(3):261.e1-261.e6.

176. Korkes HA, De Oliveira L, Sass N, Salahuddin S, Karumanchi SA, Rajakumar A. Relationship between hypoxia and downstream pathogenic pathways in preeclampsia. Hypertens Pregnancy. 2017 Apr 3;36(2):145–50.

177. Zhang M, Muralimanoharan S, Wortman AC, Mendelson CR. Primate-specific miR-515 family members inhibit key genes in human trophoblast differentiation and are upregulated in preeclampsia. Proceedings of the National Academy of Sciences. 2016 Nov 8;113(45).

178. Brooks SA, Martin E, Smeester L, Grace MR, Boggess K, Fry RC. miRNAs as common regulators of the transforming growth factor (TGF)-β pathway in the preeclamptic placenta and cadmium-treated trophoblasts: Links between the environment, the epigenome and preeclampsia. Food and Chemical Toxicology. 2016 Dec;98:50–7.

179. Demirer S, Hocaoglu M, Bayrak A.E. Determination of the relationship between early preeclampsia and mir518b in maternal blood. Gazi Medical Journal. 2019;30(1):P9.

180. Akgör U, Ayaz L, Çayan F. Expression levels of maternal plasma microRNAs in preeclamptic pregnancies. J Obstet Gynaecol (Lahore). 2021 Aug 18;41(6):910–4.

181. Gunel T, Kamali N, Hosseini MK, Gumusoglu E, Benian A, Aydinli K. Regulatory effect of miR-195 in the placental dysfunction of preeclampsia. The Journal of Maternal-Fetal & Neonatal Medicine. 2018 Mar 18;33(6):901–8.

182. Gunel T, Hosseini MK, Gumusoglu E, Kisakesen HI, Benian A, Aydinli K. Expression profiling of maternal plasma and placenta microRNAs in preeclamptic pregnancies by microarray technology. Placenta. 2017 Apr;52:77–85.

183. Demirer S, Hocaoglu M, Turgut A, Karateke A, Komurcu-Bayrak E. Expression profiles of candidate microRNAs in the peripheral blood leukocytes of patients with early- and late-onset preeclampsia versus normal pregnancies. Pregnancy Hypertens. 2020 Jan;19:239–45.

184. Hocaoglu M, Demirer S, Senturk H, Turgut A, Komurcu-Bayrak E. Differential expression of candidate circulating microRNAs in maternal blood leukocytes of the patients with preeclampsia and gestational diabetes mellitus. Pregnancy Hypertens. 2019 Jul;17:5–11.

185. Simsek F, Turunc E, Keskin-Arslan E, Erol H, Acar S, Atakul BK, et al. Molecular mechanisms involved in pre-eclampsia through expressional regulation of endothelin-1. Placenta. 2022 Jun;124:55–61.

186. Hocaoglu M. Expression Profiles of MIR-155-5P and MIR-518B Micrornas in Circulating Leukocytes of the Pregnant Patients with Preeclampsia and Polycystic Ovary Syndrome. Acta Endocrinologica (Bucharest). 2023;19(4):426–34.

187. Ozler S, Kebapcilar A, Ozdemir EM, Mert M, Arıkan MN, Celik C. Are Vascular Endothelium and Angiogenesis Effective MicroRNA Biomarkers Associated with the Prediction of Early-Onset Preeclampsia (EOPE) and Adverse Perinatal Outcomes? Reproductive Sciences. 2024 Mar 17;31(3):803–10.

188. Abbas AM, Youssef F, Gomaa AM, Kamal DT, Abd Allah ES, Ahmed MA. 418 Role of miRNA-452 expression in the pathogenesis of early onset preeclampsia: a case-control study. Am J Obstet Gynecol. 2021 Feb;224(2):S268–9.

189. Youssef HMG, Marei ES. Association of MicroRNA-210 and MicroRNA-155 with severity of preeclampsia. Pregnancy Hypertens. 2019 Jul;17:49–53.

190. Adel S, Mansour A, Louka M, Matboli M, Elmekkawi SF, Swelam N. Evaluation of MicroRNA-210 and Protein tyrosine phosphatase, non-receptor type 2 in Pre-eclampsia. Gene. 2017 Jan;596:105–9.

191. Abbas MA, Abo Shady HM, Ahmed Elshafey OH, Al-Sheikh NM. Association between expression levels of p53, miRNA-21, and lncRNA-TCL6 and the risk of preeclampsia in pregnant women. Gene. 2024 Jan;893:147932.

192. Ayoub SE, Shaker OG, Aboshama RA, Etman MK, Khalefa AA, khamiss Abd elguaad MM, et al. Expression profile of LncRNA ANRIL, miR-186, miR-181a, and MTMR-3 in patients with preeclampsia. Noncoding RNA Res. 2023 Dec;8(4):481–6.

193. Ellakwa DES, Rashed LA, El-Mandoury AAA, Younis NF. Epigenetic alterations in preeclampsia: a focus on microRNA149 and tetrahydrofolate reductase gene polymorphisms in Egyptian women. Irish Journal of Medical Science (1971 -). 2024 Jun 7;

194. Mamdouh Shoeib S, Elwy Abdeldaim D, Samir Mashal S, Raafat Ibrahim R, Mohamed Dawood L, Shatat D, et al. The Ratio of Cysteine-Rich Angiogenic Inducer 61 to MicroRNA -155 Expression as a Preeclampsia Diagnostic Marker and Predictor of Its Severity. Rep Biochem Mol Biol. 2023 Aug 1;12(2):332–9.

195. Senousy MA, Shaker OG, Elmaasrawy AHZ, Ashour AM, Alsufyani SE, Arab HH, et al. Serum lncRNAs TUG1, H19, and NEAT1 and their target miR-29b/SLC3A1 axis as possible biomarkers of preeclampsia: Potential clinical insights. Noncoding RNA Res. 2024 Dec;9(4):995–1008.

196. Timofeeva A V., Gusar VA, Kan NE, Prozorovskaya KN, Karapetyan AO, Bayev OR, et al. Identification of potential early biomarkers of preeclampsia. Placenta. 2018 Jan;61:61–71.

197. Nizyaeva N V., Kulikova G V., Nagovitsyna MN, Kan NE, Prozorovskaya KN, Shchegolev AI, et al. Expression of MicroRNA-146a and MicroRNA-155 in Placental Villi in Early- and Late-Onset Preeclampsia. Bull Exp Biol Med. 2017 Jul 27;163(3):394–9.

198. Akehurst C, Small HY, Sharafetdinova L, Forrest R, Beattie W, Brown CE, et al. Differential expression of microRNA-206 and its target genes in preeclampsia. J Hypertens. 2015 Oct;33(10):2068–74.

199. Gusar V, Timofeeva A, Chagovets V, Kan N, Vasilchenko O, Prozorovskaya K, et al. Preeclampsia: The Interplay between Oxygen-Sensitive miRNAs and Erythropoietin. J Clin Med. 2020 Feb 20;9(2):574.

200. Nizyaeva N V., Kulikova G V., Nagovitsyna MN, Kan NE, Prozorovskaya KN, Shchegolev AI. Change in OncomicroRNA Expression in the Placenta during Preeclampsia. Bull Exp Biol Med. 2018 Oct 23;165(6):793–7.

201. Artemieva KA, Nizyaeva N V., Baev OR, Romanov AYu, Khlestova G V., Boltovskaya MN, et al. Regulation of the Placental Renin-Angiotensin-Aldosterone System in Early- and Late-Onset Preeclampsia. Dokl Biochem Biophys. 2022 Dec 29;507(1):256–63.

202. Hromadnikova I, Kotlabova K, Ivankova K, Krofta L. First trimester screening of circulating C19MC microRNAs and the evaluation of their potential to predict the onset of preeclampsia and IUGR. PLoS One. 2017 Feb 9;12(2):e0171756.

203. Hromadnikova I, Kotlabova K, Hympanova L, Krofta L. Gestational hypertension, preeclampsia and intrauterine growth restriction induce dysregulation of cardiovascular and cerebrovascular disease associated microRNAs in maternal whole peripheral blood. Thromb Res. 2016 Jan;137:126–40.

204. Hromadnikova I, Kotlabova K, Hympanova L, Krofta L. Cardiovascular and Cerebrovascular Disease Associated microRNAs Are Dysregulated in Placental Tissues Affected with Gestational Hypertension, Preeclampsia and Intrauterine Growth Restriction. PLoS One. 2015 Sep 22;10(9):e0138383.

205. Hromadnikova I, Kotlabova K, Ondrackova M, Pirkova P, Kestlerova A, Novotna V, et al. Expression Profile of C19MC microRNAs in Placental Tissue in Pregnancy-Related Complications. DNA Cell Biol. 2015 Jun;34(6):437–57.

206. Hromadnikova I, Kotlabova K, Ondrackova M, Kestlerova A, Novotna V, Hympanova L, et al. Circulating C19MC MicroRNAs in Preeclampsia, Gestational Hypertension, and Fetal Growth Restriction. Mediators Inflamm. 2013;2013:1–12.

207. Ghafari A, Lessan pezeshki M, Saffari M. P0069MICRO RNA 155, 210, 494, 29B and 34A expression profile in preeclampsia and normal pregnancies. Nephrology Dialysis Transplantation. 2020 Jun 1;35(Supplement_3).

208. Nejad RMA, Saeidi K, Gharbi S, Salari Z, Saleh-Gohari N. Quantification of circulating miR-517c-3p and miR-210-

3p levels in preeclampsia. Pregnancy Hypertens. 2019 Apr;16:75–8.

209. Azizi F, Saleh Gargari S, Asadi Shahmirzadi S, Dodange F, Amiri V, Mirfakhraie R, et al. Evaluation of Placental mir-155-5p and Long Non-coding RNA sONE Expression in Patients with Severe Pre-eclampsia. Int J Mol Cell Med. 2017;6(1):22–30.

210. Amin-Beidokhti M, Sadeghi H, Pirjani R, Gachkar L, Gholami M, Mirfakhraie R. Differential expression of <i>Hsa-miR-517a/b</i> in placental tissue may contribute to the pathogenesis of preeclampsia. Journal of the Turkish-German Gynecological Association. 2021 Dec 1;22(4):273–8.

211. Yousefzadeh Y, Soltani-Zangbar MS, Kalafi L, Tarbiat A, Shahmohammadi Farid S, Aghebati-Maleki L, et al. Evaluation of CD39, CD73, HIF-1α, and their related miRNAs expression in decidua of preeclampsia cases compared to healthy pregnant women. Mol Biol Rep. 2022 Nov 1;49(11):10183–93.

212. Sandrim V, Luizon M, Palei A, Tanus-Santos J, Cavalli R. Circulating microRNA expression profiles in pre-eclampsia: evidence of increased miR-885-5p levels. BJOG. 2016 Dec;123(13):2120–8.

213. Campos CB, Marques TM, Pereira RW, Sandrim VC. Reduced circulating miR-196b levels is associated with preeclampsia. Pregnancy Hypertension: An International Journal of Women's Cardiovascular Health. 2014 Jan;4(1):11–3.

214. Sandrim VC, Diniz S, Eleuterio NM, Gomes KB, Dusse LMS, Cavalli RC. Higher levels of circulating TIMP-4 in preeclampsia is strongly associated with clinical parameters and microRNA. Clin Exp Hypertens. 2018 Oct 3;40(7):609–12.

215. Luizon MR, Conceição IMCA, Viana-Mattioli S, Caldeira-Dias M, Cavalli RC, Sandrim VC. Circulating MicroRNAs in the Second Trimester From Pregnant Women Who Subsequently Developed Preeclampsia: Potential Candidates as Predictive Biomarkers and Pathway Analysis for Target Genes of miR-204-5p. Front Physiol. 2021 Sep 22;12.

216. Sekar D, Lakshmanan G, Mani P, Biruntha M. Methylation-dependent circulating microRNA 510 in preeclampsia patients. Hypertension Research. 2019 Oct 21;42(10):1647–8.

217. Sharma C, Purohit P, Khokhar M, Modi A, Singh P, Shekhar S, et al. A clinical and in-silico study exploring the association of CASP-3, NF-kB, miR-187, and miR-146 in pre-eclampsia. Hypertens Pregnancy. 2021 Oct 2;40(4):288–302.

218. Pallavi Arora, Sankat Mochan, Sunil Kumar Gupta, Neerja Rani, Pallavi Kshetrapal, Sadanand Dwivedi, et al. MicroRNA-22, Specificity protein-1 and Cystathionine β-synthase in early onset Preeclampsia: significance in trophoblast invasion. BioRxiv [Internet]. 2023; Available from: https://doi.org/10.1101/2023.03.08.531738

219. Gopi K. Micro RNA210 expression in pregnancies with preeclampsia. Bioinformation. 2023 Mar 31;19(3):319.

220. Vijayan V, Kannan R, Subhashini Y, Tarakeswari S, Reddy BR, Bangaraiahgari R, et al. QUANTIFICATION OF VARIED MIRNAS IN EARLY AND LATE-ONSET PREECLAMPSIA COMPLICATING PREGNANCIES IN SOUTH INDIAN WOMEN-A COHORT STUDY. International Journal of Pharmaceutical Sciences and Research 5484 IJPSR [Internet]. 2023;14(11):5484–92. Available from: https://doi.org/10.13040/IJPSR.0975-8232.14

221. Ospina-Prieto S, Chaiwangyen W, Herrmann J, Groten T, Schleussner E, Markert UR, et al. MicroRNA-141 is upregulated in preeclamptic placentae and regulates trophoblast invasion and intercellular communication. Translational Research. 2016 Jun;172:61–72.

222. Noack F, Ribbat-Idel J, Thorns C, Chiriac A, Axt-Fliedner R, Diedrich K, et al. miRNA expression profiling in formalin-fixed and paraffin-embedded placental tissue samples from pregnancies with severe preeclampsia. J Perinat Med. 2011 Jan 1;39(3).

223. Kim JH, Kim JY, Park M, Kim S, Kim T, Kim J, et al. NF-κB-dependent miR-31/155 biogenesis is essential for TNF-α-induced impairment of endothelial progenitor cell function. Exp Mol Med. 2020 Aug 7;52(8):1298–309.

224. Choi SY, Yun J, Lee OJ, Han HS, Yeo MK, Lee MA, et al. MicroRNA expression profiles in placenta with severe preeclampsia using a PNA-based microarray. Placenta. 2013 Sep;34(9):799–804.

225. Kim S, Lee KS, Choi S, Kim J, Lee DK, Park M, et al. NF-κB–responsive miRNA-31-5p elicits endothelial dysfunction associated with preeclampsia via down-regulation of endothelial nitric-oxide synthase. Journal of Biological Chemistry. 2018 Dec;293(49):18989–9000.

226. Awamleh Z, Gloor GB, Han VKM. Placental microRNAs in pregnancies with early onset intrauterine growth

restriction and preeclampsia: potential impact on gene expression and pathophysiology. BMC Med Genomics. 2019 Dec 27;12(1):91.

227. Murphy MSQ, Casselman RC, Tayade C, Smith GN. Differential expression of plasma microRNA in preeclamptic patients at delivery and 1 year postpartum. Am J Obstet Gynecol. 2015 Sep;213(3):367.e1-367.e9.

228. Brkić J, Dunk C, O'Brien J, Fu G, Nadeem L, Wang Y ling, et al. MicroRNA-218-5p Promotes Endovascular Trophoblast Differentiation and Spiral Artery Remodeling. Molecular Therapy. 2018 Sep;26(9):2189–205.

229. Lázár L, Nagy B, Molvarec A, Szarka A, Rigó J. Role of hsa-miR-325 in the etiopathology of preeclampsia. Mol Med Rep. 2012 Sep;6(3):597–600.

230. Lázár L, Nagy B, Morvarec A, Rigó J. The Correlation of Circulating Cell-Free DNA, Cell-Free Fetal DNA and MicroRNA 325 Levels to Clinical Characteristics and Laboratory Parameters in Pre-eclampsia. In: Circulating Nucleic Acids in Plasma and Serum. Dordrecht: Springer Netherlands; 2010. p. 153–6.

231. Biró O, Fóthi Á, Alasztics B, Nagy B, Orbán TI, Rigó J. Circulating exosomal and Argonaute-bound microRNAs in preeclampsia. Gene. 2019 Apr;692:138–44.

232. Martinez-Fierro ML, Garza-Veloz I. Analysis of Circulating microRNA Signatures and Preeclampsia Development. Cells. 2021 Apr 24;10(5):1003.

233. Martinez-Fierro ML, Carrillo-Arriaga JG, Luevano M, Lugo-Trampe A, Delgado-Enciso I, Rodriguez-Sanchez IP, et al. Serum levels of miR-628-3p and miR-628-5p during the early pregnancy are increased in women who subsequently develop preeclampsia. Pregnancy Hypertens. 2019 Apr;16:120–5.

234. Martinez-Fierro ML, Garza-Veloz I, Gutierrez-Arteaga C, Delgado-Enciso I, Barbosa-Cisneros OY, Flores-Morales V, et al. Circulating levels of specific members of chromosome 19 microRNA cluster are associated with preeclampsia development. Arch Gynecol Obstet. 2018 Feb 1;297(2):365–71.

235. Licini C, Avellini C, Picchiassi E, Mensà E, Fantone S, Ramini D, et al. Pre-eclampsia predictive ability of maternal miR-125b: a clinical and experimental study. Translational Research. 2021 Feb;228:13–27.

236. Ura B, Feriotto G, Monasta L, Bilel S, Zweyer M, Celeghini C. Potential role of circulating microRNAs as early markers of preeclampsia. Taiwan J Obstet Gynecol. 2014 Jun;53(2):232–4.

237. Luque A, Farwati A, Crovetto F, Crispi F, Figueras F, Gratacós E, et al. Usefulness of circulating microRNAs for the prediction of early preeclampsia at first-trimester of pregnancy. Sci Rep. 2014 May 8;4(1):4882.

238. Ishibashi O, Ohkuchi A, Ali MdM, Kurashina R, Luo SS, Ishikawa T, et al. Hydroxysteroid (17-β) Dehydrogenase 1 Is Dysregulated by Mir-210 and Mir-518c That Are Aberrantly Expressed in Preeclamptic Placentas. Hypertension. 2012 Feb;59(2):265–73.

239. Miura K, Higashijima A, Murakami Y, Tsukamoto O, Hasegawa Y, Abe S, et al. Circulating chromosome 19 miRNA cluster microRNAs in pregnant women with severe pre-eclampsia. Journal of Obstetrics and Gynaecology Research. 2015 Oct;41(10):1526–32.

240. Nunode M, Hayashi M, Nagayasu Y, Sawada M, Nakamura M, Sano T, et al. miR-515-5p suppresses trophoblast cell invasion and proliferation through XIAP regulation in preeclampsia. Mol Cell Endocrinol. 2023 Jan;559:111779.

241. Whigham CA, MacDonald TM, Walker SP, Hiscock R, Hannan NJ, Pritchard N, et al. MicroRNAs 363 and 149 are differentially expressed in the maternal circulation preceding a diagnosis of preeclampsia. Sci Rep. 2020 Oct 22;10(1):18077.

242. Wang Y, Lumbers ER, Arthurs AL, de Meaultsart CC, Mathe A, Avery-Kiejda KA, et al. Regulation of the human placental (pro)renin receptor-prorenin-angiotensin system by microRNAs. MHR: Basic science of reproductive medicine. 2018 Jul 28;

243. Jairajpuri DS, Malalla ZH, Sarray S, Mahmood N. Analysis of differential expression of hypoxia-inducible microRNA-210 gene targets in mild and severe preeclamptic patients. Noncoding RNA Res. 2021 Mar;6(1):51–7.

244. Jairajpuri DS, Malalla ZH, Mahmood N, Almawi WY. Circulating microRNA expression as predictor of preeclampsia and its severity. Gene. 2017 Sep;627:543–8.

245. Witvrouwen I, Mannaerts D, Ratajczak J, Boeren E, Faes E, Van Craenenbroeck AH, et al. MicroRNAs targeting VEGF are related to vascular dysfunction in preeclampsia. Biosci Rep. 2021 Aug 27;41(8).

246. Munaut C, Tebache L, Blacher S, Noël A, Nisolle M, Chantraine F. Dysregulated circulating miRNAs in preeclampsia. Biomed Rep. 2016 Dec;5(6):686–92.

247. Kolkova Z, Holubekova V, Grendar M, Nachajova M, Zubor P, Pribulova T, et al. Association of Circulating miRNA Expression with Preeclampsia, Its Onset, and Severity. Diagnostics. 2021 Mar 8;11(3):476.

248. Lasabová Z, Vazan M, Zibolenova J, Svecova I. Overexpression of miR-21 and miR-122 in preeclamptic placentas. Neuro Endocrinol Lett. 2015 Dec;36(7):695–9.

249. Mavreli D, Lykoudi A, Lambrou G, Papaioannou G, Vrachnis N, Kalantaridou S, et al. Deep Sequencing Identified Dysregulated Circulating MicroRNAs in Late Onset Preeclampsia. In Vivo (Brooklyn). 2020 Aug 31;34(5):2317–24.

250. Lykoudi A, Kolialexi A, Lambrou GI, Braoudaki M, Siristatidis C, Papaioanou GK, et al. Dysregulated placental microRNAs in Early and Late onset Preeclampsia. Placenta. 2018 Jan;61:24–32.

251. Ali Z, Zafar U, Zaki S, Ahmad S, Khaliq S, Lone KP. Expression levels of MiRNA-16, SURVIVIN and TP53 in Preeclamptic and Normotensive women. J Pak Med Assoc. 2021 Sep;71(9):2208–13.

252. Ali Z, Zafar U, Tauseef A, Zaki S, Khaliq S. MICRO RNA 182-3-P, 519-D-5P, 378-3P AS NON-INVASIVE PREDICTORS OF PREECLAMPSIA. Journal of Ayub Medical College Abbottabad. 2023 Aug 10;35(3).

253. Wang C, Tsai P, Chen T, Tsai H, Kuo P, Su M. Elevated miR‐200a and miR‐141 inhibit endocrine gland‐derived vascular endothelial growth factor expression and ciliogenesis in preeclampsia. J Physiol. 2019 Jun 22;597(12):3069–83.

254. Tsai PY, Li SH, Chen WN, Tsai HL, Su MT. Differential miR-346 and miR-582-3p Expression in Association with Selected Maternal and Fetal Complications. Int J Mol Sci. 2017 Jul 19;18(7):1570.

255. Khaliq OP, Murugesan S, Moodley J, Mackraj I. Differential expression of miRNAs are associated with the insulin signaling pathway in preeclampsia and gestational hypertension. Clin Exp Hypertens. 2018 Nov 17;40(8):744–51.

256. Soobryan N, Kumar A, Moodley J, Mackraj I. The role and expression of pro/antiangiogenic factors and microRNAs in gestational hypertension and pre-eclampsia. European Journal of Obstetrics & Gynecology and Reproductive Biology. 2023 Nov;290:38–42.

257. Doridot L, Houry D, Gaillard H, Chelbi ST, Barbaux S, Vaiman D. miR-34a expression, epigenetic regulation, and function in human placental diseases. Epigenetics. 2014 Jan 30;9(1):142–51.

258. Lip S V., Boekschoten M V., Hooiveld GJ, van Pampus MG, Scherjon SA, Plösch T, et al. Early-onset preeclampsia, plasma microRNAs, and endothelial cell function. Am J Obstet Gynecol. 2020 May;222(5):497.e1-497.e12.

259. Weedon-Fekjær MS, Sheng Y, Sugulle M, Johnsen GM, Herse F, Redman CW, et al. Placental miR-1301 is dysregulated in early-onset preeclampsia and inversely correlated with maternal circulating leptin. Placenta. 2014 Sep;35(9):709–17.

260. Jelena M, Sopić M, Joksić I, Zmrzljak UP, Karadžov-Orlić N, Košir R, et al. Placenta-specific plasma miR518b is a potential biomarker for preeclampsia. Clin Biochem. 2020 May;79:28–33.

261. Lalevée S, Lapaire O, Bühler M. miR455 is linked to hypoxia signaling and is deregulated in preeclampsia. Cell Death Dis. 2014 Sep 4;5(9):e1408–e1408.

262. Kim S, Park M, Kim JY, Kim T, Hwang J, Ha KS, et al. Circulating miRNAs Associated with Dysregulated Vascular and Trophoblast Function as Target-Based Diagnostic Biomarkers for Preeclampsia. Cells. 2020 Aug 31;9(9):2003.

263. Trongpisutsak A, Phupong V. Prediction of preeclampsia using a combination of serum micro RNA-210 and uterine artery Doppler ultrasound. Sci Prog. 2021 Jul 25;104(3):003685042110368.
